# Trace Adsorptive Removal of PFAS from Water by Optimizing the UiO‐66 MOF Interface

**DOI:** 10.1002/adma.202413120

**Published:** 2024-11-21

**Authors:** Nebojša Ilić, Kui Tan, Felix Mayr, Shujin Hou, Benedikt M. Aumeier, Eder Moisés Cedeño Morales, Uwe Hübner, Jennifer Cookman, Andreas Schneemann, Alessio Gagliardi, Jörg E. Drewes, Roland A. Fischer, Soumya Mukherjee

**Affiliations:** ^1^ Chair of Urban Water Systems Engineering Technical University of Munich Am Coulombwall 3 85748 Garching Germany; ^2^ Department of Chemistry University of North Texas 1155 Union Cir Denton TX 76203 USA; ^3^ Chair of Simulation of Nanosystems for Energy Conversion, Department of Electrical Engineering, TUM School of Computation, Information and Technology Technical University of Munich Hans‐Piloty‐Straße 1 85748 Garching Germany; ^4^ Chair of Inorganic and Metal‐Organic Chemistry, Catalysis Research Center School of Natural Sciences Technical University of Munich 85748 Garching Germany; ^5^ Xylem Services GmbH Boschstraße 4–14 32051 Herford Germany; ^6^ Bernal Institute, Department of Chemical Sciences University of Limerick Limerick V94 T9PX Ireland; ^7^ Inorganic Chemistry I Technische Universität Dresden Bergstraße 66 01069 Dresden Germany

**Keywords:** functional porous materials, materials chemistry, metal–organic frameworks, MOF–polymer composites, poly‐ and perfluoroalkyl substances (PFAS), water purification

## Abstract

The confluence of pervasiveness, bioaccumulation, and toxicity in freshwater contaminants presents an environmental threat second to none. Exemplifying this trifecta, per‐ and polyfluoroalkyl substances (PFAS) present an alarming hazard among the emerging contaminants. State‐of‐the‐art PFAS adsorbents used in drinking water treatment, namely, activated carbons and ion‐exchange resins, are handicapped by low adsorption capacity, competitive adsorption, and/or slow kinetics. To overcome these shortcomings, metal–organic frameworks (MOFs) with tailored pore size, surface, and pore chemistry are promising alternatives. Thanks to the compositional modularity of MOFs and polymer–MOF composites, herein we report on a series of water‐stable zirconium carboxylate MOFs and their low‐cost polymer‐grafted composites as C_8_–PFAS adsorbents with benchmark kinetics and “parts per billion” removal efficiencies. Bespoke insights into the structure–function relationships of PFAS adsorbents are obtained by leveraging interfacial design principles on solid sorbents, creating a synergy between the extrinsic particle surfaces and intrinsic molecular building blocks.

## Graphical abstract

Using a prototypal platform of metal–organic frameworks (MOFs) and polymer–MOF hybrid sorbents, an optimal combination of surface hydrophobicity and strong electrostatics enables “parts per billion” removal of per‐ and polyfluoroalkyl substances (PFAS), with record‐high kinetics and efficient sorbent regeneration. The expendable nature of guest‐accessible porosity for PFAS removal is established, underpinning a counterintuitive perspective on future adsorbent design.

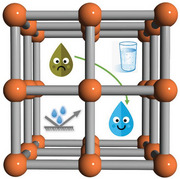

## 1 Introduction

Surging industrialization implies increased exposure of mankind to water resources contaminated with industrial pollutants. One group of industrial pollutants gaining recent attention is a class of anthropogenic organic chemicals, per‐ and polyfluoroalkyl substances (PFAS), due to their widespread use as surface coatings, and resistance to degradation.^[^ ^1^ ^]^ They consist of hydrophobic, fully (per‐) or partly (poly‐)fluorinated, linear or branched alkyl chains (containing strong carbon–fluorine (C─F) bonds, average bond energy ≈ 485 kJ mol^−1^),^[^ ^2^ ^]^ connected to different polar functional head groups (mainly carboxylate, sulfonate, or phosphate). Perfluoroalkyl moieties of PFAS provide ultralow surface energy which results in high hydrophobicity and oleophilicity. This property is “best fit” to deliver water‐ and stain‐proof, nonsticky surfactants/polymers, used in numerous industrial/consumer applications, e.g., textiles and leather, firefighting foams, metal plating, and paper packaging. Nonetheless, prolonged human exposure to drinking waterborne PFAS, such as perfluoroalkyl carboxylic acids (PFCAs)—perfluorooctanoic acid (PFOA) and hexafluoropropylene oxide dimer acid and its ammonium salt (GenX)—as well as perfluoroalkyl sulfonic acids (PFSAs)—perfluorooctanesulfonic acid (PFOS) (Scheme S1, Supporting Information) even at trace levels causes human bioaccumulation with multiple adverse effects. These include hepatotoxicity, tumor induction, developmental toxicity, immunotoxicity, neurotoxicity, and endocrine disruption among others.^[^ ^3^ ^]^ Under this backdrop, several PFAS have been banned or their use is limited, with PFOS joining the restriction list (Annexure B) of the Stockholm convention on Persistent Organic Pollutants (POPs) in 2009 and PFOA the elimination list (Annexure A) in 2019, followed by PFHxS (perfluorohexanesulfonic acid) in 2022.^[^ ^4^ ^]^ Further regulations are applied (or are in the pipeline) to a wide variety of PFAS species through the European Registration, Evaluation, Authorisation and Restriction of Chemicals (REACH) regulation, but even more importantly through the EU Drinking Water Directive, which limits the total amount of PFAS in supplied drinking water to 500 ng L^−1^, while a sum of selected 20 PFAS is limited to 100 ng L^−1^. The US Environmental Protecting Agency (EPA) also announced a new national drinking water regulation in April 2024, limiting the amount of 5 PFAS in supplied drinking water, with PFOA and PFOS being limited to 4 ng L^−1^, and PFNA, PFHxS, HFPO‐DA ((aka. GenX) to 10 ng L^−1^.^[^ ^5^ ^]^ These limits are only expected to become more stringent in the future, as analytical capabilities to detect and quantify PFAS improve.^[^ ^6^ ^]^

Physicochemical properties of PFAS, particularly their chemical inertness, challenge their removal from drinking water, and their efficient removal, remain an active issue in drinking water treatment. The EPA recommends three separation technologies for use in drinking water.^[^ ^7^ ^]^ High‐pressure membrane filtration (i.e., reverse osmosis/nanofiltration) is a method that effectively separates PFAS into a liquid concentrate stream while producing a permeate stream free of PFAS (both short chain and long chain are separated well). The use of sorbents, namely, granular activated carbon (GAC) and/or ion‐exchange resins (IXR), is recommended for passive PFAS capture, potentially offering a significant reduction in operating costs of the systems.^[^ ^8^ ^]^ While all three are valid treatment options, there are several downsides: A) membrane filtration is a high‐cost method both to install and operate under high pressures; B) activated carbon needs frequent bed exchanges due to fast PFAS breakthrough times and high competitiveness of other present contaminants;^[^ ^9^ ^]^ C) IXR are difficult to regenerate and costly to replace, and are still often disposed of via incineration alike GAC while requiring complex chemical syntheses to produce.^[^ ^8^, ^10^ ^]^ These pitfalls and the observed ineptness of GAC and IXR to effectively capture short‐chain PFAS foster the search for alternative energy‐efficient adsorbents that represent a high degree of selectivity,^[^ ^11^ ^]^ and rely upon regenerable adsorption as a technology that embodies green circular economy for a sustainable future.^[^ ^12^ ^]^

Among emerging energy‐efficient and selective adsorbents, reticular porous coordination networks, also known as MOFs, have gained particular significance in hydrocarbon adsorption technologies, largely due to their tuneable porosity and guest‐accessible porous nanospace.^[^ ^13^ ^]^ Modularity of these porous solids can potentially offer an adsorbent design paradigm to meet the PFAS remediation specifications from contaminated ground and surface waters.^[^ ^14^ ^]^

Considering the high hydrophobicity of PFAS, leveraging the compositional modularity of MOFs, the design of hydrophobic MOFs has shown early signs of PFAS capture in terms of high saturation capacities (comparable to GAC). However, research in this area is at an underexplored niche.^[^ ^14^, ^15^ ^]^ Nonetheless, rolling out early performance metrics, the simultaneous control of surface and pore hydrophobicity has resulted in a few select MOFs to be benchmarked as the most hydrophobic materials reported thus far,^[^ ^16^ ^]^ therefore, offering an edge over other physisorbents.^[^ ^17^ ^]^ However, to design effective MOF adsorbents for PFAS removal, the nature of PFAS adsorption and the underlying fundamental forces must be identified first and simply put; this remains an unmet challenge thus far. In this work, we explore the adsorption performance toward 11 different PFAS contaminants by a library of 4 isostructural MOFs, each functionalized with distinct functional groups (FG): hydrogen (electronically neutral but key to facilitate hydrogen bonding), nitro (a π‐electron‐deficient group), amino (a π‐electron‐rich group), and fluorines (multiple F atoms, each with a σ‐electron‐deficient group). Since the parent MOF platform, namely, the **UiO‐66** family of MOFs^[^ ^18^ ^]^ is well known for its water stability^[^ ^19^ ^]^ and was previously studied for PFAS capture from high initial concentration,^[^ ^20^ ^]^ it serves as an ideal sorbent prototype to examine trace PFAS removal performances (from initial concentrations of few “parts per billion,” ppb), alongside deciphering the structure–function relationships. Beyond controlling the MOF micropore chemistry, we carefully scrutinize the impact of interfacial modulation of hydrophobicity and electrostatics upon trace PFAS removal performance by probing two organic polymer–MOF composite families. We approach this through a wide spectrum of complementary techniques, including trace removal/regeneration experiments, computational analyses, spectroscopic studies, and microscopic insights into the experimental performance parameters.

## 2 Experimental Section

### 2.1 Materials

All the reagents, starting materials, and solvents were commercially purchased from Sigma–Aldrich (Merck), and were used without further purification. Detailed syntheses of the MOFs and MOF composites are included in the Supporting Information. Eleven different PFAS were used in this study (7 PFCAs, 2 PFSAs, and 2 perfluoroalkyl ether carboxylic acids (PFECAs), with three PFAS in focus (Scheme S1, Supporting Information) as the most discussed species in literature (PFOA, PFOS, and GenX). A full list of PFAS chemicals used can be found in Table S1 (Supporting Information). Type 1 ultrapure water was used exclusively in preparation of all experiments and stock solutions, obtained from a Sartorius Arium Pro water purification system. Thermo Fischer Scientific Orion Star A214 series benchtop pH/ISE meter and Ross Ultra glass combination pH electrode were purchased directly from Thermo Fisher Scientific and used for all pH measurements. Reagent grade sodium chloride (NaCl), liquid chromatography‐mass spectrometry (LC‐MS) grade methanol (CH_3_OH) and 99% formic acid (HCOOH) were acquired from VWR. Polypropylene (PP) labware was used exclusively in preparation of all experiments and measurements (volumetric flasks, beakers, wide‐neck bottles, funnels, pipette tips, and syringe filters). Different volume polypropylene labware was purchased from VWR, while the syringes (2 mL) and PP‐frit syringe filters (13 mm, 0.22 µm) were purchased from BGB Analytik, Germany.

### 2.2 General Characterizations and Physical Measurements

All adsorption experiments discussed in this work were conducted in a water bath shaker (JULABO SW22) set to 200 RPM and with a fixed temperature setting of 20 ± 1 °C. All adsorption samples were filtered through a syringe filter immediately upon sampling by capturing 2 mL in a syringe, discarding the first five drops of filtrate, and capturing the rest in Eppendorf vials for later quantification. While random control showed no issues with measuring samples even after 2 months of storage in the refrigerator, the general principle was to measure samples as quickly as possible after obtaining them.

### 2.3 Experimental Procedures

Five different adsorption experiments were designed and conducted, serving different purposes. First, initial PFAS adsorption performance was screened with batch removal experiments for PFOA, PFOS, and GenX to identify which materials to study further. Adsorption kinetics were then determined for selected materials, to determine equilibrium times for further tests. Following these, removal of 11 different PFAS at environmentally relevant concentrations was examined, subsequently followed by isotherm experiments to observe changes in capacity which occur due to different surface chemistry and applied coating. Finally, regeneration of best‐performing adsorbents was evaluated by creating microscale adsorption columns. MOF particles were loaded on polypropylene syringe filters, and multiple adsorption and regeneration cycles were performed with the produced microscale columns (in duplicate). All batch experiments (excluding kinetics) were conducted in triplicate, with triplicate blanks operated in parallel (vials without adsorbent added) that were treated and filtered in the same manner to account for any PFAS losses due to sample processing. The screening batch adsorption results were deduced from a total of 4–5 repetitions from two separate experiments, for improved certainty of screening results. More in‐depth reasoning and experimental details for all experiments are presented in the Supporting Information. **Table** **1** presents an overview of experimental conditions for all conducted experiments, as well as the PFAS species studied in each experiment.

### 2.4 Instrumental Analysis

PFAS concentrations were determined with an LC/MS–MS system (Agilent 1260 Infinity, ABSciex Qtrap 5500). The analytical method was developed with consideration of the German standard method DIN 38407–42, and the US Environmental Protection Agency Method 537.1. A complete list of PFAS species and *m*/*z* ratios measured by the system, analytical and internal PFAS standards, and a more detailed explanation of the analytical method can be found in the Supporting Information. A separation column from Waters (XSelect HSS T3, 100 Å, 3.5 µm, 2.1 mm × 100 mm) and the delay column from Agilent (ZORBAX Eclipse plus; C18 95 Å, 3.5 µm, 4.6 mm × 30 mm) were used. The established method had an effective detection range between 5 and 5000 ng L^−1^, i.e., 5 to 5000 ppt. Selected total ion chromatogram (TIC) and mass spectra before and after adsorption for all 11 PFAS are presented in Figures S33 and S34 (Supporting Information) (parts 1 and 2 available for each). Polypropylene LC vials and polyethylene snap‐on caps from Agilent were used in preparation of all LC‐MS samples and calibration standards. More information on the quantification of PFAS using LC/MS‐MS can be found in the Supporting Information.

## 3 Results and Discussion

**Figure 1:**
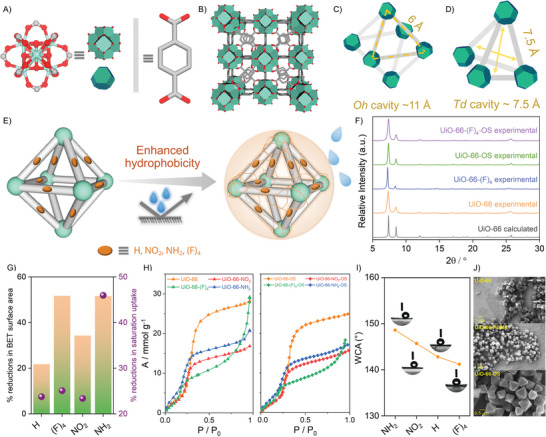
Sorbent structures and characterization. A) The molecular building blocks sustaining the **UiO‐66‐X** (X = H, (F)_4_, NH_2_, and NO_2_) structures; an octahedral Zr(IV)–oxo metal cluster (left) and one of the terephthalate linker derivatives (X = H, NO_2_, NH_2_, and (F)_4_) (Zr = olive green; O = crimson red; C = gray; H atoms and *μ* ^3^‐O bridges are omitted for clarity). B) Polyhedral illustration of the sorbent architecture; turquoise truncated octahedra and gray pillars correspond to the Zr_6_–oxo clusters and (X‐)terephthalate linkers that form C) the 11 Å octahedral cage, enclosing a pore aperture of 6 Å, and D) a tetrahedral 7.5 Å cage. E) Schematic illustration of the adopted polymer (PDMS and/or OS) protection strategy, primed to induce surface hydrophobicity. F) Powder X‐ray diffractograms recorded on **UiO‐66‐X** and the corresponding OS protected **UiO‐66‐X‐OS** (X = H and (F)_4_) samples postactivation, plotted alongside the calculated **UiO‐66** powder X‐ray pattern reported in the Cambridge Structural Database Refcode RUBTAK03.^[^ ^27^ ^]^ G) Upon OS treatment, percent reductions of the Brunauer–Emmett–Teller (BET) surface areas (*Y*_1_‐axis), and those of the nitrogen saturation uptakes at 77 K (*Y*_2_‐axis). H) Comparison of H_2_O vapor adsorption isotherms (recorded at 298 K) for all the four **UiO‐66‐X** MOFs, before and after the OS treatment. I) Static water contact angles for the four OS‐treated **UiO‐66** derivatives. Insets include the water droplet pictures used for the respective measurements. J) Field‐emission scanning electron microscopy (FE‐SEM) micrographs of the observed morphologies for **UiO‐66, UiO‐66‐PDMS**, and **UiO‐66‐OS** crystallites, following each of the synthesis.

Taking cognizance of the water stability and isostructural nature in the prototypical Zr(IV)‐carboxylate **UiO‐66** family of MOFs,^[^ ^18^ ^]^ here we critically interrogate PFAS removal in four distinctly tagged **UiO‐66** variants: **UiO‐66‐X** (defect‐free, unsubstituted),^[^ ^21^ ^]^ [Zr_6_(*μ*_3_‐O)_4_(*μ*_3_‐OH)_4_(BDC)_6_]*_n_* (BDC = 1,4‐benzenedicarboxylate) and its functionalized analogs **UiO‐66‐X**, with X = NH_2_,^[^ ^22^ ^]^ NO_2_,^[^ ^22^ ^]^ and ─ (F)_4_ (**Figure** **1**; Scheme S2, Supporting Information).^[^ ^23^ ^]^ The structures crystallize in *Fm‐3m* space group (sodium chloride type), where the secondary building units (SBU) occupy the unit cell vertices and the face centers (Figure 1B), whereas the center of the cell (by symmetry, the midpoint of each edge) presents an octahedral cage of 11 Å (each with triangular windows of 6 Å) (Figure 1C), and a tetrahedral cage of 7.5 Å apertures (Figure 1D). The polycrystalline morphology and hydrophilicity (experimental water contact angle (WCA) ≈ 0° for **UiO‐66‐X**)^[^ ^24^ ^]^ of these MOFs were compared against the two hydrophobic polymer–MOF composite families, **UiO‐66‐X‐PDMS** and **UiO‐66‐X‐OS** that we prepared (Figure 1F,I),^[^ ^25^ ^]^ by low‐cost, post‐synthetic polydimethylsiloxane (PDMS) and organosilicone (OS) treatment, respectively (Figure 1E–I; see Figures S1,S2, and S6–S32 in the Supporting Information for key properties like synthesis and detailed characterization).^[^ ^25^, ^26^ ^]^ It is worth highlighting that **UiO‐66‐X‐OS** is a previously unreported family of composites, enabling us to closely evaluate the interfacial chemistry of its intrinsic micropores and extrinsic particle surfaces, which will, hereinafter, be referred to simply as MOF surfaces.

Predicated upon the well‐known **UiO‐66‐X** (X = H, NO_2_, NH_2_, and (F)_4_) family of isostructural MOFs,^[^ ^18^, ^21^, ^22^, ^23^ ^]^ two families of isostructural polymer–MOF composites, **UiO‐66‐X‐PDMS** and **UiO‐66‐X‐OS**, were studied (Figure 1E,F; Figures S1 and S2, Supporting Information).^[^ ^25^, ^26^ ^]^ Powder X‐ray diffractograms (PXRD) (Figure 1F; Figures S1 and S2, Supporting Information), thermogravimetric analysis (TGA) traces (Figures S5–S7, Supporting Information), the cryogenic (77 K) N_2_ adsorption isotherm‐based A) Brunauer–Emmett–Teller (BET) surface area analyses (Figure 1G; Figures S8–S24 and Table S3, Supporting Information), and B) pore size distribution analyses (Figures S25–S28, Supporting Information) confirm consistency across the materials platform and agreement with the literature.^[^ ^26^ ^]^ Hydrophilicity of the OS‐coated derivatives, **UiO‐66‐X‐OS**, was compared against the hydrophobic **UiO‐66‐X‐PDMS** composites,^[^ ^25^ ^]^ and the hydrophilic **UiO‐66‐X** MOFs^[^ ^26b^ ^]^ by monitoring: A) the pore hydrophilicity signatures (reflected in the water vapor adsorption isotherms (WAI) (Figure 1H; Figure S29, Supporting Information), and B) the surface hydrophobicity signatures (evident from static WCA measurements (Figure 1I; Figure S30, Supporting Information)). Scanning electron microscopy (SEM) micrographs at the precoating and the postcoating stages of the composites were captured to examine the consistency of polycrystalline morphologies, that is, the nature of the crystal habits compared to those of **UiO‐66‐X** (Figure 1J; Figures S31 and S32, Supporting Information). Interestingly, albeit retaining the crystallite morphologies and the pore hydrophilicities (as evident from the similar WAIs), microporosity is significantly reduced in the **UiO‐66‐X‐PDMS** and **UiO‐66‐X‐OS** composites, in stark contrast to the pronounced increase to surface hydrophobicity observed in the composites (Figure 1I; Figure S30, Supporting Information) versus the hydrophilic (WCA ≈ 0°) **UiO‐66‐X** MOFs. With regard to guest‐accessible surface areas, the limited microporosity in the MOFs and/or the derived composites is deemed unlikely to offer porous channels suitable for the capture and/or confinement of large guest molecules such as PFOA and PFOS. In essence, the following study hinges on a key question: is porosity essential for enabling PFAS capture, or could an optimal combination of surface properties suffice?

**Figure 2:**
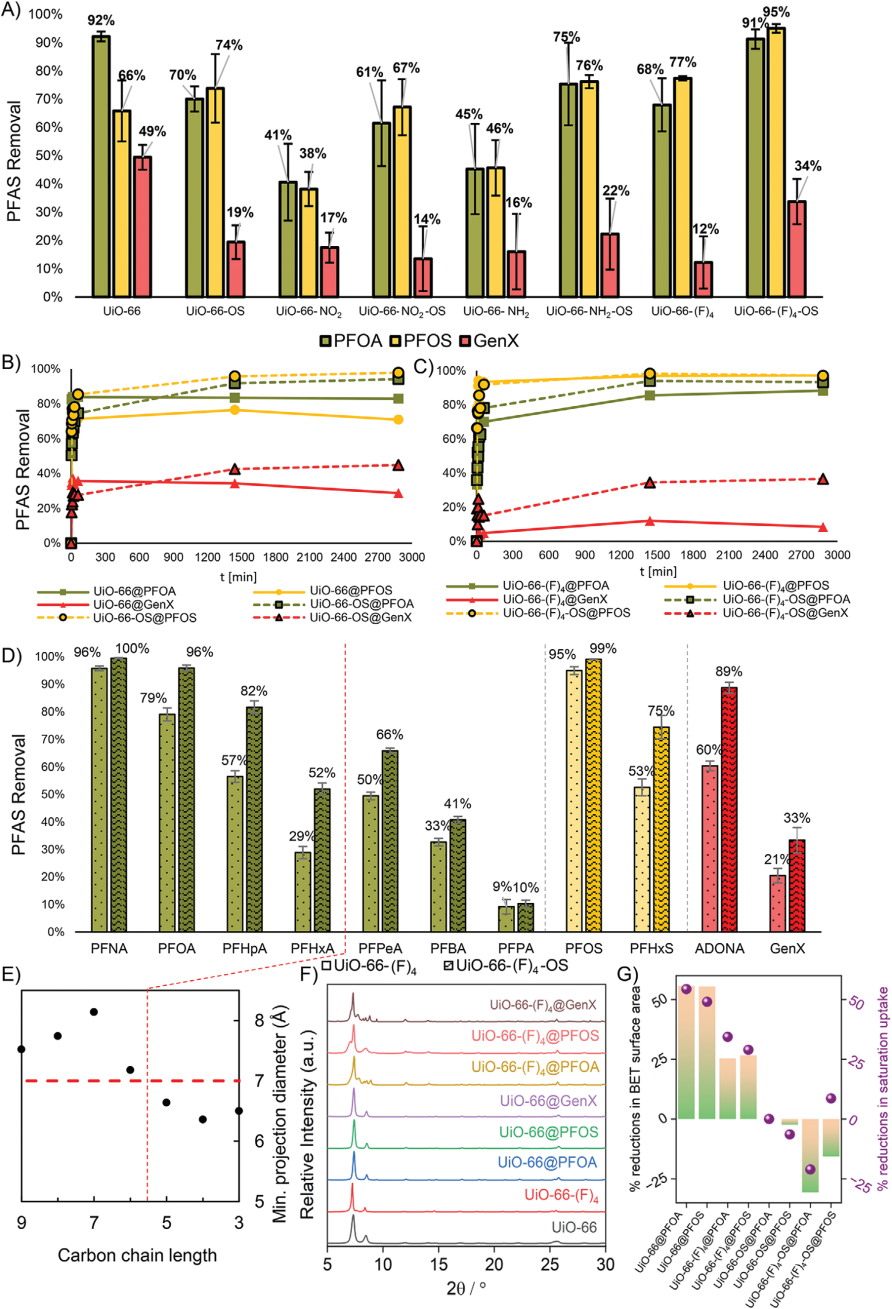
PFAS batch adsorption, removal kinetics, removal at trace level concentrations and associated phase characterizations. A) Batch removal performances exhibited by the four isostructural **UiO‐66‐X** adsorbents, and their four OS‐coated analogs after 24 h of equilibration (starting PFAS concentration ≈ 100 µg L^−1^, i.e., 100 ppb). B) Comparison of adsorption kinetics between **UiO‐66** and **UiO‐66‐OS**. C) Comparison of adsorption kinetics between **UiO‐66‐(F)_4_** and **UiO‐66‐(F)_4_‐OS** (*C*_0_ ≈ 100 µg L^−1^ each PFAS in mixture). D) Adsorption performance comparison between **UiO‐66‐(F)_4_** and **UiO‐66‐(F)_4_‐OS** at environmentally relevant concentrations (*C*_0_ ≈ 2 µg L^−1^) using a stock mixture containing 11 different PFAS species with 48 h equilibration time. E) The calculated projected dynamic radius of the PFCA series using ChemAxon Chemicalize in comparison to the **UiO‐66** pore aperture width anticipated to be at ≈7 Å (horizontal red line). F) **UiO‐66‐X** (X = H and (F)_4_) powder X‐ray diffractograms after synthesis followed by activation, plotted alongside the corresponding post‐PFAS adsorption phases (PFAS: PFOA, PFOS, and GenX). G) Upon PFOA and PFOS saturation, percent reductions of the BET surface areas (*Y*_1_‐axis), and those of the nitrogen saturation uptakes at 77 K (*Y*_2_‐axis).

Recognizing the homogeneously functionalized composite surfaces, we devised a series of experiments to evaluate the adsorption performances for all 12 sorbents that we synthesized, namely the hydrophilic **UiO‐66‐X** MOFs in comparison to the surface‐hydrophobic **UiO‐66‐X‐PDMS** and **UiO‐66‐X‐OS** derivatives (**Figure** **2**A; the Supporting Information dataset). The highest adsorption performance among the pristine MOFs tested was observed in **UiO‐66**, followed by **UiO‐66‐(F)_4_**, as seen in Figure 2A. Further, we observed that **UiO‐66** removed PFOA and GenX best, while **UiO‐66‐(F)_4_** removed most PFOS out of the four tested pristine MOFs, **UiO‐66‐X**. Consistent with the literature,^[^ ^28^ ^]^ two adsorption mechanisms can be considered to be at play. One utilizes electrostatic force and the other fluorine‐based hydrophobic attraction force, with PFCAs presumably getting adsorbed by hydrogen bonding that involves their carboxylate head groups, thus benefiting from the availability of ─H in the pore structure of **UiO‐66**. Conversely, PFOS, due to its long fluorinated tail, adsorbs well in the fluorinated surface of **UiO‐66‐(F)_4_**. This could also provide context for the lower removal performance that we observed for **UiO‐66‐NH_2_** and **UiO‐66‐NO_2_**, where the sizes of the introduced functional groups could reduce access to the remaining hydrogen bonding groups. When applying hydrophobic coating via the PDMS wafer deposition method, we observed major loss of adsorption performance with all four composite materials tested (see the Supporting Information dataset). On the other hand, ultrasonic deposition of OS resulted in improved batch adsorption performance for three out of the four tested MOFs, where only **UiO‐66** displayed lower overall adsorption performance, but showed an increase in PFOS removal. On the whole, these observations indicate that the hydrophobic nature of the OS layer could offer a favorable environment for adsorbing the most hydrophobic guest, PFOS. However, the reduced availability of hydrogens works against this. Conversely, a synergistic effect was observed in **UiO‐66‐(F)_4_**, where the OS coating improved removal of all the PFAS tested.

Further, for all sorbents tested, it is observed that most adsorption occurred in the first 30 min. With **UiO‐66** (Figure 2B), equilibrium was achieved after 3 min, indicating exceptionally fast adsorption kinetics. While **UiO‐66‐(F)_4_** recorded the highest adsorption capacity within the first 30 min, a further ≈10% increase in capture was observed in 24 h (Figure 2C). The nature of adsorption kinetics in **UiO‐66** was found to change after applying the OS coating, displaying slower adsorption, and resembling the adsorption profiles of hydrophobic **UiO‐66‐(F)_4_** and **UiO‐66‐(F)_4_‐OS**. A close‐up of the first 60 min of the kinetic adsorption experiments is provided in Figure S55 (Supporting Information). All four sorbents revealed trends that best fit to pseudosecond order kinetics, where we observed the coefficients of determination, *R* ^2^ values to be 0.95, 0.98, 0.86, and 0.94 for PFOA, and 0.98, 0.99, 0.95, and 0.93 for PFOS, for **UiO‐66**, **UiO‐66‐OS**, **UiO‐66‐(F)_4_**, and **UiO‐66‐(F)_4_‐OS**, respectively. The adsorption kinetics of GenX was found not to correlate well with the adsorption kinetics models.

Trace adsorption performance data for **UiO‐66**, **UiO‐66‐NO_2_**, **UiO‐66‐NH_2_**, and their OS analogs are provided in Figure S56 (Supporting Information). A clear overall downward trend in adsorption performance was noticed with the shortening of the PFAS carbon chain. The best‐performing sorbent in the pristine library, **UiO‐66**, was found to exhibit a notable drop in adsorption performance when coated with OS. On the other hand, **UiO‐66‐NO_2_** and **UiO‐66‐NH_2_** were found to register increased long‐chain PFAS adsorption performances, but reduced adsorption performances toward shorter‐chain PFAS after coating. **UiO‐66‐(F)_4_** revealed a different trend, where we see an improvement in adsorption performance for all PFAS after applying the OS coating (Figure 2D). The observed drop in adsorption performance with shortening of the PFAS carbon chain implies that longer chain lengths and thus stronger fluorine‐based interactions play a major role in facilitating PFAS adsorption. This preferential occupation of adsorption sites by longer chain PFAS can result in poor uptake of shorter chains. However, we observe a slight increase in perfluoropentanoic acid (PFPeA) adsorption compared to perfluorohexanoic acid (PFHxA). This could indicate that the smaller diameter of PFPeA (≈6.64 Å) enables it to partly access the pore channels which are otherwise inaccessible until PFHxA (diameter ≈ 7.18 Å) (Figure 2E). This is in alignment with a pore aperture slightly larger than 6 Å (Figure 1C), likely caused by structural defects that accompany water treatment; 7 Å is adopted in Figure 2E for graphical representation. PFPA, despite its smallest size and the most propensity to enter the **UiO‐66‐X** channels, is poorly adsorbed. This is likely caused by weak interactions between the shortest fluoroalkyl chain and the adsorbent.

Adsorption isotherms can provide further insight into the adsorption performance of the tested materials and bring further understanding of the nature of the adsorption process. We observe that the best isotherm model *R* ^2^ fits depend on the adsorbent and adsorbate pairs; in other words, the nature of the adsorption process varies between materials and PFAS. For example, in **UiO‐66** and **UiO‐66‐OS**, we observe the highest *R* ^2^ for PFOA, while for **UiO‐66‐(F)_4_** and **UiO‐66‐(F)_4_‐OS** we observe highest *R* ^2^ for PFOS, implying a difference in adsorption forces at play, making one or the other the dominant adsorbate. This aligns with the observations made when modeling the adsorption of these PFAS molecules to **UiO‐66‐X** surfaces (vide the following discussion; Figure 4; Figures S60–S67, Supporting Information), where simply put the resting state of the PFOS molecules indicates favorable interactions between **UiO‐66‐(F)_4_** and the fluorinated tails (hydrophobic–hydrophobic interactions, because PFOS features the longest fluoroalkyl group). In contrast, the head groups of PFOA were found to bind to **UiO‐66‐(F)_4_**, implying higher adsorption affinity for PFCAs.^[^ ^29^ ^]^ Opposing the adsorption trends observed with PFOA and PFOS, GenX isotherms revealed much smaller adsorbed amounts underpinning its relatively low adsorption affinity. Based on this fact and our investigation of the corresponding mixture isotherms (Figure S58, Supporting Information), the isotherm shapes were found to be influenced by the presence of the superior‐adsorbing contaminants, PFOA and PFOS. For **UiO‐66**, a sigmoidal (i.e., S‐shaped) isotherm was observed, consistent with literature.^[^ ^29^ ^]^ Here, already adsorbed PFOA and PFOS seemed to strengthen hydrophobic–hydrophobic adsorption leading to co‐adsorption and/or agglomeration of GenX in the **UiO‐66** cages. However, in case of **UiO‐66‐(F)_4_**, the polymer–MOF hybrids, **UiO‐66‐OS** and **UiO‐66‐(F)_4_‐OS**, the GenX isotherms revealed negative slopes. The compositional modifications prevented co‐adsorption and/or agglomeration, likely because of steric hindrance. The adsorption of competing PFOA and PFOS resulted in the negative slope of the GenX isotherm. We modeled the competitive adsorption behavior based on the ideal adsorbed solution theory (IAST)^[^ ^30^ ^]^ using the individual (pseudo‐single‐solute) isotherm parameters for PFOA and PFOS (**Table** **2**), and then estimated the isotherm parameters for GenX (Figure S58, Supporting Information).

**Table 1:** Overview of PFAS adsorption experiments conducted in this study.

Experiment type	Initial PFAS concentration	PFAS species	MOF loading/batch volume	Equilibrium time	Type of PFAS matrix
Batch removal	100 µg L^−1^	PFOA, PFOS, GenX	10 mg/25 mL	24 h	Separate experiments
Adsorption kinetics	100 µg L^−1^	PFOA, PFOS, GenX	20 mg/50 mL	48 h	Mixture
Trace removal	2 µg L^−1^	11 PFAS	10 mg/25 mL	48 h	Mixture
Adsorption isotherm	100 µg L^−1^	PFOA, PFOS, GenX	2, 4, 6, 8, 10, 15 mg/25 mL	48 h	Mixture
Regeneration	5 µg L^−1^	PFOA, PFOS, GenX	2 mg on syringe filter/2 mL	1 drop per second filtration speed	Mixture

The adsorption performance is evaluated by the sorption coefficient *K*_d_ = *q*_e_/*C*_e_ at high residual concentration (i.e., *C*_e_ ≈ 50 µg L^−1^) for PFOA and PFOS (all *K*_d_ values alongside the corresponding isotherm data are available in Table 2, and further details are included in the Supporting Information dataset). With **UiO‐66**, we observe a decrease in sorption coefficient when applying the coating (e.g., from 7.93 × 10^3^ to 5.76 × 10^3^ L kg^−1^ for PFOA), while with **UiO‐66‐(F)_4_** we observe a significant increase in sorption coefficient (e.g., from 4.43 × 10^3^ to 1.20 × 10^4^ L kg^−1^ for PFOA). For comparison, the sorption coefficient for PFOA for three different GAC materials is in the range of 1 × 10^3.75^–1 × 10^5.11^ L kg^−1^, indicating comparable performance parameters.^[^ ^8^ ^]^ Figure S57 (Supporting Information) displays the experimental data and isotherm models for PFOA and PFOS for all four sorbents tested. The phase purity and isostructural nature of the sorbents following exposure to PFAS were confirmed by the consistent PXRD patterns observed for **UiO‐66‐X**, **UiO‐66‐X‐OS**, **UiO‐66‐X@PFAS,** and **UiO‐66‐X‐OS@PFAS** (where, PFAS = PFOA, PFOS, and GenX) (Figure 2F; Figures S3 and S4, Supporting Information). BET surface area analysis (Figure 2G; Figures S35–S50 and Table S4, Supporting Information) and pore size distribution analyses based on N_2_ adsorption isotherms (77 K; Figures S51–S54, Supporting Information) suggest that post PFAS exposure, the modest microporosity decreases in the pristine MOFs following the order: **UiO‐66‐(F)_4_@PFOS** < **UiO‐66‐(F)_4_@PFOA** < **UiO66@PFOS** < **UiO‐66@PFOA**, whereas the guest‐accessible surface areas increase in the OS‐coated derivatives following: **UiO‐66‐OS@PFOA** < **UiO‐66‐OS@PFOS** < **UiO‐66‐(F)_4_OS@PFOS < UiO‐66‐(F)_4_OS@PFOA**. The fact that sorbent microporosity was incommensurate with the sizes of the guest PFAS sorbates is further supported by these trends, sparking additional interest in critically interrogating the surface chemistry of these sorbents by spectroscopy, molecular modeling, and microscopy experiments.

**Table 2:** Summary of experimental results, comparing the acquired data through sorption coefficients (*K*_d_, at *C*_e_ ≈ 50 µg L^−1^), Langmuir and Freundlich adsorption isotherm models. Isotherm experiments were conducted in triplicate accompanied by triplicate blanks, with sorbent loadings of 2, 4, 6, 8, 10, and 15 mg in 25 mL, and a starting concentration of ≈100 µg L^−1^ for each PFAS present in the sample mixture.

Sorbate	PFOA							PFOS						
	Langmuir			Freundlich				Langmuir			Freundlich			
Sorbent	*R* ^2^	*K*_L_ [L µg^−1^]	*q*_max_ [µg g^−1^]	*R* ^2^	*K*_F_ [g_MOF_^−1^ L^−^ *^n^* µg_PFAS_^1−^ *^n^*]	*N*	*K*_d_ [L kg^−1^]	*R* ^2^	*K*_L_ [L µg^−1^]	*q*_max_ [µg g^−1^]	*R* ^2^	*K*_F_ [g_MOF_^−1^ L^−^ *^n^* µg_PFAS_^1−^ *^n^*]	*n*	*K*_d_ [L kg^−1^]
UiO‐66	0.99	0.027	805.3	0.97	53.83	0.547	7.93 × 10^3^	0.92	0.036	482.5	0.86	63.06	0.397	7.66 × 10^3^
UiO‐66‐(F)_4_	0.91	0.011	661.2	0.87	12.63	0.739	4.43 × 10^3^	0.98	0.026	913.3	0.98	42.65	0.651	1.08 × 10^4^
UiO‐66‐OS	0.80	0.023	438.7	0.84	25.35	0.569	5.76 × 10^3^	0.61	0.041	388.1	0.69	28.75	0.578	6.33 × 10^3^
UiO‐66‐(F)_4_‐OS	0.95	0.0035	3631.3	0.96	14.75	0.924	1.20 × 10^4^	0.97	0.015	2355	0.99	41.89	0.861	2.56 × 10^4^

**Figure 3:**
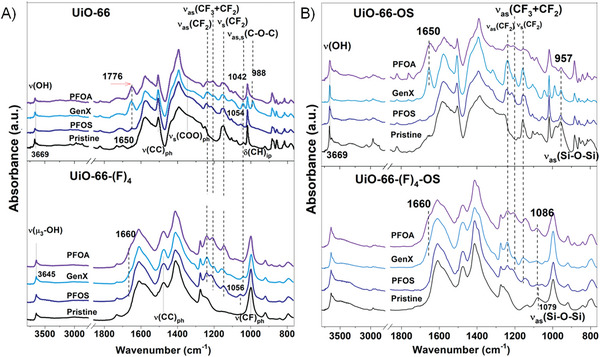
Infrared (IR) spectroscopy‐led insights into PFAS binding. IR spectra of pristine (bottom), PFOA, GenX, and A) PFOS‐loaded **UiO‐66** (top left), B) **UiO‐66‐OS** (top right), A) **UiO‐66‐(F)_4_** (bottom left), and B) **UiO‐66‐(F)_4_‐OS** (bottom right). The overwhelming signal of adsorbed water was removed by preannealing the samples up to 150 °C.

Before and after the PFAS (PFOA, PFOS, and GenX) capture, IR spectral signatures were recorded on **UiO‐66**, **UiO‐66‐(F)_4_**, **UiO‐66‐OS**, and **UiO‐66‐(F)_4_‐OS**. The loaded OS in **UiO‐66‐OS** is characterized by its strong *ν*_as_(CH_3_) and *v*_as_(Si─O─Si) bands, which are absent in the pristine **UiO‐66** spectrum (**Figure** **3**). Interestingly, the ν_as_(Si─O─Si) band appears at different positions in **UiO‐66‐(F)_4_** and **UiO‐66**, i.e., at 1079 and 957 cm^−1^, respectively. While the former is in the range of free OS (before loading) absorption frequency at ≈1100–1020 cm^−1^, the latter is shifted lower, i.e., redshifted.^[^ ^31^ ^]^ This suggests that OS could be absorbed on the external surface of **UiO‐66‐(F)_4_** but trapped inside **UiO‐66** nanopores, establishing stronger interactions with MOF structures, and thus shifting *ν*_as_(Si─O─Si) band to the lower frequency. The adsorbed PFAS molecules were found to exhibit features akin to those observed in the pristine **UiO‐66** and **UiO‐66‐(F)_4_** samples, e.g., the *ν*(C═O) band of PFOA and GenX appeared at 1650 cm^−1^ in **UiO‐66‐OS** versus 1660 cm^−1^ in **UiO‐66‐(F)_4_‐OS** (Figure S59 and Tables S5 and S6, Supporting Information). This indicates that the carboxylic acid groups A) primarily react with the OH groups of **UiO‐66** as evidenced by the decrease of the *ν*(OH) band intensity and B) interact with the exposed Zr^4+^ sites of **UiO‐66‐(F)_4_‐OS**. The lower adsorption, except for PFOS in **UiO‐66‐OS**, could be due to surface blocking by OS. It is worth noting that *ν*_as_(Si─O─Si) band in **UiO‐66‐OS** still remained in the same position at 957 cm^−1^; nevertheless, it appeared at slightly higher frequency (1086 cm^−1^) in **UiO‐66‐(F)_4_‐OS**, which pointed to the direct interaction between PFAS and OS in **UiO‐66‐(F)_4_** that accounted for their enhanced uptake.

**Figure 4:**
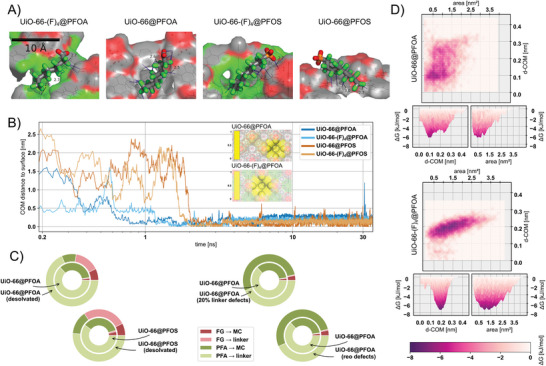
Examining the PFAS binding sites with molecular dynamics. A) Sample adsorption configurations at 25 ns for the two best‐performing MOF sorbents, taken from a single MD run. Marked up are interatomic distances of <0.3 nm. The three shortest distances are annotated in Å. B) Distance profile, recorded between the PFAS–center of mass (COM) and MOF surface (defined by the topmost atom) over the MD simulation time, corresponding to the systems shown as snapshots in panel (A); see Supporting Information for an average across multiple, different runs. The inset shows the contact ratio map for **UiO‐66@PFOA** and **UiO‐66‐(F)_4_@PFOA** systems, see Supporting Information for the corresponding visualizations of the **UiO‐66@PFOS** and **UiO‐66‐(F)_4_@PFOS**. Of particular importance is how the adsorption process with **UiO‐66** is not centered on a single cavity. C) Left: Comparison of relative contact counts (<0.4 nm) in **UiO‐66@PFOA**, as simulated with the solvent molecules and the adsorbed structures corresponding to a desolvated system. Right: Comparison of relative contact counts (<0.4 nm) in **UiO‐66@PFOA**, as simulated with the idealized system and more realistic defective structures. Contacts are differentiated by the type of subunits of the MOF (linker/metal cluster) and PFAS (backbone/functional group) system to which the contact‐pair atoms belong. Notably, removing solvent molecules, the contacts (i.e., as a proxy for interactions) between the functional group (FG) and the metal cluster (MC) elicit a marked increase in the desolvated system compared to the system as simulated with solvent molecules. Additionally, within the defective structures we see a marked increase in PFAS–MC contacts, compared to the idealized system, i.e., the PFAS–MC interactions seem to play a profound role in PFAS capture. D) Probability plots for **UiO‐66@PFOA** and **UiO‐66‐(F)_4_@PFOA**, in a specific (distance vs contact area) state, expressed in the units of free energy. The data were recorded across all simulation runs for the respective MOF@PFOA combinations in timesteps of 50 ns. Distances are recorded following the distances of the PFOA molecule's COM from the topmost MOF atoms, measured along the surface normal. Contact areas were determined with a modified solvent‐accessible surface area algorithm (see the Supporting Information for details). Both systems exhibit a single, extended free energy basin. Abbreviations: FG: functional group; MC: metal cluster.

To elucidate the atomistic behavior of PFAS adsorption, we conducted classical molecular dynamics (MD) simulations for the lead PFAS sorbents,^[^ ^32^ ^]^ **UiO‐66** and **UiO‐66‐(F)_4_**, solvated in water. Key outcome of these simulations is that the PFAS adsorption mechanism relies heavily upon the interspersed PFAS–MOF surface interactions in the hydrated system, standing against the common notion of PFAS transport occurring across microporous cavities leading to adsorption inside of those.^[^ ^33^ ^]^ For each tuple (**UiO‐66@PFAS** analog), we conducted five 35 ns NPT (isothermal–isobaric ensemble) simulations on an idealized surface model (**Figure** **4**A includes representative adsorption geometries; visualizations sampled across time as well as for the ─NX_2_ (X = H and O) analogs are included in Figures S60 and S67 in the Supporting Information). In general, we can discern two adsorption modes: the PFOA adsorbs relatively fast (within 1 ns), while the PFOS needs to find a preferential geometry with the FG oriented away from the *bdc*‐linker plane. Once the PFAS molecules get adsorbed, we could not observe any further detachment during the simulation runtime (Figure 4B). Contrary to literature reports, where the PFAS molecules fully enter the MOF cages,^[^ ^14a^ ^]^ it is crucial that we observe mostly surface‐level interactions with the center of mass (COM) of the specific PFAS sorbates never crossing the virtual plane encompassed by the MOF terminal cluster atoms. To quantify these observations, in **UiO‐66**, collation of sub 0.4 nm contacts between PFAS and MOF shows that the FG orients and interacts with the metal clusters (MCs) (Figures S63–S65, Supporting Information), while the perfluoroalkyl (PFA) (Scheme S2, Supporting Information) interacts with both the MC (in part for the orientation of the FG) and the *bdc*‐linker. For **UiO‐66‐(F)_4_**, interactions of FG with the MC are more pronounced whereas the PFA shows relatively stronger interactions with (F)_4_‐*bdc*. These observations can be spatially quantified across the full simulation runtime by analyzing per atom contact ratios (Figure 4B, inset), that is, the number of PFAS atom contacts per MOF atom per simulation frame. Here it becomes visually evident that PFAS adsorption onto **UiO‐66‐(F)_4_** can be associated with interactions occurring between the PFA groups and (F)_4_‐*bdc*, as well as between the FG and metal clusters. For **UiO‐66**, a more dispersed but similar interaction pattern can be found, and the plot indicated the PFAS molecules to be “hopping” across the **UiO‐66** surface while consistently interacting with MOF MCs.

Following the lead of Li et al.,^[^ ^14a^ ^]^ we present a free‐energy estimation profile, due to differences in the probabilities between different (COM–distance, contact area) bins (Figure 4D; Figure S66, Supporting Information). Altogether, PFAS adsorption in **UiO‐66‐(F)_4_** was found to exhibit a lower spread of MOF surface–PFAS distances in silico, while covering a range of contact areas up to 3.5 nm^2^. With the **UiO‐66** surface, we note the PFAS molecules entering deeper into the cavity, despite registering fewer interactions at high contact areas. As the interactions were found more concentrated around the PFA groups, no qualitative difference between different PFAS molecules could be found in this analysis. With this positive evaluation of our model system showing consistent adsorption behavior, we selected the best‐performing analog (**UiO‐66**) for further studies. Figure 4C shows the relative distribution of sub 0.4 nm contacts comparing the desolvated system to its solvated counterpart—this shows a marked increase in functional group–MC interactions supporting the detection of bond formation indicated by IR experiments. In addition, the introduction of defects comprising 20% of linkers randomly removed or a missing cluster (**reo**‐like) resulted in a marked increase of PFAS–MC interactions (Figure 4C).^[^ ^34^ ^]^ This is consistent with earlier observations made with the trace PFAS removal experiments (Figure 2D,E).

Overall, these simulation results qualitatively support that the highly efficient adsorption behavior of the **UiO‐66** platform of sorbents for PFAS is related to MOF‐specific FG and PFA–MC interactions, which might lead to a stronger and more specific adsorption than the competing PFA–*bdc* interactions found in other systems.^[^ ^15^, ^35^ ^]^ An increase in defects in our systems, a situation likely to occur in our PFAS exposed samples (at the post‐immersion stage), results in a marked increase in these PFAS–MC interactions, further strengthening the experimental observations from IR spectroscopy. Strong FG–MC interactions that we observe here are in agreement with the most recent literature.^[^ ^36^ ^]^

**Figure 5:**
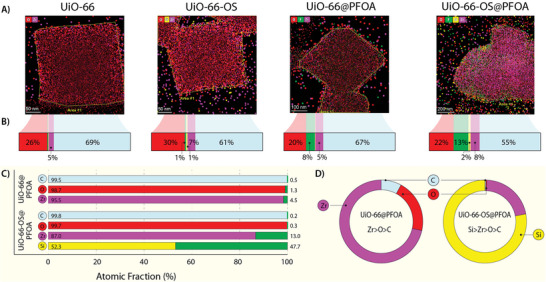
HAADF‐STEM imaging‐based PFAS–MOF interactions. A) EDX elemental maps for single particles collected from the four studied sorbents, **UiO‐66**, **UiO‐66‐OS**, **UiO‐66@PFOA**, and **UiO‐66‐OS@PFOA** (carbon signals were removed to reduce image saturation and improve clarity). B) The corresponding EDX elemental compositions. C) Relationships between the atomic fraction % of the identified elements compared to that of F in **UiO‐66** and **UiO‐66‐OS** after incubation with PFOA, that is, in **UiO‐66@PFOA** and **UiO‐66‐OS@PFOA**. D) Two doughnut charts representing the F retention with respect to the other elements present in the sorbents after PFOA incubation, i.e., in **UiO‐66@PFOA** and **Ui‐O‐66‐OS@PFOA**. Color codes—O: red; Zr: pink; Si: yellow; F: green; and C: blue).

Utilizing high‐angle annular dark field‐scanning transmission electron microscopy (HAADF‐STEM) imaging technique, energy‐dispersive X‐ray (EDX) spectroscopy was conducted for **UiO‐66**, **UiO‐66‐OS**, and the corresponding pair of sorbents incubated with PFOA, i.e., **UiO‐66@PFOA** and **UiO‐66‐OS@PFOA**, respectively (**Figure** **5**A). To establish the effectiveness of each sample variation at capturing PFOA, elemental analysis was performed where the key indicator for PFOA retention is the quantitative detection of F (stemming from the sorbate PFOA) (Figure S68, Supporting Information). From the EDX spectroscopic data, the atomic fraction compositions were quantified and represented as a bar chart in Figure 5B to better understand the elemental composition and comparison between the samples (Figure 5B). Si signals in **UiO‐66** and **UiO‐66@PFOA** and the F signals in **UiO‐66** and **UiO‐66‐OS** were found to be negligible, as expected (Figure 5B). For **UiO‐66‐OS@PFOA**, the atomic fraction of F (at%) was found to be higher (13%) than that found in **UiO‐66@PFOA** (8%), suggesting the likelihood of **UiO‐66‐OS** to be a more effective sorbent to capture PFOA versus **UiO‐66**. The ratios of F with respect to C, O, Zr, and Si were monitored to establish the dependence of F on each of the elemental components (Figure 5C). For **UiO‐66**, a notable dependence on Zr was noticed; however, this was three times more pronounced in **UiO‐66‐OS**. F was found to be significantly dependent on the Si component of **UiO‐66‐OS**, thus contributing to its PFOA capture performance. The propensity of F's (stemming from PFOA) relationship to the other elemental components was Zr > O > C for **UiO‐66**, and Si > Zr > O > C for **UiO‐66‐OS** (Figure 5D).

Enriched with the learning on the nature of interactions between PFAS and the studied best‐performing materials, we designed a regeneration study which allows us to perform five consecutive adsorption and regeneration cycles with each adsorbent at microscale by immobilizing them on the frit of a polypropylene syringe filter, as described in the “Experimental Details: Microscale Column Regeneration Experiments” section in the Supporting Information. Prior to conducting a regeneration study with five cycles of adsorption and regeneration, we first determined which regeneration solvent to use. As polar solvents are known to effectively disrupt both electrostatic and hydrophobic bonds between the PFAS and sorbent molecules, whereas low‐polarity solvents efficiently regenerate hydrophobic adsorbents,^[^ ^15^, ^28^, ^37^ ^]^ we narrowed down the solvent selection to methanol, methanol + 1% NaCl (70–30 v/v), and acetone. Acetone was selected as a potentially high‐performing solvent for PFAS regeneration from sorbents that adsorb PFAS predominantly via hydrophobic‐hydrophobic interactions, consistent with our earlier observations in **UiO‐66‐(F)_4_** and the OS‐coated derivatives, **UiO‐66‐OS** and **UiO‐66‐(F)_4_‐OS**. We observed MeOH‐NaCl to outperform the other two solvents marginally, as presented in Figure S69 (Supporting Information).

**Figure 6:**
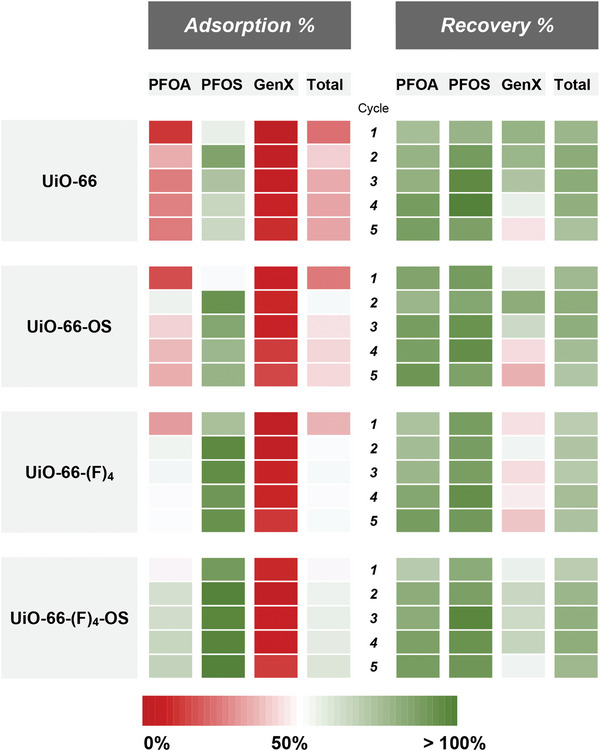
PFAS capture and recycling performance. The comparison of adsorption and regeneration efficiency over five cycles for PFOA, PFOS, and GenX, as well as the total PFAS removal and recovery. Adsorption represents the amount of PFAS removed compared to the blank control sample, while regeneration represents the amount of the adsorbed PFAS that was subsequently recovered (explained with Equations (S1)–(S4) in the Supporting Information). Adsorption and regeneration performance of materials were tested in duplicate, while the blank tests (using syringe filters without adsorbent loading) were conducted identically but in triplicate and those concentrations used as the starting concentrations. A slight improvement in both adsorption and regeneration performance can be observed with **UiO‐66‐(F)_4_‐OS** over the course of five cycles.

Upon selecting MeOH‐NaCl as our regeneration solvent (Figure S69, Supporting Information), we proceeded to conduct a 5‐cycle regeneration study for all four sorbents in duplicate; we alternately passed and captured the PFAS solution (adsorption) and the regeneration solvent (recovery) through the syringe filter. Syringe filters without any adsorbent material were used in an identical fashion to the prepared MOF‐carrying columns, to obtain blank values fit for use as C_0_ in our calculations. While polypropylene syringes that were used throughout this study performed well in the sense that not much PFAS were adsorbed to them, some non‐negligible adsorption of PFAS occurred on the syringe filter frit itself. Therefore, blanks were necessary, and were conducted in triplicate, the average of which was used for all calculations as C_0_. Further details on the methodology, the rationale for the calculations, and the formulae can be found in the “Experimental Details: Microscale Column Regeneration Experiments” section of the Supporting Information. The adsorption and regeneration performance over the course of five cycles, both for individual PFAS and their sum, are presented in **Figure** **6**.

Experimentally, we observed modest adsorption performance for PFOA, and only minor adsorption of GenX, while PFOS adsorbed well on all four MOFs, with exceptionally high adsorption performance of **UiO‐66‐(F)_4_‐OS** over all five cycles. Further, PFOS desorbed most readily from all four sorbents, with >90% of the adsorbed PFOS accounted for in the regeneration solvent. PFOA regenerated well too, with >80% of the adsorbed PFOA accounted for in the regeneration solvent in most cases. GenX displayed decent regeneration capabilities as well, but considering the general trend of poor GenX adsorption performance compared to PFOA and PFOS, its negative impact on the total adsorption and regeneration performance was notable. While looking at the total adsorption and regeneration performance of the four materials, we observed stabilization in adsorption performance over the five cycles for **UiO‐66**, **UiO‐66‐OS**, and **UiO‐66‐(F)_4_**, while **UiO‐66‐(F)_4_‐OS** displays a steady increase in adsorption performance as the cycles progress. In the case of regeneration, **UiO‐66** and **UiO‐66‐OS** register a decreasing trend in performance, whereas **UiO‐66‐(F)_4_** and **UiO‐66‐(F)_4_‐OS** exhibit stable trends in regeneration, with **UiO‐66‐(F)_4_‐OS** demonstrating the highest regeneration performance overall. A side‐by‐side comparison of the adsorption and regeneration data in the form of bar graphs presented with standard deviations are available in Figures S70 and S71 (Supporting Information).

## 4 Conclusion

We have thus designed and systematically studied two families of regenerable (and stable) MOFs and MOF–organosilicone composite physisorbents; one from each family offers excellent ppb‐level PFAS removal at lab scale. IR spectroscopy, HAADF‐STEM imaging‐based EDX analysis, and MD simulations—the findings from all three experiments, consistently revealed the profound impact of OS coating and the importance of the availability of Zr(IV) sites in inducing efficient PFAS capture. Simply put, surface functionalization reveals clear improvement in the PFAS removal performance as compared to the traditional approach of harnessing pores for immobilizing sorbate molecules. To set new PFAS removal benchmarks and to overcome the oft‐encountered limitations of the state‐of‐the‐art PFAS adsorbents (i.e., activated carbons and IXR), future research efforts should likely focus upon the development and study of the next generation of MOFs and polymer–MOF hybrids,^[^ ^38^ ^]^ when rightly surface‐functionalized (vide the proposed sorbent design paradigm in Figure S72 in the Supporting Information).^[^ ^39^ ^]^ Albeit the narrow pores’ unsuitability to fit the PFAS sorbate molecules inside, this study offers insights into which MOF functionalization routes elicit optimal surface properties most suitable for PFAS removal. Relying largely upon liquid chromatography coupled with mass spectrometry, high ppb‐level PFAS removal efficiencies have been realized in **UiO‐66** and **UiO‐66‐(F)_4_‐OS**. Perhaps most importantly, our study emphasizes the nonessential nature of guest‐accessible porosity in enabling water decontamination from persistent and mobile organic compounds, including but not limited to PFAS. In essence, adjusting microporosity and/or controlling pore size and environment seems to be less crucial than adjusting the external particle surface and refining the interface for the hydrophobic coating. Leaning into the future, a defect‐rich MOF nanoparticle exposing Zr(IV) sites hosted with organosilicone coating, offering the ideal combination of hydrophobicity, stability, and electrostatics might well be the solution to address the challenge of PFAS predicament.

## Conflict of Interest

The authors declare no conflict of interest.

## Supporting information



Supporting Information

## Data Availability

The data that support the findings of this study are publicly available in the open repository developed under the European OpenAIRE program, DOI: 10.5281/zenodo.12634526 (weblink: https://zenodo.org/records/12634526).
