# Supplementary material for: Trace Adsorptive Removal of PFAS from Water by Optimizing the UiO‐66 MOF Interface
Source: Adv Mater. 2024 Nov 21;37(6):2413120. doi: 10.1002/adma.202413120 (PMC11817902; doi:10.1002/adma.202413120)
Supplement: Supplementary file 1 — Supporting Information [file ADMA-37-2413120-s001.pdf]

ADVANCED MATERIALS

Supporting Information

for *Adv. Mater.*, DOI 10.1002/adma.202413120

Trace Adsorptive Removal of PFAS from Water by Optimizing the UiO-66 MOF Interface

Nebojša Ilić, Kui Tan, Felix Mayr, Shujin Hou, Benedikt M. Aumeier, Eder Moisés Cedeño Morales, Uwe Hübner, Jennifer Cookman, Andreas Schneemann, Alessio Gagliardi, Jörg E. Drewes, Roland A. Fischer* and Soumya Mukherjee**

Supporting Information for

Trace Adsorptive Removal of PFAS From Water by Optimizing the UiO-66 MOF Interface

Nebojša Ilić,[1] Kui Tan,[2] Felix Mayr,[3] Shujin Hou,[4] Benedikt M. Aumeier,[1] Eder Moisés Cedeño Morales,[3] Uwe Hübner,[1,5] Jennifer Cookman,[6] Andreas Schneeman,[7] Alessio Gagliardi,[3] Jörg E. Drewes,[1] Roland A. Fischer,*[4] and Soumya Mukherjee*[4,6]*

[1]Chair of Urban Water Systems Engineering, Technical University of Munich, Am Coulombwall 3, 85748 Garching, Germany.

[2]Department of Chemistry, University of North Texas, 1155 Union Cir, Denton, TX, 76203, USA.

[3]Chair of Simulation of Nanosystems for Energy Conversion, Department of Electrical Engineering, TUM School of Computation, Information and Technology, Technical University of Munich, Hans-Piloty-Straße 1, 85748 Garching, Germany.

[4]Chair of Inorganic and Metal-Organic Chemistry, Catalysis Research Center, School of Natural Sciences, Technical University of Munich, 85748 Garching, Germany.

[5]Xylem Services GmbH, Boschstraße 4-14, 32051, Herford, Germany.

[6]Bernal Institute, Department of Chemical Sciences, University of Limerick, Limerick V94 T9PX, Ireland.

[7]Inorganic Chemistry I, Technische Universität Dresden, Bergstraße 66, 01069 Dresden, Germany.

Corresponding authors contact: jdrewes@tum.de, roland.fischer@tum.de, and soumya.mukherjee@ul.ie

Materials and methods.

Starting materials, reagents, and solvents were purchased from commercial sources (Sigma-Aldrich, Acros Organics B.V.B.A., AK Scientific Inc. and TCI chemicals Europe N.V.) and used without further purification. A full list of PFAS chemicals used in this study is available in **Table S1**. Dowsil™ / Dow Corning 1-2577 conformal coating was procured from VWR Deutschland and was used as the organosilicone (a.k.a. DC 1-2577).

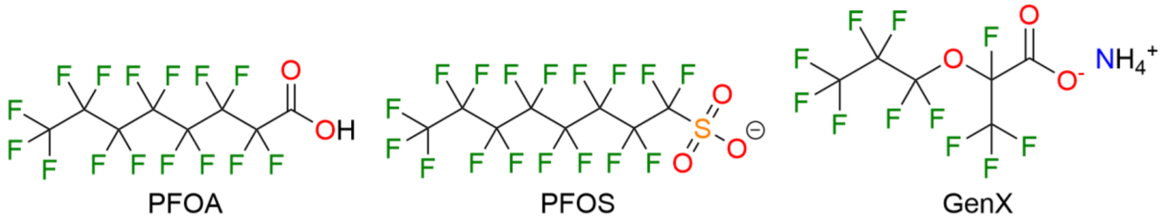

Scheme S1. Three PFAS chemicals examined in this study as the primary focus (PFOA: perfluorooctanoic acid; PFOS: perfluorooctanesulfonate; GenX: ammonium salt of hexafluoropropylene oxide dimer acid.

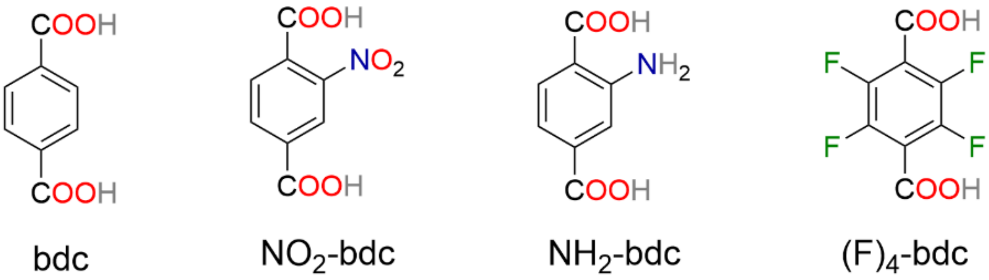

Scheme S2. Linker ligands used in this study to prepare variants of **UiO-66-X** (X = H, $(F)_4$, NO_2, NH_2).

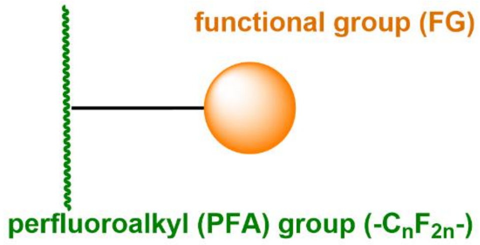

Scheme S3. Two components constituting the PFAS molecules: carboxylates/sulphonates are regarded as the functional group (FG), whereas the hydrophobic tail is known as the perfluoroalkyl group (PFA).

Initial high concentration stocks were made in methanol for long-term storage, and used to prepare the lower concentration stock solutions used for experiments.

Experimental details.

X-ray diffraction measurement. Diffractograms were recorded using Bragg-Bentano geometry with a silicon wafer plate on a Rigaku Benchtop MiniFlex 600-C. X-ray Cu Kα radiation (λ = 1.5406 Å) with a voltage of 40 kV and current of 15 mA was used. The data was collected from 5°–30° (2θ) with a step-size of 0.01° and a scan time of 30 seconds per step. Crude data were analysed using the X'Pert HighScore Plus™ software V 4.1 (PANalytical, The Netherlands).

Thermogravimetric analysis (TGA). Thermogravimetric analysis coupled with differential scanning calorimetry (TGA-DSC) was conducted on a Netzsch TG-DSC STA 449 F5 in a temperature range from 25 °C to 800 °C with a heating rate of 10 K min^{-1} (min = minute) under argon flow (flow rate: 20 mL min^{-1}). It

should be noted that the sample was briefly (few seconds) exposed to air before the measurement, when the aluminium oxide pan was transferred from the argon filled transport vial to the sample holder stage.

Scanning electron microscopy (SEM). SEM images were obtained using a NVision40 (Carl Zeiss AG) field emission scanning electron microscope, with an accelerating voltage of 5 to 7.5 kV, and a working distance range of 7.2 to 10 mm.

Nitrogen adsorption isotherm measurements. Adsorption measurements with N_2 (> 99.999 vol%) at 77 K were carried out on a Micromeritics 3Flex surface area and pore size analyser, which uses a manometric method to determine the amount adsorbed at an equilibrated gas pressure. Activated samples were transferred under dry argon atmosphere into preweighed sample tubes and capped with Micromeritics CheckSeals. Samples were subsequently activated again at 120 °C for 5 h under dynamic vacuum of ca. $1x10^{-3}$ mbar using a SmartVacPrep by Micromeritics Instrument Corp. to ensure absence of unwanted adsorbates and identical pre-measurement states of all samples. The absorbent mass was then recorded, generally in the range of 70 – 85 mg. To facilitate proper degassing in context of the highly diffusion hindering pore environment, prior to each measurement samples were evacuated in situ at 80 °C for at least 20 h under dynamic vacuum of ca. $1x10^{-5}$ mbar. Free space of the sample tube was determined after measuring each adsorption isotherm using helium (> 99.999 vol%). A liquid nitrogen bath was used for measurements at 77 K. BETSI.exe was used to determine the BET surface areas.[1] The corresponding BETSI analysis (generated by BETSI.exe) for all studied sorbents are included as Figures S13-S24; S39-S50. The corresponding pore size distribution profiles for each of the adsorption isotherms are included as Figures S25-S28; S51-S54 (geometry: slit; model: N_2 at 77 K on Carbon Slit pores). These were obtained by fitting the NLDFT model to the respective 77 K N_2 adsorption branches.

Water vapour adsorption isotherm measurements

Water vapour adsorption measurements were conducted using a Belsorp-Max porosimeter (Microtrac Bel) equipped with a cryostat to maintain the temperature of the sample cell at 298 K during the measurements. The measurements were conducted with deionised water, which was degassed by three freeze-pump-thaw cycles via the solvent degassing-routine of the instrument. For each measurement around 50 mg of sample were pre-treated by heating to 120 °C in dynamic vacuum overnight (10^{-3} mbar).

Static contact angle measurements

Static water contact angles were recorded on pressed sample pellets (after subjecting solid samples to mechanical pressure of *ca*. 1.5 ton press) with a Krüss EasyDrop Kys DAS 10 MK2 contact angle meter, followed by Krüss Advance software-based analysis (Young-Laplace Fit using Sessile Drop Analysis method). The analysis is based on the fitting of the Young–Laplace equation to the droplet image data (droplet shape analysis), by using 10 μL of distilled water, with a precision of ± 0.5°.

Infrared spectroscopy (IR).

IR measurements were performed on a Nicolet 6700 FTIR spectrometer using a liquid N_2-cooled mercury cadmium telluride (MCT-A) detector. The spectrometer is equipped with a vacuum cell that is placed in the main compartment with the sample at the focal point of the infrared beam. Each of the samples (≈ 5 mg) was gently pressed into a KBr pellet, and placed into a cell that was connected to a vacuum line for evacuation of trapped water. The samples were activated by heating up to 150 ºC, and then cooled back to room temperature for IR measurements.

HAADF-STEM imaging based EDX studies.

The four samples analysed by scanning transmission electron (STEM) and energy dispersive X-ray spectroscopy (EDX) were deposited each on a lacey carbon Cu TEM grid for 1 h and then the excess tapped off. The grids were then stored in membrane boxes. STEM imaging and EDX spectroscopic analysis were

conducted on a Thermo Fisher Scientific Titan Themis Cubed operating at 300 kV. Initially, the microscope was aligned according to user alignments and further tuned with the probe corrector using a gold standard calibration grid. Once complete, each sample was placed in a double tilt holder for STEM imaging and EDX analysis.

Collection of the EDX spectroscopic data and subsequent analysis was conducted using the software Velox (Thermo Fisher Scientific). Analysis of the EDX experiments were conducted on an offline version of Velox where background correction was completed and the appropriate particles were outlined to exclude signals from other features, e.g., carbon substrate, where possible. Velox quantifies the spectra in each pixel and performs a least square (empirical) fit, most appropriate for sparse datasets. Atomic fraction % and associated fir error was gathered from these data to elucidate the fraction of each element in the sample.

Significant signals were recorded for C, O, Zr for all samples, and F for the PFOA incubated samples, and Si for the organosilicone treated samples.

When conducting EDX there were some cautions worth considering, for example in the samples that were not incubated with PFOA and hence the absence of a F signal is expected, there is a minor peak identified at approximately 700 eV that could be interpreted as a residual signal of F. However, this signal is due to the iron components of the electromagnetic lenses of the TEM where iron is expected at 704.5 eV. Additionally, a copper signal is experienced at 930 eV which can be attributed to the copper TEM grid where the sample is placed. This signal is identified in EDX spectra that were acquired close in proximity to the copper grid bar and is observed in the green line of **PFOA@UiO-66**. This is also the case for samples that were not treated with organosilicone where a minor peak is identified at approximately 2000 eV where silicon is expected. Through further investigation this minor signal was attributed to the Zr Lα2 signal expected at 2039.9 eV.

LC-MS quantification of PFAS.

PFAS concentrations in all the collected samples were quantified with an LC/MS-MS system (Agilent 1260 Infinity, ABSciex Qtrap 5500). The analytical method for this system was developed with regards to the German standard method DIN 38407-42, supported by the US Environmental Protection Agency (U.S. EPA) Method 537.1. The LC system was connected without the mixing chamber and dampener for lower retention times. A PFAS delay column (Agilent ZORBAX Eclipse plus; C18 95 Å, 3.5 μm, 4.6 mm × 30 mm) was introduced before the injection valve to delay potential PFAS signals originating from the instrument or solvents. PFAS separation was accomplished with a Waters XSelect HSS T3 Column (F100 Å, 3.5 μm, 2.1 mm × 100 mm) with the column oven set to 30 °C. The mass spectrometer was operated in negative electrospray ionisation mode using multiple reaction monitoring (MRM) to measure all PFAS simultaneously. The sample injection volume was 50 μL, and the flowrate 400 $\mu L\ min^{-1}$. The analytes were eluted with solvent A (ultrapure water + 0.1% formic acid) and solvent B (methanol + 0.1% formic acid). Ultrapure water and methanol blanks were included in the sequence before and after the calibration standards, as well as after every 5 sample injections, and at the end of the measurement run.

PFAS of primary interest for the study (GenX, PFOA and PFOS) were purchased as analytical grade methanol solutions, while the other 7 PFAS measured were purchased as reagent grade chemicals and 1 mL high concentration methanol stock solutions were prepared for each PFAS from them. ^{13}C mass-labelled internal standards for GenX, PFOA and PFOS were used for spiking samples and standards throughout the study for control.

Table S2 contains m/z ratios for fragments scanned for each PFAS in the method. PFAS were always quantified using calibration curves generated from one fragment, while the second fragment was used for validation. Generated calibration curves displayed good fits (R^2 > 0.99). The LC/MS-MS system had an effective detection range between 5 and 5,000 ng L^{-1}. MultiQuant 3.0.3 software was used to analyse and curate the acquired data.

Synthesis.

UiO-66-X (X = H, NH_2, NO_2) derivatives were synthesised following the defect-controlled synthesis method reported by DeStefano *et al.*,[2] while **UiO-66-(F)$_4$** was prepared following the original literature from Hu *et*

al.[3] We reproduce the four **UiO-66-X-PDMS** (X = H, NH_2, NO_2, $(F)_4$) derivatives following the post-synthetic polydimethylsiloxane (PDMS) treatment procedure reported by Zhang *et al*. (see detailed characterisation: Figures S1, S6, S8-S12, S14, S17, S20 and S23 in the Supporting Information).[4] The four **UiO-66-X-OS** (X = H, NH_2, NO_2, $(F)_4$) derivatives were synthesised following a slightly revised procedure than the one adopted by Qian *et al*. to post-synthetically modify different MOFs, such as, **NH_2-MIL-125(Ti)**, **ZIF-67**, and **HKUST-1** (see below and the detailed characterisation. Figures S2, S7, S8-S11, S15, S18, S21 and S24, S32 in the Supporting Information).[5]

Synthesis of UiO-66-X-OS:

80 mg organosilicone, *i.e.*, DC-1-2577 was dissolved in 12 mL heptane, to which 1 g of activated **UiO-66-X** powdered crystallites were charged. The reaction mixture was ultrasonicated for 0.5 h, following which it was dried under Schlenk vacuum at 303 K for 12 h.

Computational methods.

Force Fields and Partial Charges: In line with previous work for similar structures,[6] the molecular dynamics (MD) simulation was created around a fixed MOF structure, inserted into a periodically repeating cell. The basic, computation-ready structure of **UiO-66** was taken from literature,[7] and the corresponding analogues of **UiO-66-(F)$_4$**, **UiO-66-NH_2** and **UiO-66-NO_2** were derived based on that structure. Subsequently, their unit cells were relaxed to a force-difference threshold below 0.05 eV $Å^{-1}$. Lennard-Jones force-field parameters for the MOF atoms were taken from the UFF-force-field,[8] while the water solvent and the PFAS molecule were both parametrised using a fully bonded GAFF and OPLS-AA-parametrisation. In both cases, the TTIP3-model was used for water and parameters for describing the PFA part, which formed a characteristic helix-like structure (as indicated in previous works[6a, 9]).[10] Comparative studies showed no difference between the OPLS-AA and the GAFF parametrisation, the latter being chosen for production runs. For modelling the Coulomb interactions, the PME-method was used with a cut-off of 1.4 nm. Partial charges for the MOF structure were calculated using the RESP-protocol and the GAPW-approach in CP2K.[11] They were found to be in quantitative agreement with previous literature.[7]

Protocol: Basic model systems were created using a cubic-cell of the **UiO-66** analogues, which was arranged in a 3 × 3 × 2 slab, with periodicity in the *xy*-plane. The thickness of the resulting slab is approximately 4 nm. A simulation cell was created by padding the cell along the *z*-axis with approximately 8 nm of vacuum, which was then filled with water molecules. A well-defined surface was created by ensuring that the *xy*-planes consisted of full metal-cluster for the MOFs. Any non-bonded MC attachment sites at the interface were saturated with a single C atom. Finally, any residual charges of the resulting setup were equilibrated with Cl^- ions. The MD simulation protocol followed the standard of running a NVT and subsequent NPT equilibration simulation before production runs. Water loading was equilibrated with a 1 ns NVT run, followed by a 4 ns NPT run. The resulting water loading per **UiO-66** cell was found in agreement with a previous work reporting water loading.[12] A modified Berendsen thermostat was used to set a temperature of 300 K, and Berendsen coupling was used for pressure coupling at 1 bar. For the final production runs, the PFAS ions were inserted in a random orientation of about 2 nm along the z-axis from the top MOF atoms, approximately at the cell centre. While the ion was restrained, 1 ns NVT and NPT equilibrations were run as preparation steps, following which the 35 ns NPT-production runs were created and analysed. To approximate the behaviour of the system when desolvated, we took 75 snapshots of the PFAS-adsorbed states and relaxed the PFAS in the absence of water. Defective systems were created by repeating the cubic cell to a 2 × 1 × 3 slab, then removing 20% of the linkers and capping the connection points with COOH [13]. For this system partial charges were calculated using the RESP formalism and the system was replicated to a 4 × 3 × 3 slab of the original cell for MD. In a similar fashion, a metal cluster was removed from the surface of a 2 × 2 × 3 slab, with the slab then replicated for a 4 × 4 × 3-structure - forming a **reo**-phase defect. The appearance of such defects in **UiO-66** has been extensively documented both in theoretical,[12-13] and experimental work.[14]

Analysis: To quantify the attachment processes, extensive analysis of the PFAS-MOF contact interactions was performed. A contact was counted when the distance between any PFAS atom and a MOF atom was found to be smaller than 0.4 nm. These contacts were collated according to the classification of participating atoms (MOF-MC, MOF-linker)–(PFA-FG, PFA-(CF)-chain) [Supporting Information, Figures S63-S65]. Contacts were evaluated every 5 ps across 7000 frames per production run. Any contacts arising from the backbone carbon chain of the PFAS were omitted from the analysis as those atoms are buried inside of the molecular surface composed of fluorine vdW-shells. While this kind of analysis cannot provide quantitative insight into bonding behaviour (especially across systems of varying size), it provides a good overview of where the interactions happen, acting as proxy which allows to deduct possible bonding patterns. Furthermore, a contact area was calculated using a modified SASA-like algorithm implemented in Python using UFF-vdW-radii.[15] The contact ratio maps (manuscript, Figure 4D; Supporting Information, Figure S61) were derived from this analysis, counting a MOF atom as contacted if there was a shared interfacial area, and then normalizing with the number of frames. A basic analysis of the relationship between PFAS-COM distances to the surface and the associated contact area was performed. The probability of the system being in a (COM-distance, contact area)-state was visualised in units of the free energy using the transformation $dG = RT \ln(P/P_0)$, where P is the probability of a specific state, and P_0 is the base state with respect to which the free energy difference is determined.

Ideal adsorbed solution theory (IAST) calculations: To model the competitive adsorption behaviour, a numerical solution of the IAST was implemented in Python. The IAST was originally developed by Myers and Prausnitz,[16] and later transferred to solve problems of dilute solutions.[17] Here, the individual Freundlich isotherm parameters for PFOA and PFOS were taken from the isotherm models (Table 1, manuscript) assuming single-solute behaviour. Units were converted to molarities and no further corrections were performed. The IAST isotherm parameters for GenX were finally estimated to obtain a good fit with experimental data. The results are depicted in Figure S58.

PFAS adsorption experiments.

In all cases, the material was subjected to hydration before PFAS was spiked and the experiment started. 90% of the total batch volume was first added to a vial, before adding the material, and sonicating it in an ultrasound bath for 15 minutes. The materials were then left in ultrapure water overnight, and in the morning the remaining 10% of total batch volume was spiked as a PFAS stock with a 10x concentration of the planned initial concentration. This indicated the beginning of the experiment. All experiments were conducted at an adjusted initial pH of 6.5. In order to eliminate influence from external factors and to focus on the adsorption performance changes originating from compositional fine-tuning, all adsorption experiments were conducted using synthetic PFAS solutions prepared with ultrapure water. The sequence of addition played an important role in experiment setup, where the fine powder was added after water to prevent it from locking into the bottom of the vial due to the formation of a hydrophobic barrier. It was then sonicated and left to sit overnight before the PFAS was spiked. Throughout the experiments, the powder appears to have maintained good overall contact with the water and revealed excellent reproducibility between replicates.
To terminate the adsorption process immediately after sampling, samples taken from the batch vials were filtered through a polypropylene (PP) syringe filter into Eppendorf vials for storage. All samples were kept refrigerated until analysed. After the experiment, the MOFs were filtered out, dried, and then stored in a desiccator for future desorption and structural integrity tests. All laboratory equipment was cleaned using an ultrasound bath and by rinsing multiple times with ultrapure water and methanol.

Preliminary batch PFAS adsorption performance screening.

The initial screening of PFAS capture performance was based on batch adsorption tests. The batch removal experiment was conducted in 4-5 repetitions for each material due to the explorative nature of the experiment, where every material was tested for its ability to remove PFOA, PFOS and GenX individually. Observations

from these preliminary experiments were used to evaluate which materials are worth examining further for their PFAS removal capabilities.

Batch adsorption experiments were set up in triplicate using 30 mL PP bottles containing 10 mg of adsorbent in 25 mL of synthetic PFAS solution, and an initial PFAS concentration of 100 μg L^{-1}. After 24 h of equilibration in a water bath shaker, the samples were taken out, shaken intensely by hand, and then 2 × 1 mL are sampled with a pipette directly into a syringe with its piston pulled out and fitted with a PP syringe filter (PP frit, 13 mm diameter, 0.22 μm pore size). To avoid any material passing into the filtered samples, the first 5 drops of filtrate were discarded, as it was observed that first 2-3 drops resulted in some turbidity.

Adsorption kinetics.

After determining preliminary PFAS adsorption performance, selected high-performing materials were examined for their adsorption kinetics, to determine the equilibration time necessary for trace removal and isotherms. As these experiments required multiple samples from the same batch over time, we kept the solid/liquid ratio the same but doubled the batch volume of kinetics experiments (20 mg of material in 50 mL batch volume). Consequently, the protocol was then adjusted to adding 20 mg to 45 mL of ultrapure water, sonicating, and then spiking with 5 mL of PFAS stock, marking the start of the kinetics experiment. The initial concentration (t = 0 s) for the kinetics experiments was determined by filtering 2 mL of separately prepared diluted PFAS stock (200 μL in 1800 μL) through a PP syringe filter (PP frit, 13 mm diameter, 0.22 μm pore size) in an identical manner to the samples to account for any adsorption of PFAS to the filter. Adsorption kinetics, and all further adsorption related experiments were done with a PFAS stock mixture, rather than individually, both due to practicality and potential competition in adsorption behaviour between the different PFAS. Samples were taken after 3, 6, 9, 12, 15, 20, 30, 60 minutes, and after 24 h and 48 h.

Trace level batch adsorption of eleven PFAS chemicals (together).

Removal performances for all materials were tested for 11 PFAS in mixture at an individual concentration of 2 μg L^{-1}. Experiments were conducted identically to the batch removal experiments, except the spiking solution contained a mixture of 11 PFAS with an individual concentration of 20 μg L^{-1}. The full library of pristine and OS-coated MOFs was tested to examine whether shorter chain PFAS would display different adsorption performance trends when compared to the three main PFAS tested in batch removal experiments.

Isotherm adsorption experiments.

To evaluate the adsorption performance and changes due to coating in more detail, isotherm experiments were set up for the best-performing **UiO-66**, **UiO-66-(F)$_4$**, and their OS composite analogues, **UiO-66-OS** and **UiO-66-(F)$_4$-OS.** A mixture of PFOA, PFOS and GenX is spiked in the same manner as with batch experiments, into triplicate samples with different adsorbent loadings (2, 4, 6, 8, 10, and 15 mg). The equilibrium concentrations are then observed after the samples were taken and filtered after 48 h, and Langmuir and Freundlich isotherm models are considered for their goodness of fit.

Microscale column regeneration experiments.

To evaluate the column application potential of the sorbents on a micro scale, regeneration experiments were conducted by immobilizing the MOFs / polymer-MOF hybrids on syringe filters, and applying these as micro-scale adsorption columns. A screening analysis was conducted to determine the best approach to preparing a suspension of the MOFs. Literature and previous practical experience resulted in selecting tween-20 (often used in different laboratories for suspension creation, 92 μL in 100 mL ultrapure water), dishwashing soap (DWS, per recommendation of tween-20 manufacturer website, 0.1 g in 100 mL ultrapure water), absolute ethanol (as recommended in literature[18]), and absolute isopropanol (acquired research experience), as the suspension mediums. We weighed 2 mg of each sorbent and added it to an Eppendorf vial, after which 2 mL of the suspension medium was added to the vial. The samples were then sonicated for 15 minutes, and pictures taken directly after sonication, and after 1 h. The most stable suspension for all materials tested was with

ethanol, so we proceeded to prepare all the corresponding MOF suspensions using ethanol (1 mg mL^{-1}). The suspension was then sonicated, and 2 mL was passed through a PP syringe filter (PP frit, 13 mm diameter, 0.22 μm pore size), the filtrate was captured and was passed through again to recover any MOF particles that passed through the first time. Thus, the prepared syringe filters with immobilised adsorbents were used in all regeneration experiments described in the manuscript. The nature of the syringe filter adsorption columns made it impossible to recover the materials for further characterisation after regeneration, a sacrifice made consciously to enable us to conduct the number of experiments necessary to perform the envisioned adsorption-regeneration analysis for several solvents and best-performing materials.

The preliminary solvent selection experiments were conducted as single repetitions per material and solvent (including blank control filters). The protocol consisted of several steps, where first we passed 2 mL of PFAS stock solution (2 μg L^{-1} starting concentration) and captured the filtrate in an eppendorf vial. Then 1 mL of ultrapure water is passed as a wash step and captured, followed by 1 mL of regeneration solvent passed and captured. The PFAS masses in each fraction are quantified by LC/MS-MS and added together to represent the "total available PFAS" mass balance from which we subtract the PFAS mass quantified in the adsorption and wash of a MOF column run to obtain the PFAS by immobilized MOF, as displayed in Equation 1:

$$PFAS_{removed}\ [ng] = \sum PFAS_{blank,average} - PFAS_{adsorption\ pass} - PFAS_{wash\ pass} \qquad \text{Equation 1}$$

Where the sum of PFAS blank averages represents the above described "total available PFAS" in a blank, obtained by adding together the three fractions of adsorption, wash, and regeneration. The removal percentage is then determined by dividing the removed PFAS mass by the "total available PFAS", as can be seen in Equation 2:

$$PFAS_{removed}\ [\%] = \frac{PFAS_{removed}\ [ng]}{\sum PFAS_{blank,average}\ [ng]} \qquad \text{Equation 2}$$

The mass of PFAS removed as determined in Equation 1 was then used as the reference for determining the regeneration performance, as displayed in Equation 3:

$$PFAS_{recovered}\ [\%] = \frac{PFAS_{regeneration}\ [ng]}{PFAS_{removed}\ [ng]} \qquad \text{Equation 3}$$

The 5 cycle regeneration experiments (conducted in duplicate) had an additional wash step (1 mL of ultrapure water) that followed the regeneration solvent, in which PFAS were also quantified and added to the mass balance. This was done mainly to prime the column before the next adsorption pass and remove any residual solvent. Furthermore, blank "columns" (*i.e.*, syringe filter without adsorbent immobilised) were also processed in an identical manner in triplicate, the mass balances of the triplicates averaged per cycle, and the average PFAS mass balances per cycle used to determine adsorption performance of a corresponding MOF cycle. This means that we also determine the regeneration performance per cycle, as is presented in Equation 4:

$$PFAS_{recovered}, i^{th} cycle\ [\%] = \frac{PFAS_{regeneration}, i^{th} cycle\ [ng]}{PFAS_{removed}, i^{th} cycle\ [ng]} \qquad \text{Equation 4}$$

Occasionally, slightly higher than 100% recovery rates were observed for PFOA and PFOS in advanced cycles, presumably by recovery of fractions of PFAS not recovered in previous cycles. The total PFAS recovered in each cycle did not account for all PFAS removed in previous cycles.

Differences between the UiO-66-X and the PDMS-modified UiO-66-X analogues (UiO-66-X-PDMS) (X = H, $(F)_4$, NO_2, and NH_2):

PXRD patterns in Figure S1 reveal isostructural nature. TGA traces in Figures S6 reveal that post (the exchanged solvent) methanol removal, the thermal stability order of the four **UiO-66-X-PDMS** analogues as: **UiO-66-NO_2-PDMS** > **UiO-66-$(F)_4$-PDMS** > **UiO-66-NH_2-PDMS** > **UiO-66-PDMS**. N_2 adsorption isotherms at 77 K confirm that PDMS coating consistently reduced microporosity (resulting in a decrease of BET surface areas: Figure S12). H_2O vapour adsorption isotherms (recorded at 298 K) for the four **UiO-66-X-PDMS** sorbents were found to be consistent with those of the four **UiO-66-X** sorbents (Figure 1H; Figure S29). Starting from hydrophilic water contact angles ≈ 0° for all **UiO-66-X**, the PDMS coated **UiO-66-X-PDMS** analogues exhibited high hydrophobicity along the order: **UiO-66-NH_2-PDMS** (155.7) > **UiO-66-NO_2-PDMS** (152.3) > **UiO-66-PDMS** (150.7) > **UiO-66-$(F)_4$-PDMS** (147.8) (Figure S30).[19] SEM micrographs at the pre- PDMS coating and the post- PDMS coating stages were captured to examine the consistency of polycrystalline morphologies, that is, the crystal habits alongside those of **UiO-66-X** and **UiO-66-X-PDMS**.[19]

Differences between the UiO-66-X and the OS-modified UiO-66-X analogues:

PXRD patterns in Figure 1F and S2 reveal isostructural nature. TGA traces in Figures S7 reveal that post (the exchanged solvent) methanol removal, the thermal stability order of the four **UiO-66-X-OS** analogues as: **UiO-66-OS** > **UiO-66-NO_2-OS** ≈ **UiO-66-$(F)_4$-OS** ≈ **UiO-66-NH_2-OS**. N_2 adsorption isotherms at 77 K confirm that OS coating consistently reduced microporosity (resulting in a decrease of BET surface areas: Figure S12). H_2O vapour adsorption isotherms (recorded at 298 K) for the four **UiO-66-X-OS** sorbents were found to be consistent with those of the four **UiO-66-X** sorbents (Figure 1H). Starting from hydrophilic water contact angles ≈ 0° for all **UiO-66-X**, the OS coated **UiO-66-X-OS** analogues exhibited high hydrophobicity along the order: **UiO-66-NH_2-OS** (148.6) > **UiO-66-NO_2-OS** (145.7) > **UiO-66-OS** (142.8) > **UiO-66-$(F)_4$-OS** (141.2) (Figure 1I).[19] SEM micrographs at the pre- OS coating and the post- OS coating stages were captured to examine the consistency of polycrystalline morphologies, that is, the crystal habits alongside those of **UiO-66-X** and **UiO-66-X-OS** (Figure 1J; Figures S31, S32).

Supporting Figures.

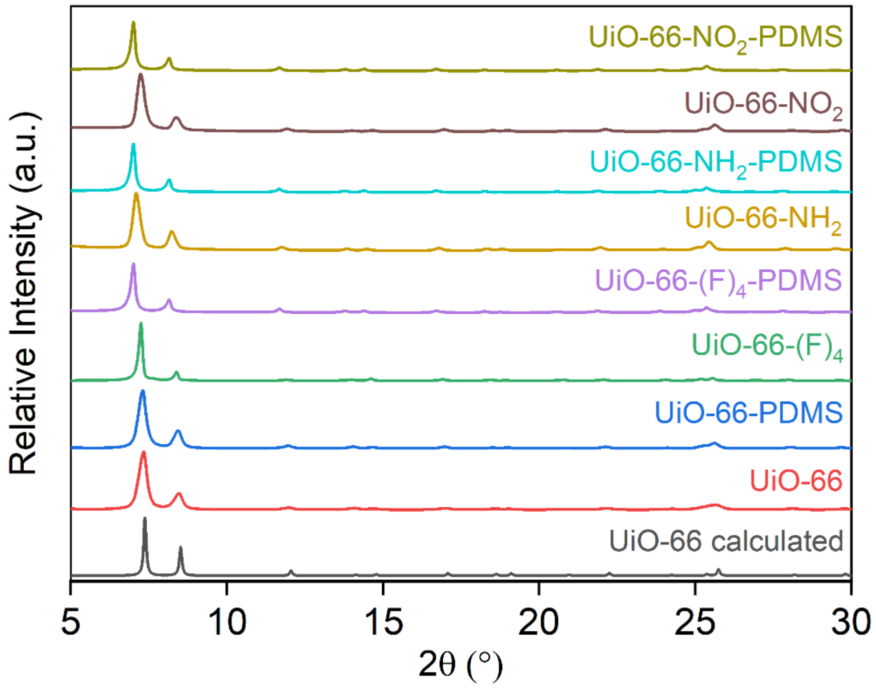

Figure S1. UiO-66-X and **UiO-66-X-PDMS** (X= H, (F)$_4$, NO_2 and NH_2) powder X-ray diffractograms after synthesis, plotted alongside the simulated **UiO-66** powder X-ray pattern reported in Cambridge Structural Database Refcode RUBTAK03.[20]

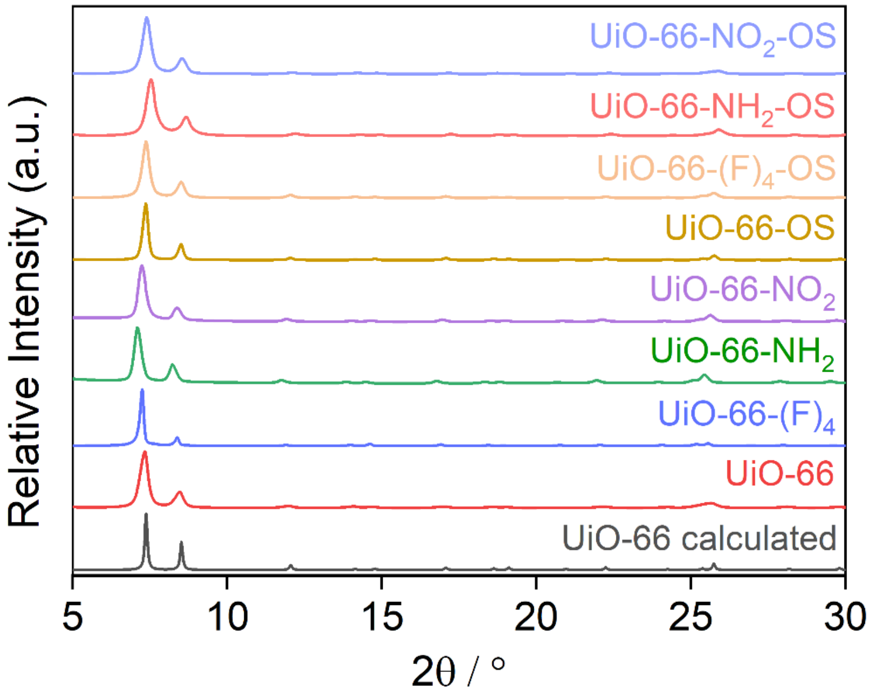

Figure S2. UiO-66-X and **UiO-66-X-OS** (X= H, (F)$_4$, NO_2 and NH_2) powder X-ray diffractograms after synthesis, plotted alongside the simulated **UiO-66** powder X-ray pattern reported in Cambridge Structural Database Refcode RUBTAK03.[20]

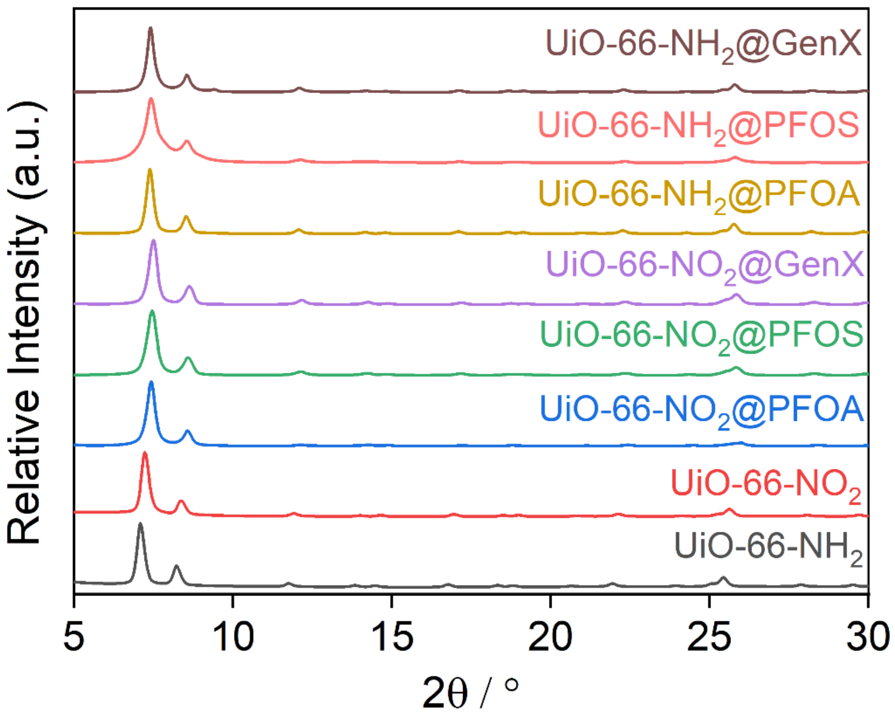

Figure S3. UiO-66-X (X= NO_2 and NH_2) powder X-ray diffractograms after synthesis, plotted alongside the corresponding phases obtained post-PFAS adsorption (PFAS: PFOA, PFOS and Genx).

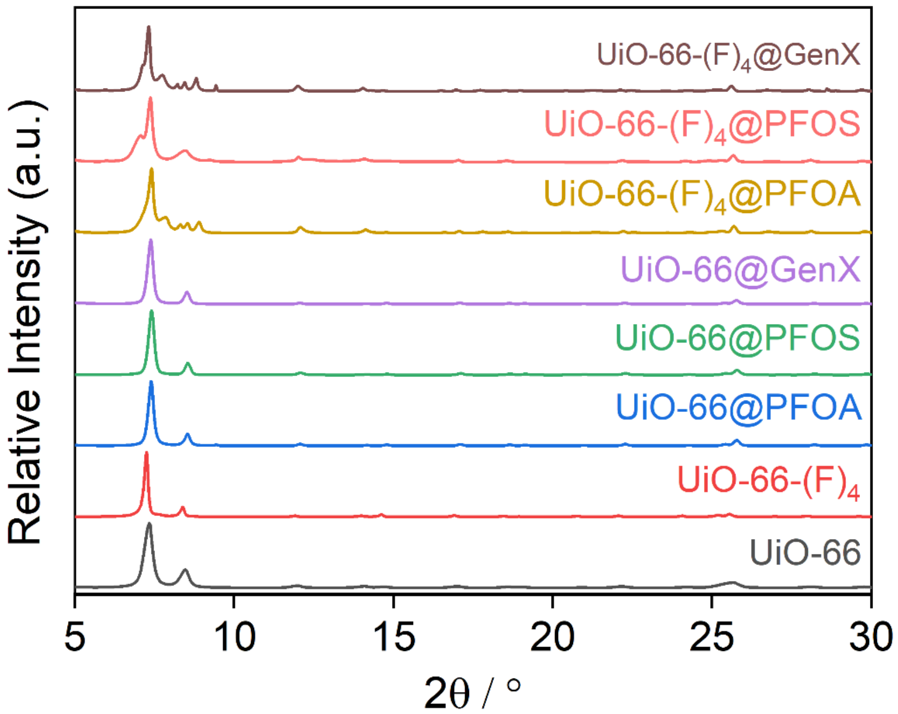

Figure S4. UiO-66-X (X= H and $(F)_4$) powder X-ray diffractograms after synthesis, plotted alongside the corresponding phases obtained post-PFAS adsorption (PFAS: PFOA, PFOS and Genx).

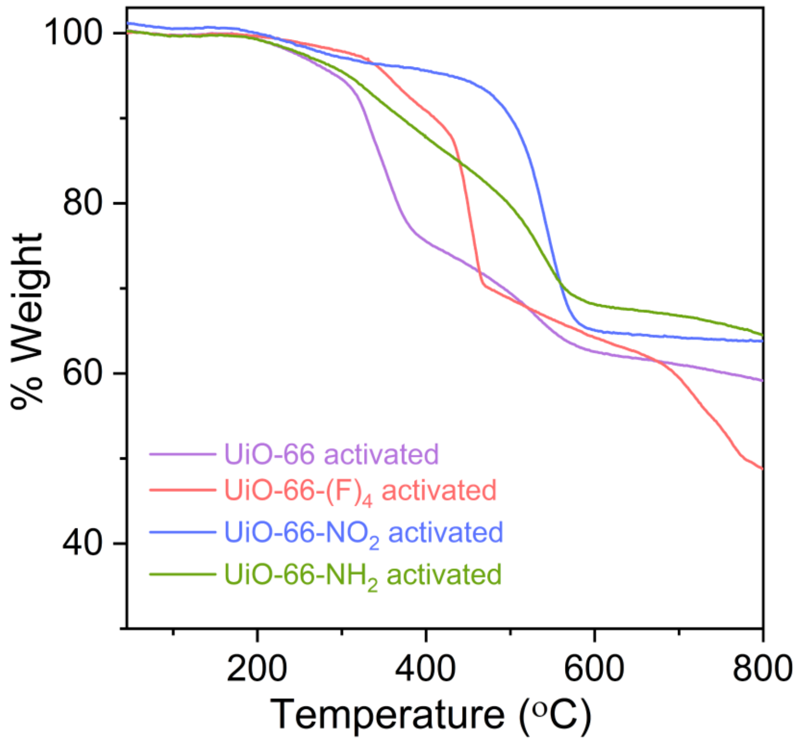

Figure S5. Thermogravimetric analysis profiles for **UiO-66-X** (X = H, $(F)_4$, NO_2, NH_2).

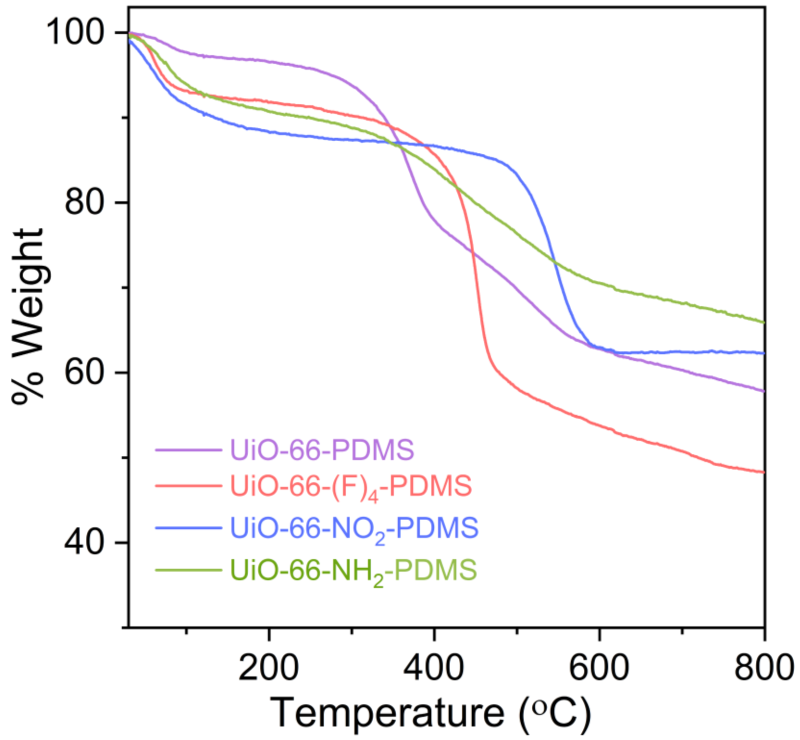

Figure S6. Thermogravimetric analysis profiles for **UiO-66-X-PDMS** (X = H, $(F)_4$, NO_2, NH_2) (A), the PDMS protected MOFs.

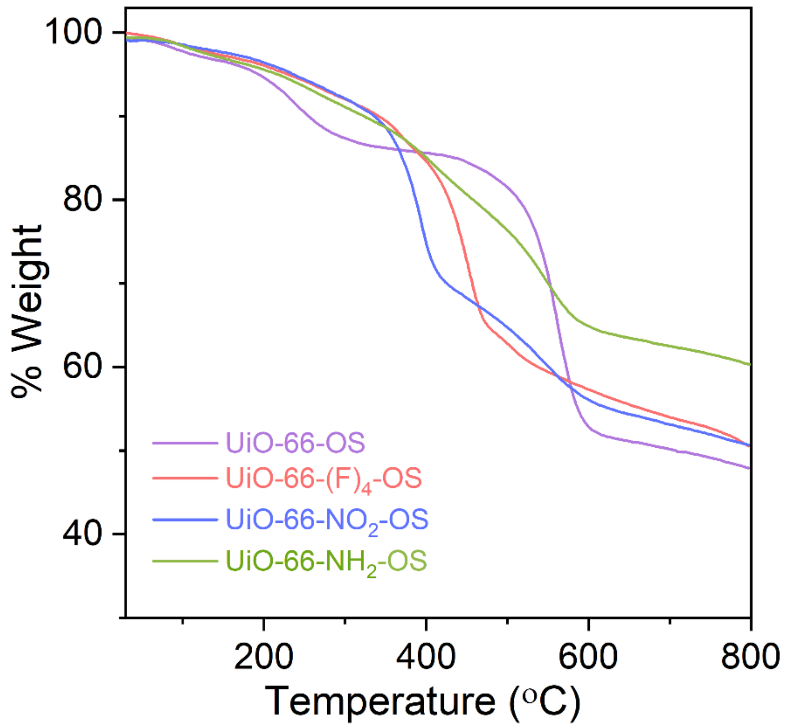

Figure S7. Thermogravimetric analysis profiles for **UiO-66-X-OS** (X = H, $(F)_4$, NO_2, NH_2) (A), the OS protected MOFs.

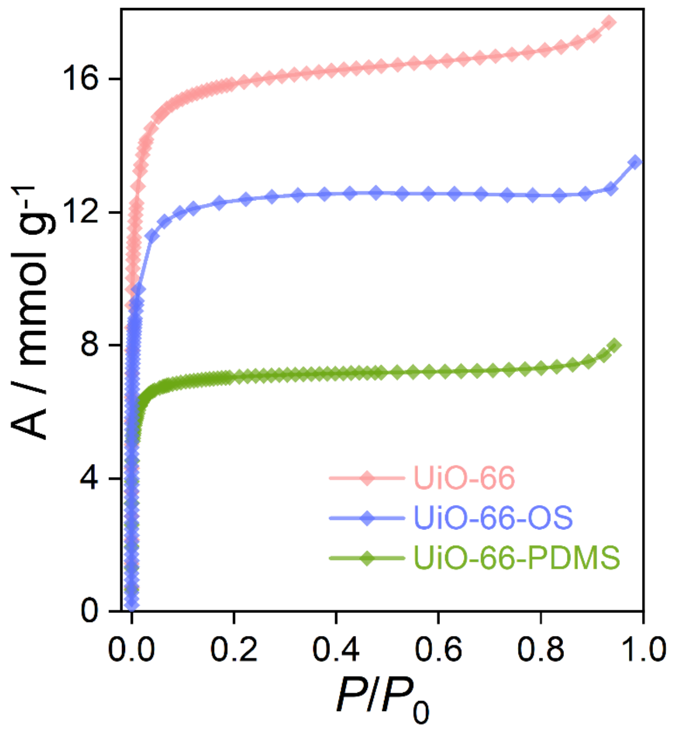

Figure S8. N_2 adsorption isotherms recorded at 77 K for **UiO-66**, **UiO-66-PDMS** and **UiO-66-OS**.

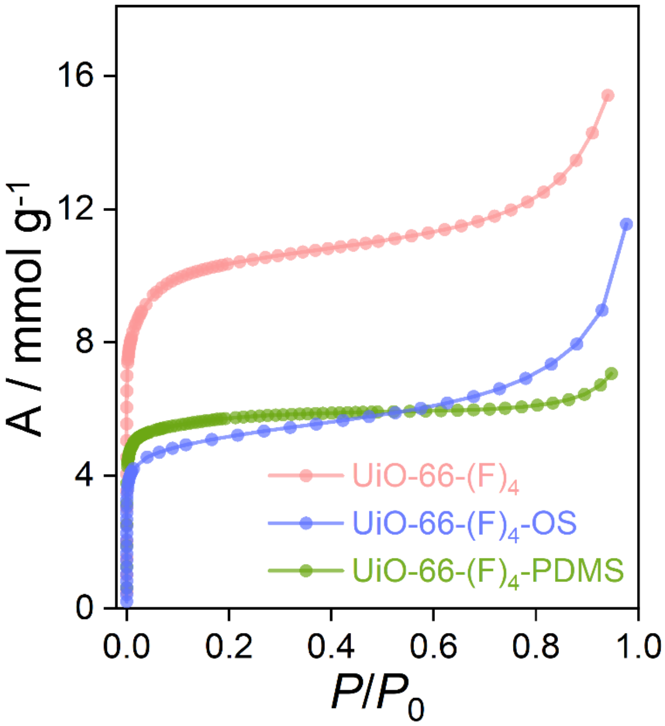

Figure S9. N_2 adsorption isotherms recorded at 77 K for **UiO-66-(F)$_4$**, **UiO-66-(F)$_4$-PDMS** and **UiO-66-(F)$_4$-OS**.

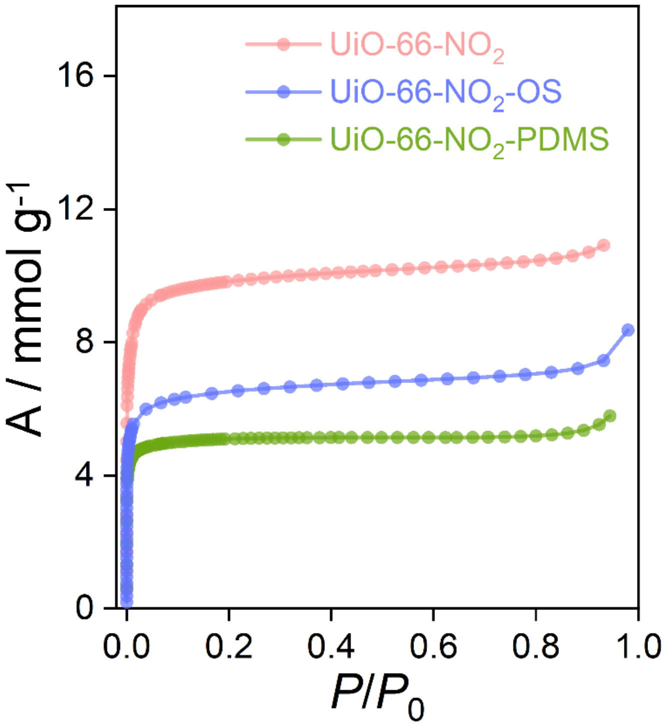

Figure S10. N_2 adsorption isotherms recorded at 77 K for **UiO-66-NO$_2$**, **UiO-66-NO$_2$-PDMS** and **UiO-66-NO$_2$-OS**.

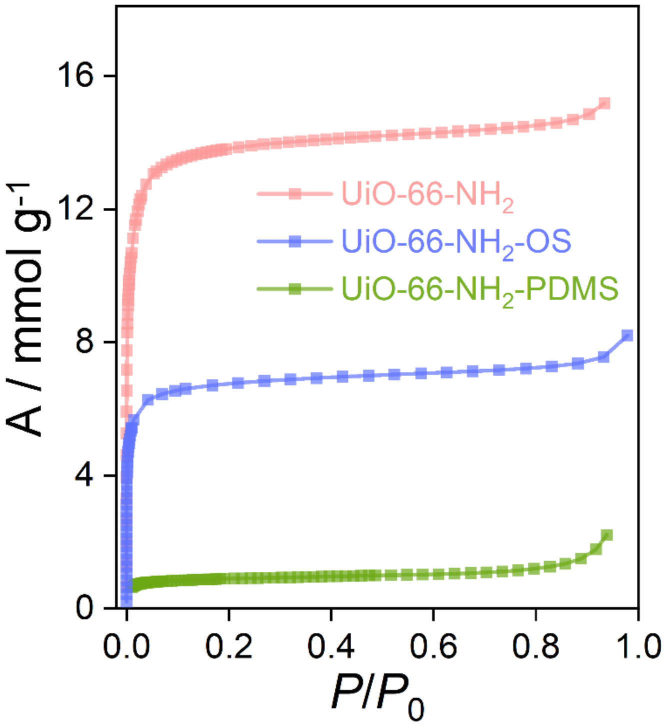

Figure S11. N_2 adsorption isotherms recorded at 77 K for **UiO-66-NH$_2$**, **UiO-66-NH$_2$-PDMS** and **UiO-66-NH$_2$-OS**.

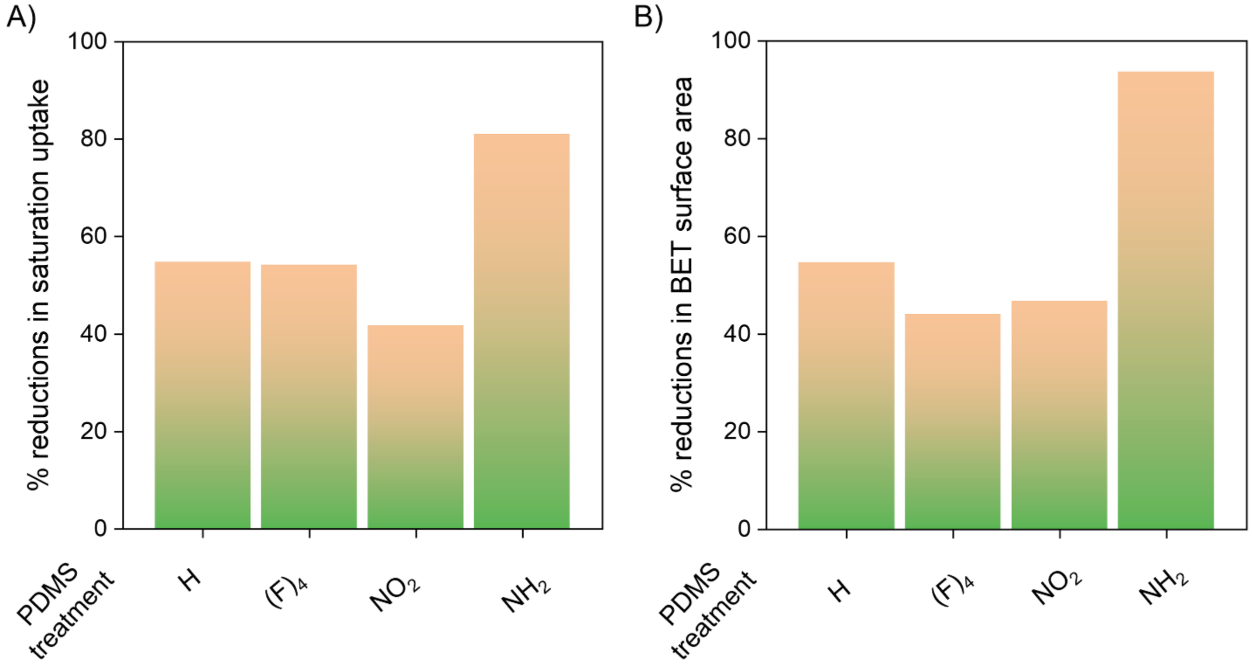

Figure S12. (A) % reduction of the nitrogen saturation uptakes (each recorded at 77 K and ≈ 1 bar) upon PDMS treatment. (B) % reduction of the BET surface areas upon PDMS treatment.

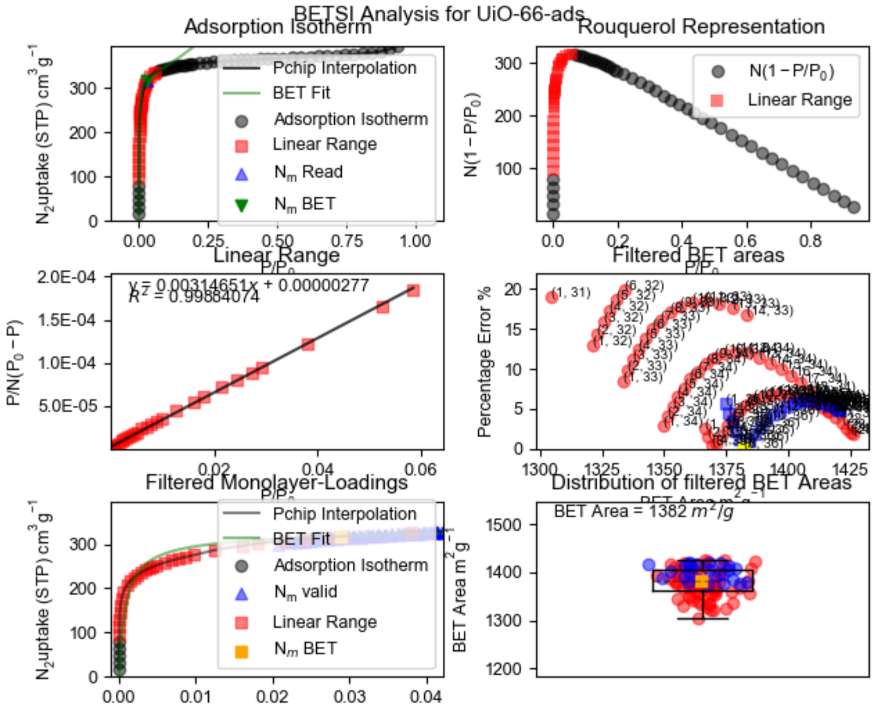

Figure S13. BETSI fitting and BET area calculations for **UiO-66**.

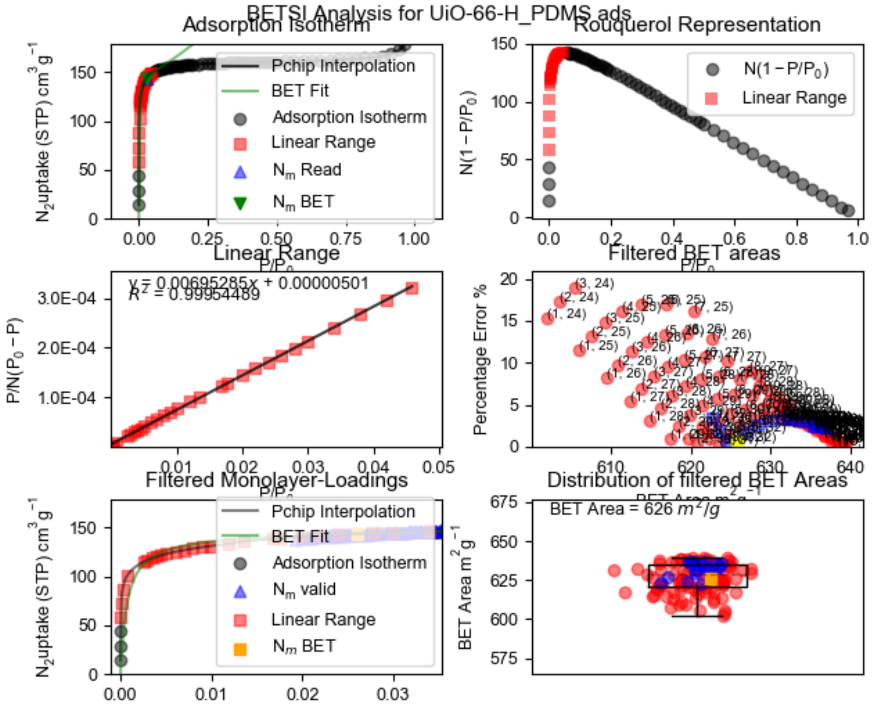

Figure S14. BETSI fitting and BET area calculations for **UiO-66-PDMS**.

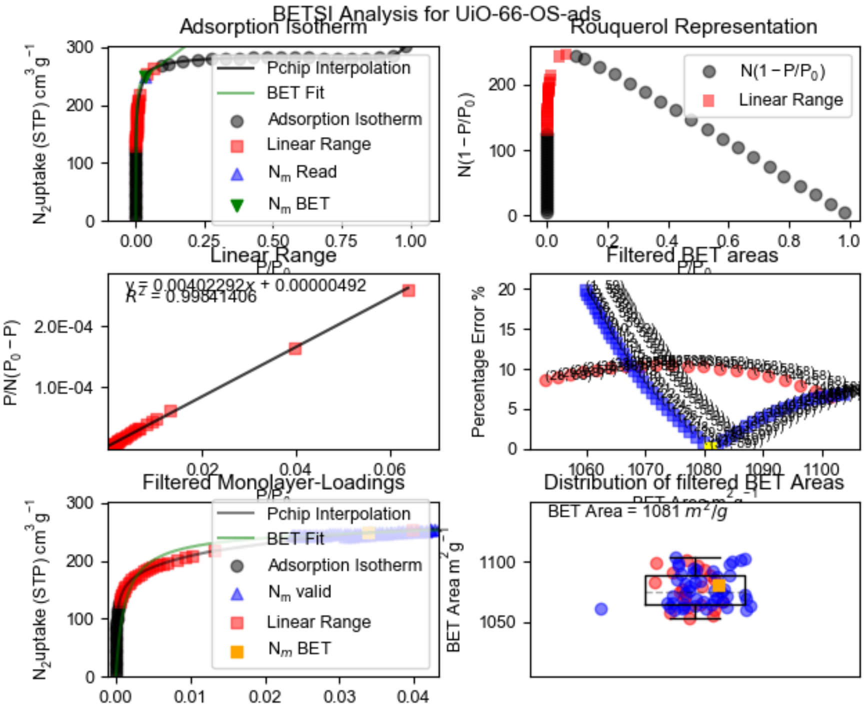

Figure S15. BETSI fitting and BET area calculations for **UiO-66-OS**.

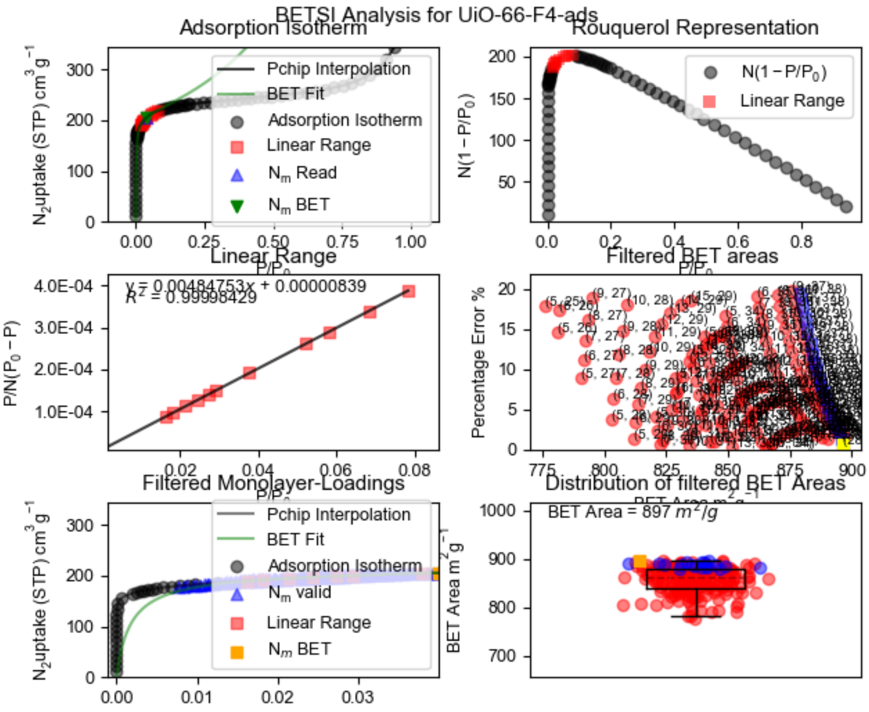

Figure S16. BETSI fitting and BET area calculations for **UiO-66-(F)$_4$**.

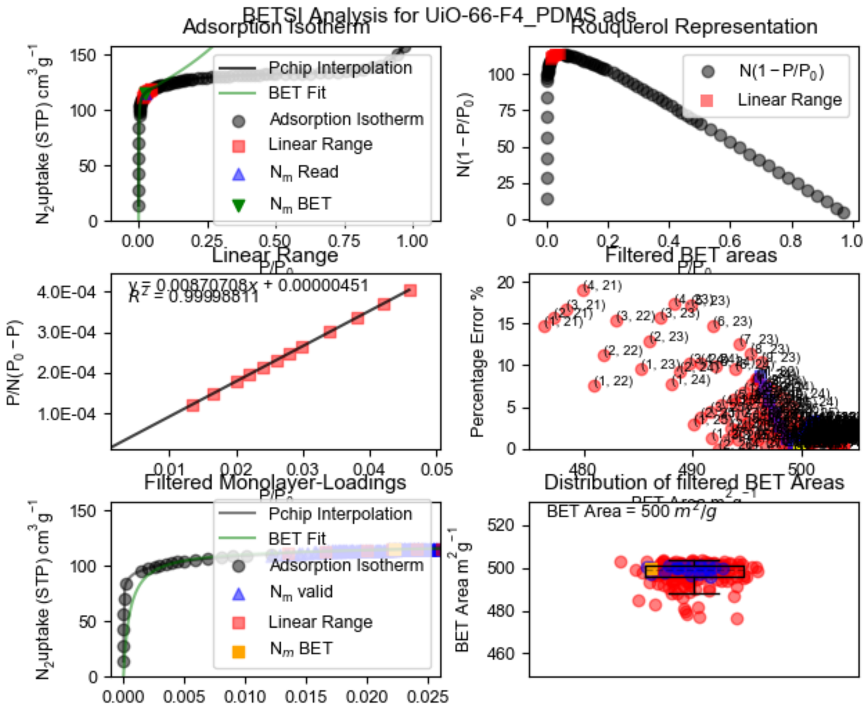

Figure S17. BETSI fitting and BET area calculations for **UiO-66-(F)$_4$-PDMS**.

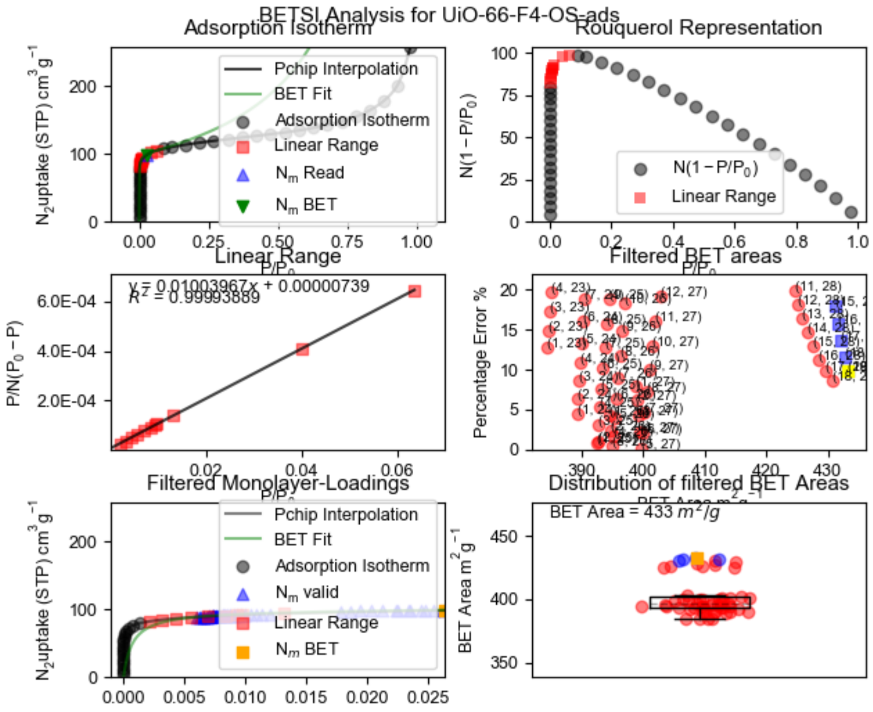

Figure S18. BETSI fitting and BET area calculations for **UiO-66-(F)$_4$-OS**.

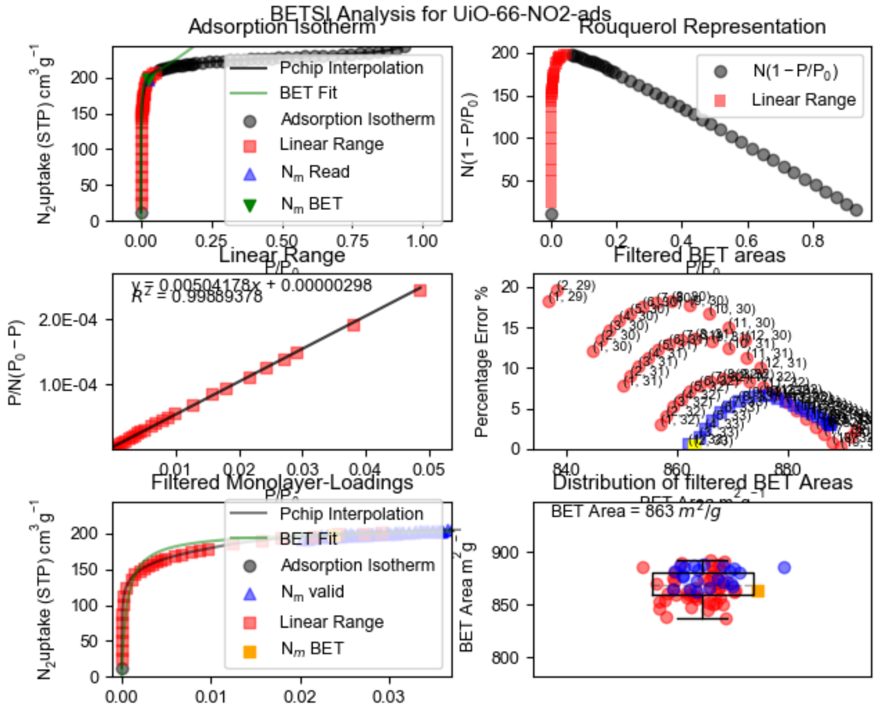

Figure S19. BETSI fitting and BET area calculations for **UiO-66-NO$_2$**.

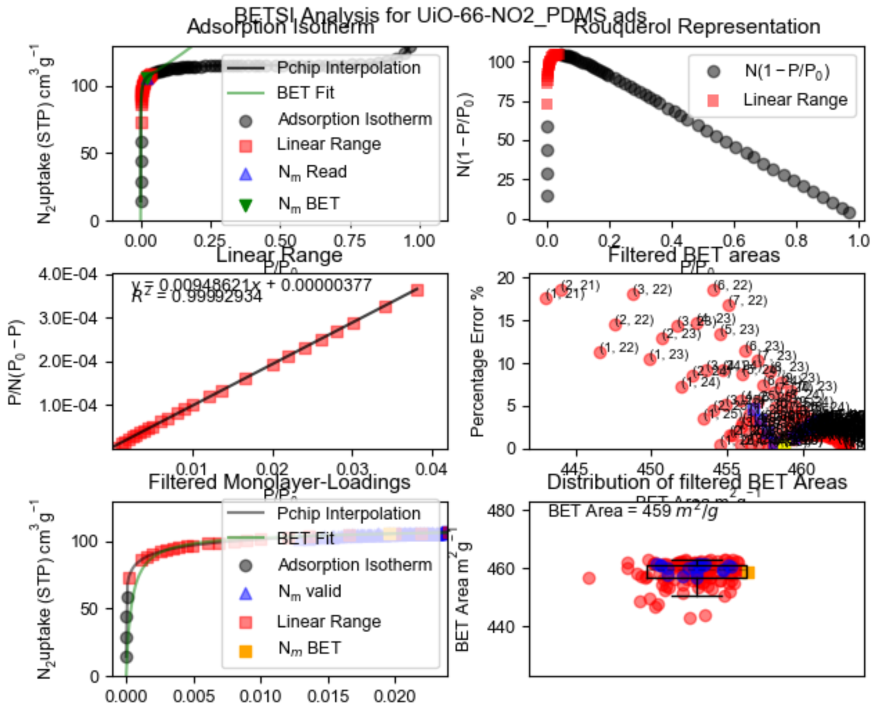

Figure S20. BETSI fitting and BET area calculations for **UiO-66-NO$_2$-PDMS**.

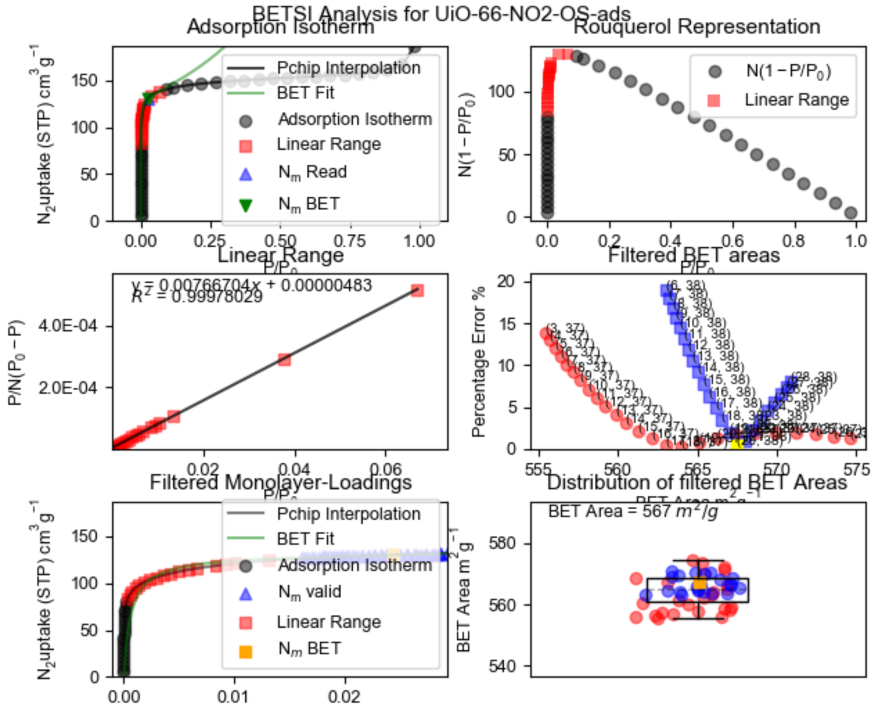

Figure S21. BETSI fitting and BET area calculations for **UiO-66-NO$_2$-OS**.

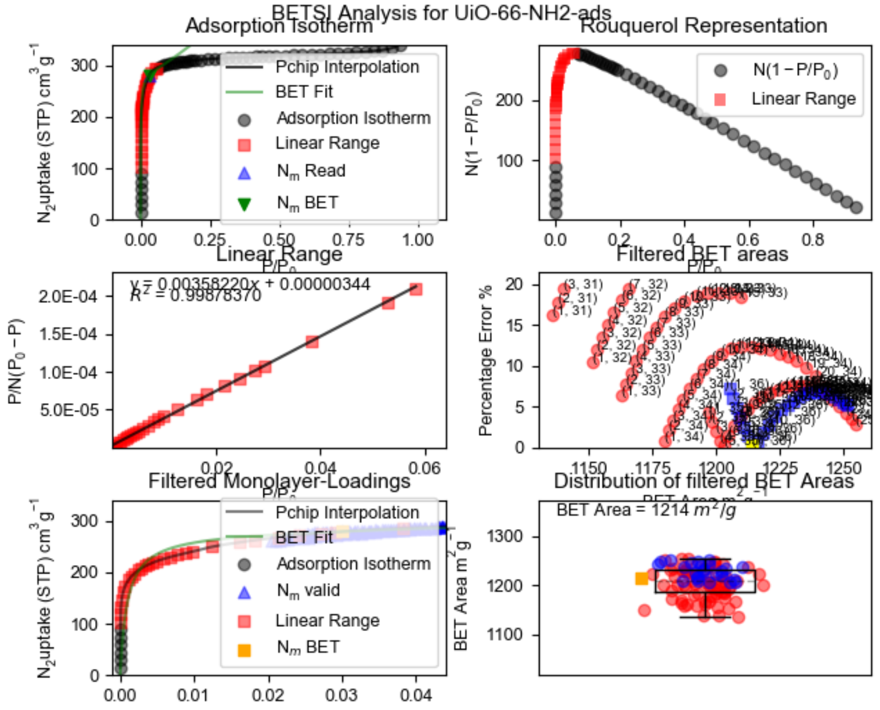

Figure S22. BETSI fitting and BET area calculations for **UiO-66-NH$_2$**.

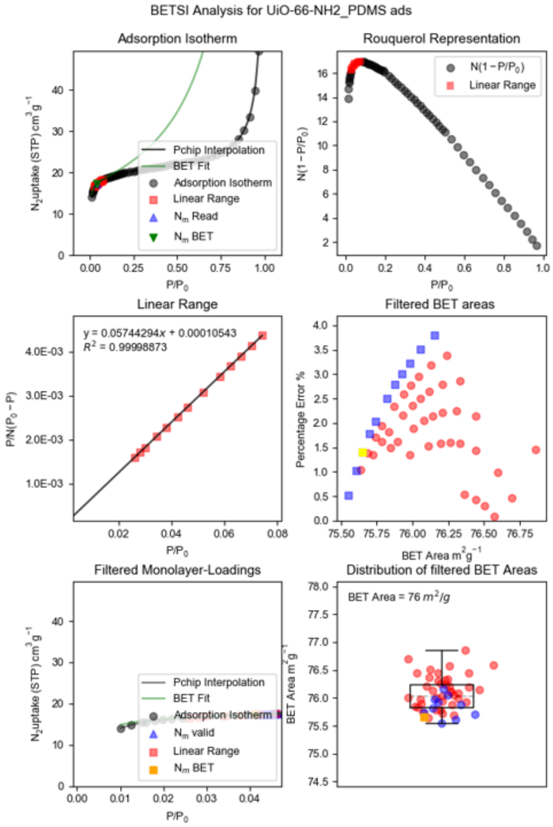

Figure S23. BETSI fitting and BET area calculations for **UiO-66-NH$_2$-PDMS**.

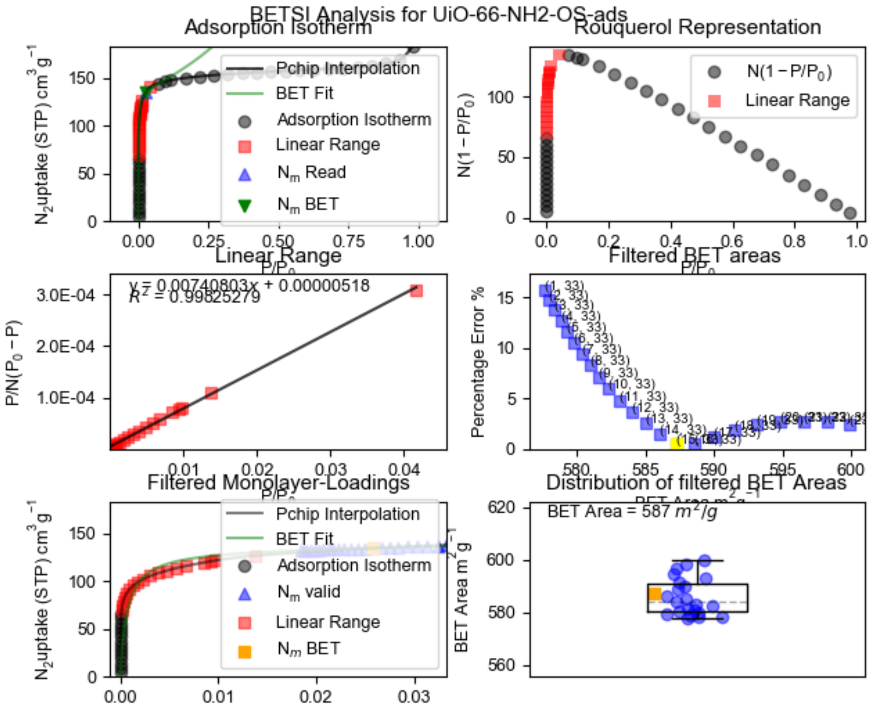

Figure S24. BETSI fitting and BET area calculations for **UiO-66-NH$_2$-OS**.

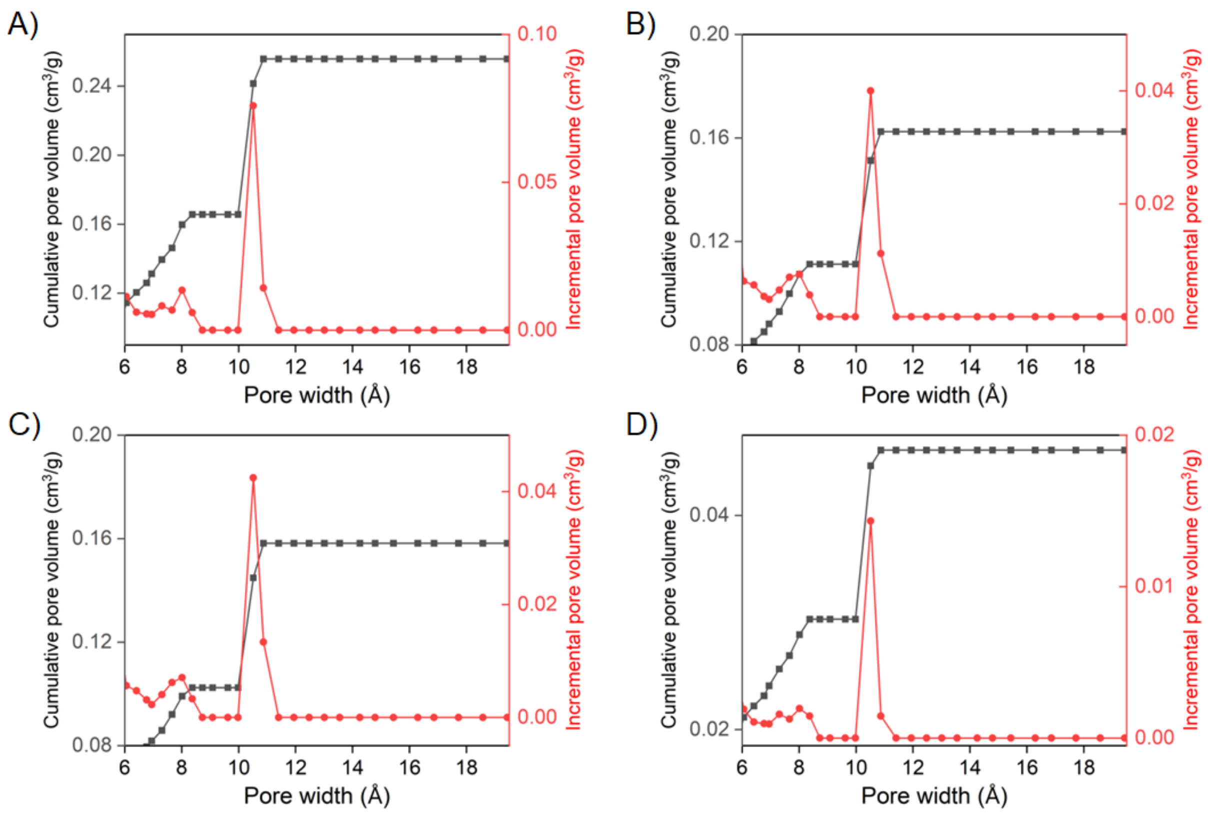

Figure S25. Pore size distribution profiles for A) **UiO-66**; B) **UiO-66@PFOA**; C) **UiO-66@PFOS**; D) **UiO-66@GenX** were obtained by fitting the NLDFT model to the respective 77 K N_2 adsorption branches.

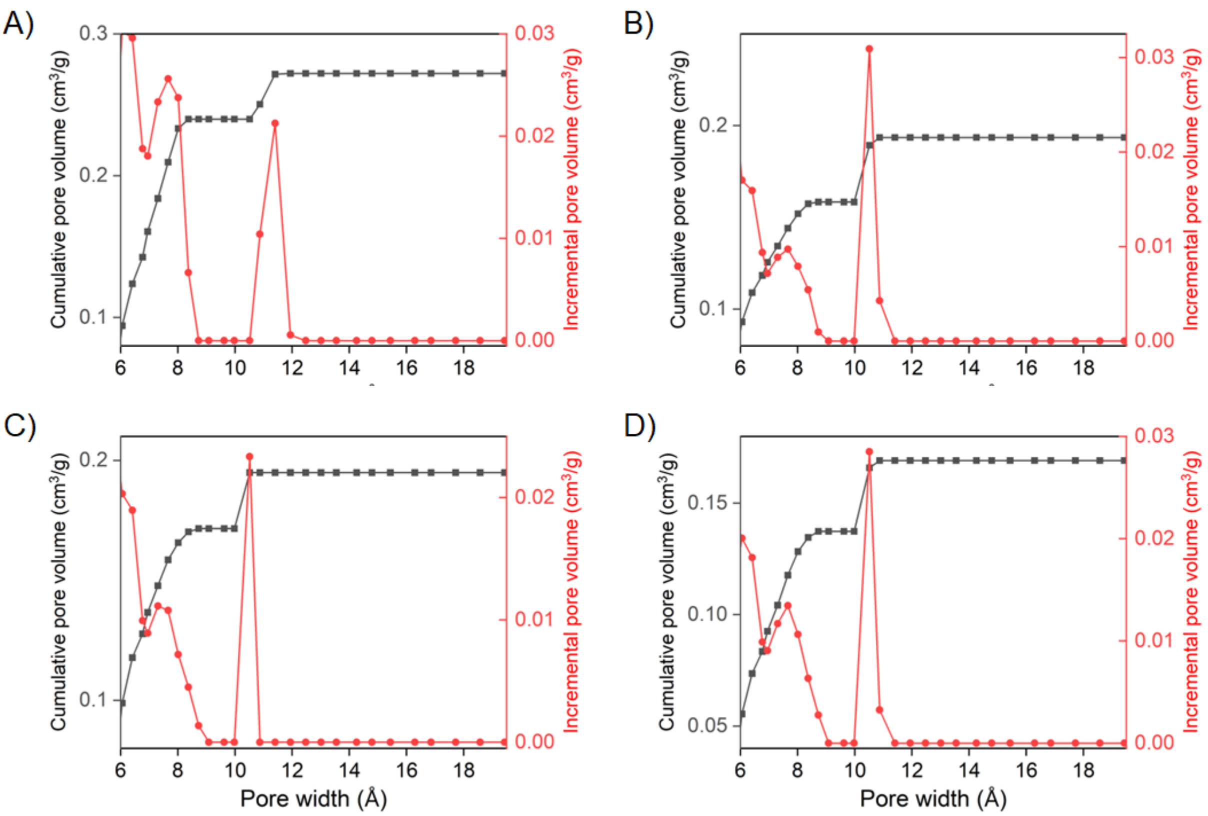

Figure S26. Pore size distribution profiles for A) **UiO-66-(F)$_4$**; B) **UiO-66-(F)$_4$@PFOA**; C) **UiO-66-(F)$_4$@PFOS**; D) **UiO-66-(F)$_4$@GenX** were obtained by fitting the NLDFT model to the respective 77 K N_2 adsorption branches.

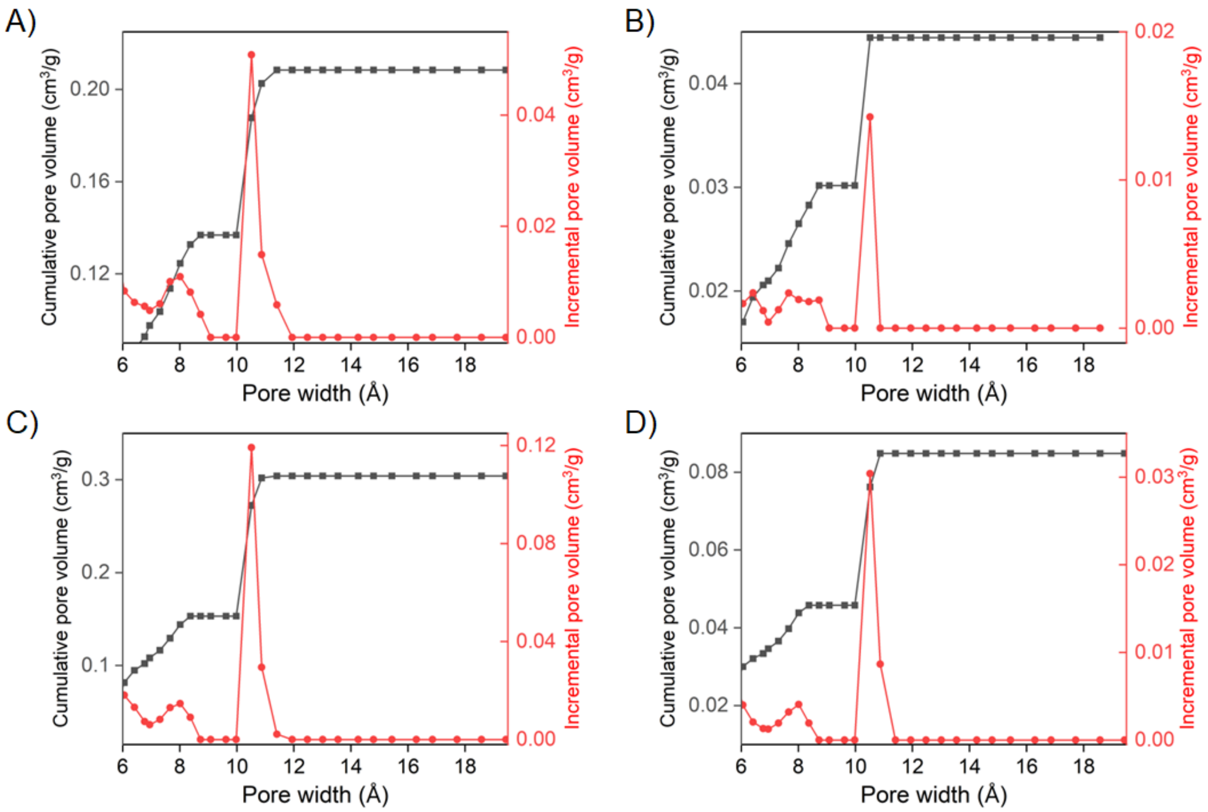

Figure S27. Pore size distribution profiles for A) **UiO-66-NO_2**; B) **UiO-66-NO_2@PFOA**; C) **UiO-66-NO_2@PFOS**; D) **UiO-66-NO_2@GenX** were obtained by fitting the NLDFT model to the respective 77 K N_2 adsorption branches.

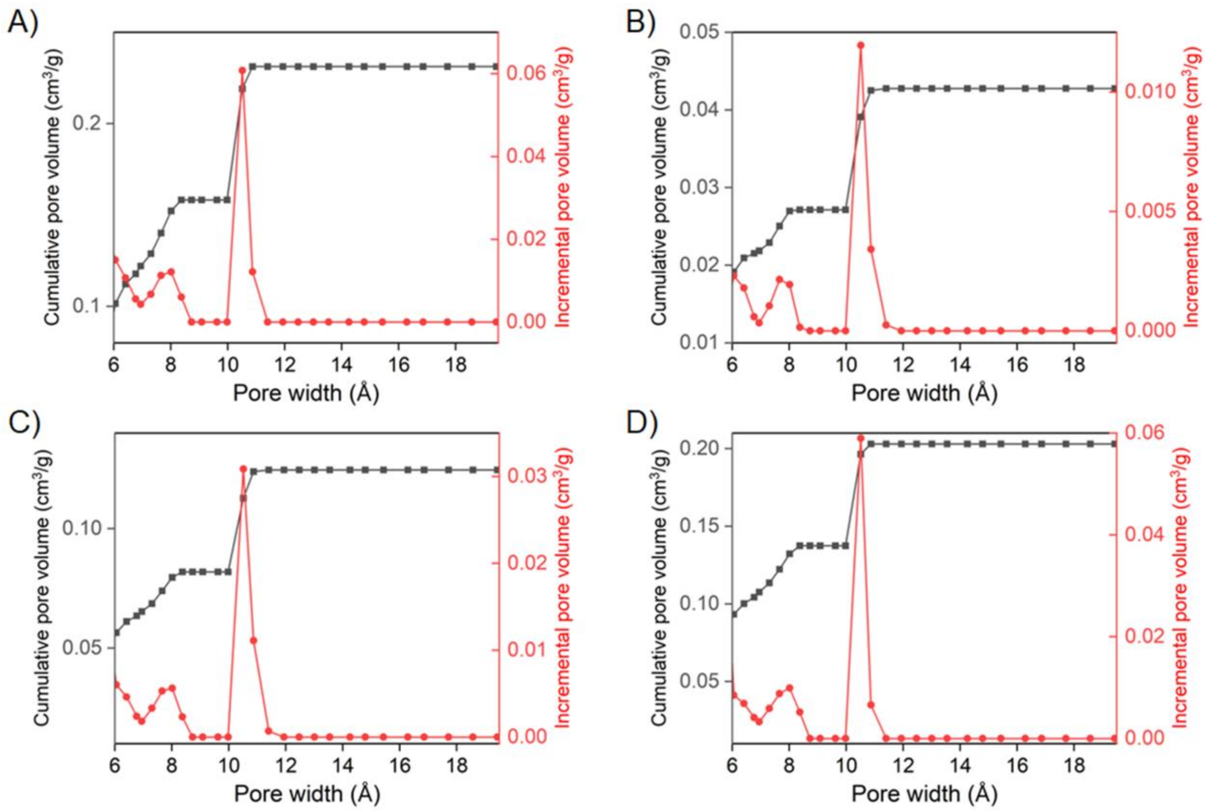

Figure S28. Pore size distribution profiles for A) **UiO-66-NH_2**; B) **UiO-66-NH_2@PFOA**; C) **UiO-66-NH_2@PFOS**; D) **UiO-66-NH_2@GenX** were obtained by fitting the NLDFT model to the respective 77 K N_2 adsorption branches.

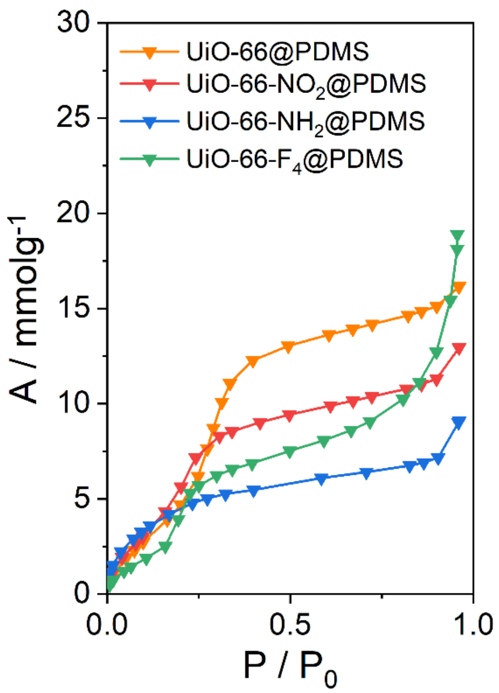

Figure S29. H_2O vapour adsorption isotherms (recorded at 298 K) for the four **UiO-66-X-PDMS** sorbents.

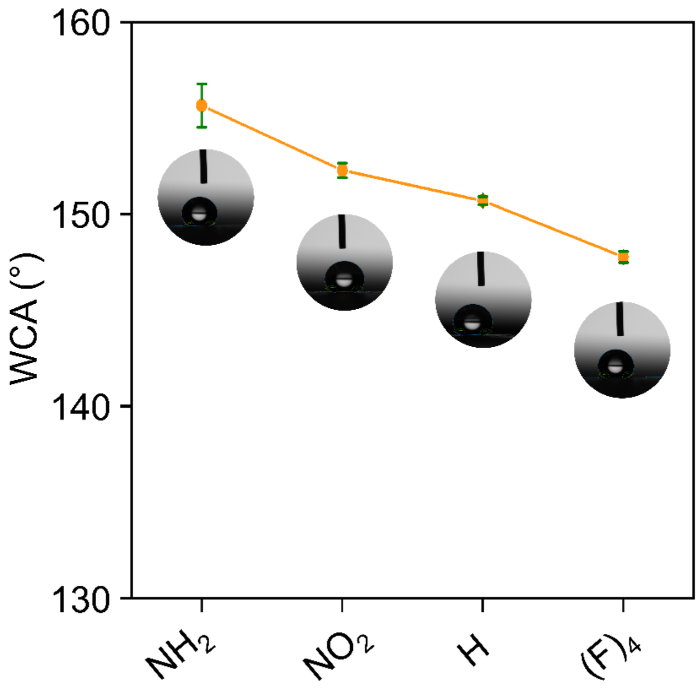

Figure S30. Static water contact angles for the four PDMS treated **UiO-66** derivatives (including the standard deviations determined from five sets of independent measurements). Insets include the water droplet pictures used for the respective measurements.

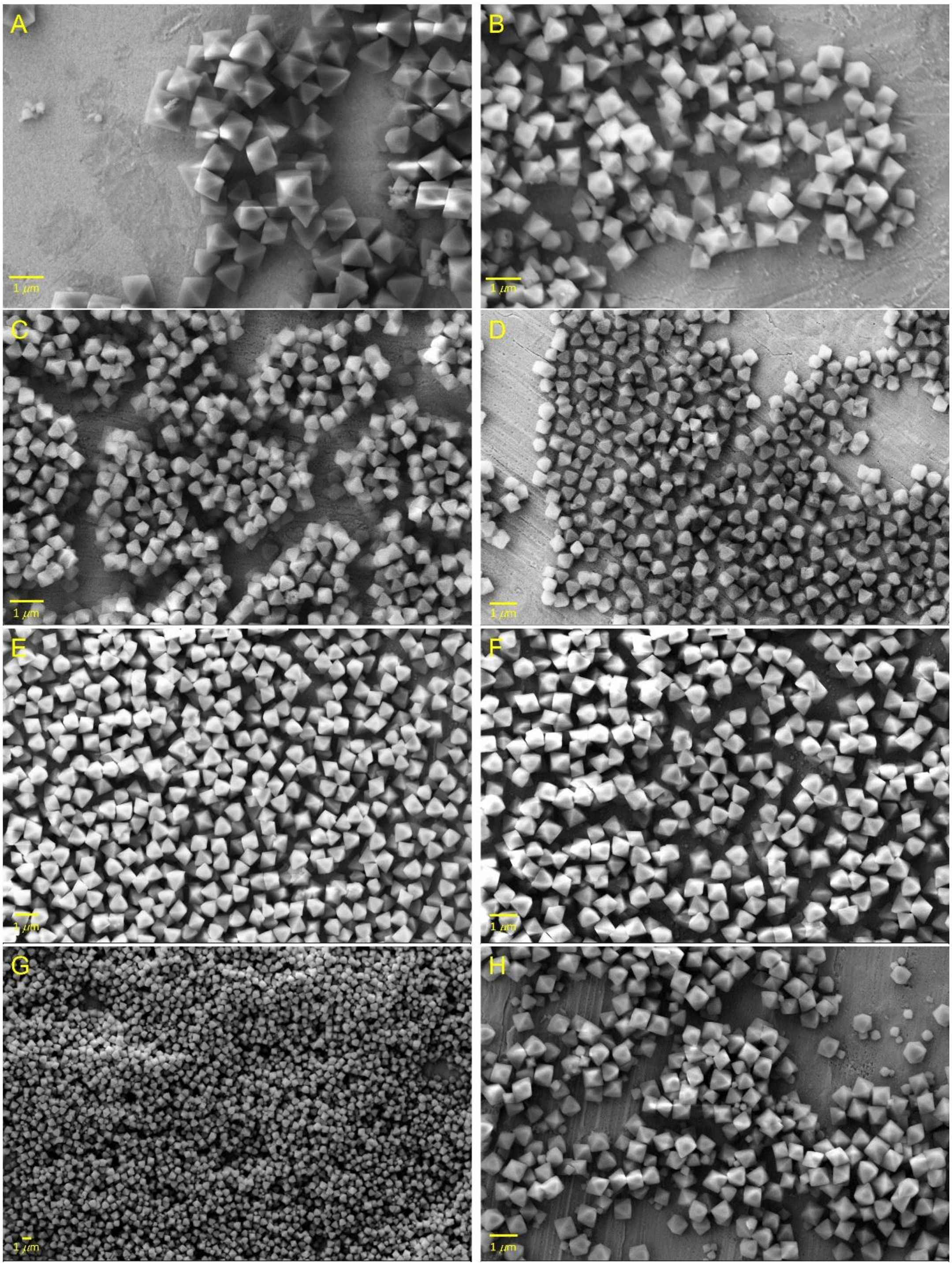

Figure S31. FE-SEM micrographs of the observed morphologies for **UiO-66**, **UiO-66-NO$_2$**, **UiO-66-NH$_2$**, **UiO-66-(F)$_4$**: A, C, E, G, respectively, and those for the corresponding PDMS protected **UiO-66-PDMS**, **UiO-66-NO$_2$-PDMS**, **UiO-66-NH$_2$-PDMS**, **UiO-66-(F)$_4$-PDMS**: B, D, F, H, respectively.

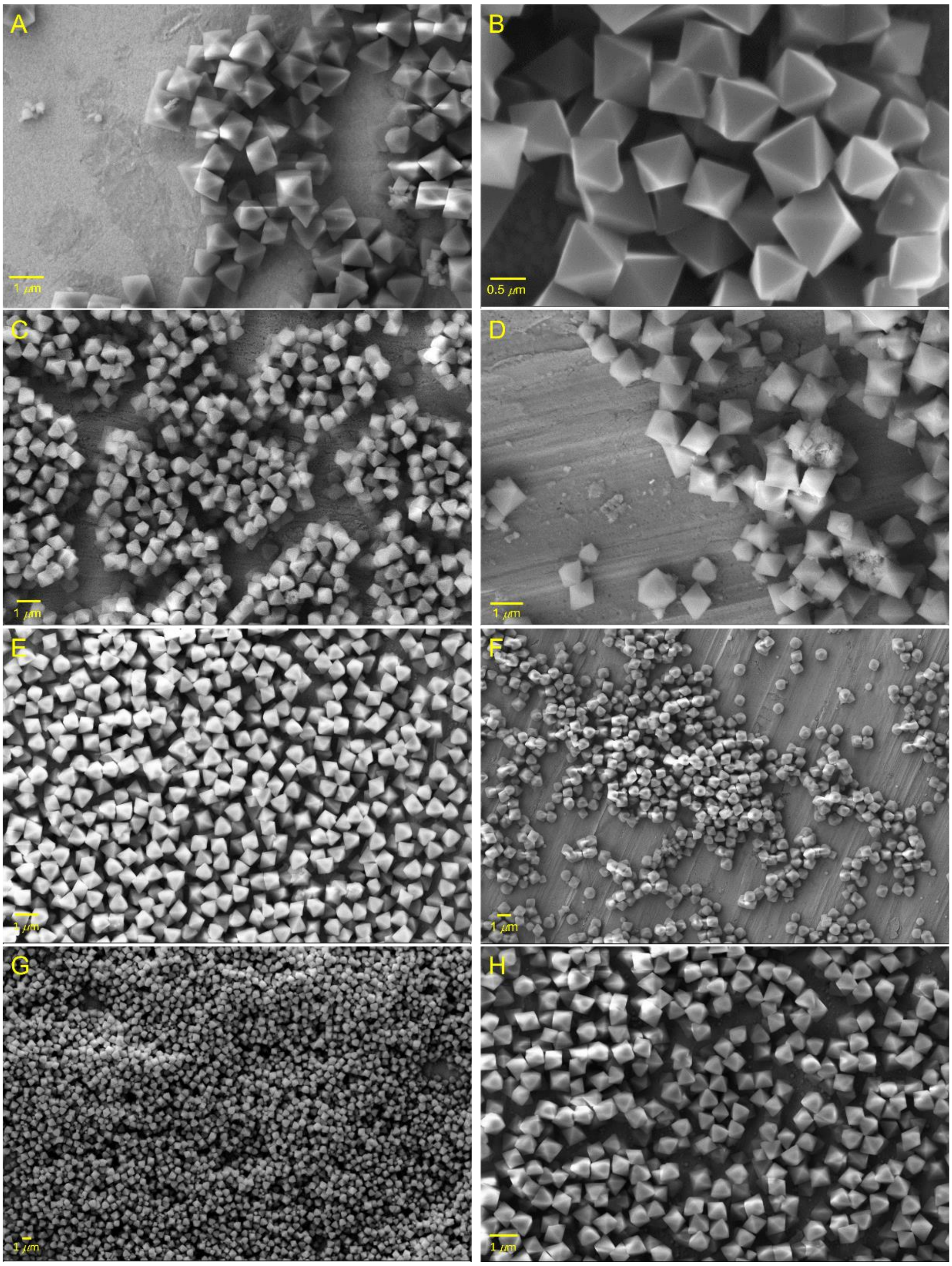

Figure S32. FE-SEM micrographs of the observed morphologies for **UiO-66**, **UiO-66-NO_2**, **UiO-66-NH_2**, **UiO-66-(F)$_4$**: A, C, E, G, respectively, and those for the corresponding OS protected **UiO-66-OS**, **UiO-66-NO_2-OS**, **UiO-66-NH_2-OS**, **UiO-66-(F)$_4$-OS**: B, D, F, H, respectively.

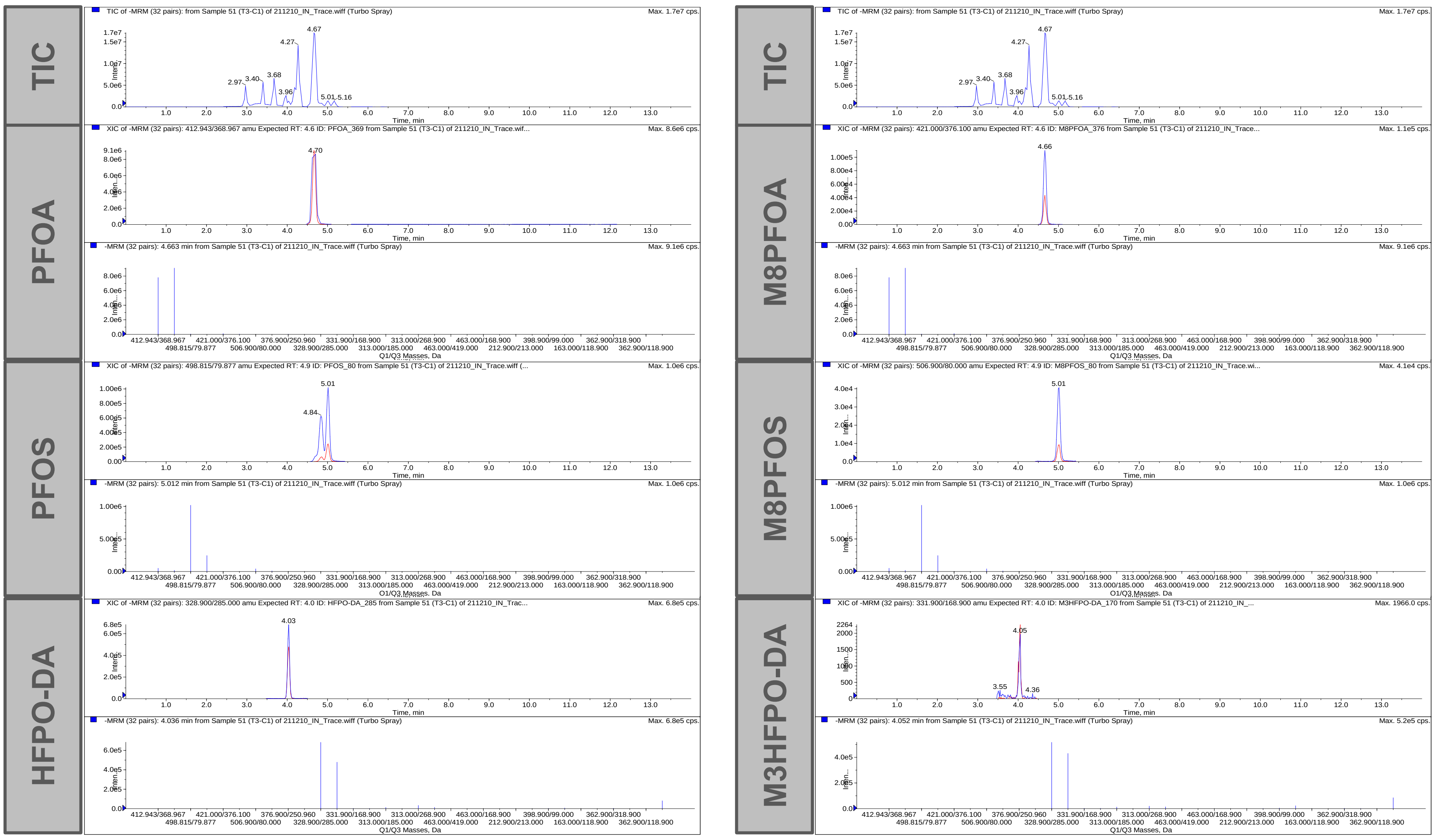

Figure S33 – ***Part 1***: TIC and mass spectra of all PFAS fragments: without adsorptive removal at trace level concentrations (in a filtered blank control sample), corresponding to Figure 2D and Table S2. The PFOS double peak was attributed to two different isomers (linear and branched);[21] integrating the double peak provided good calibration fits, and seemingly equally participated in adsorption experiments too.

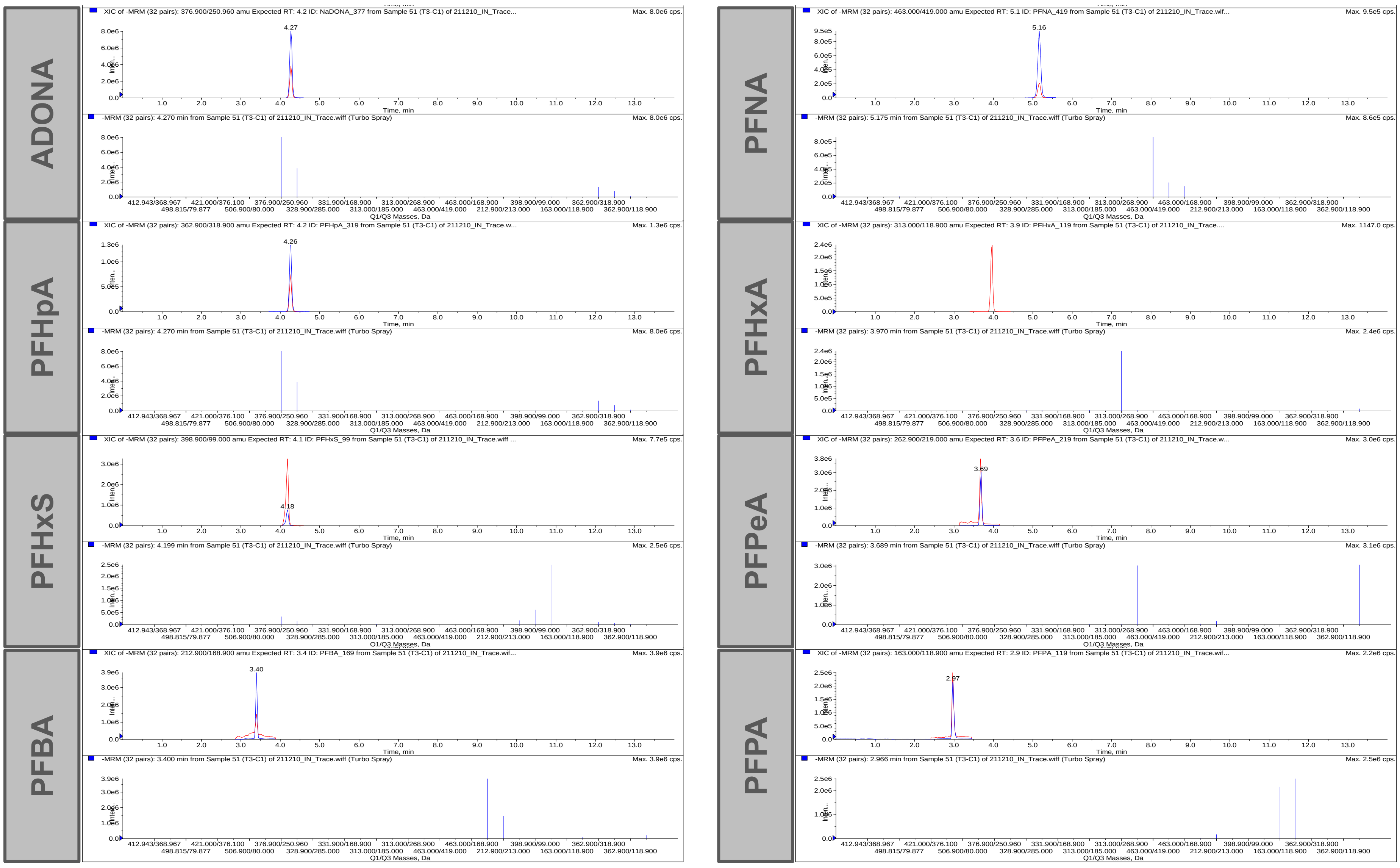

Figure S33 – ***Part 2*****:** TIC and mass spectra of all PFAS fragments: without adsorptive removal at trace level concentrations (in a filtered blank control sample), corresponding to Figure 2D and Table S2.

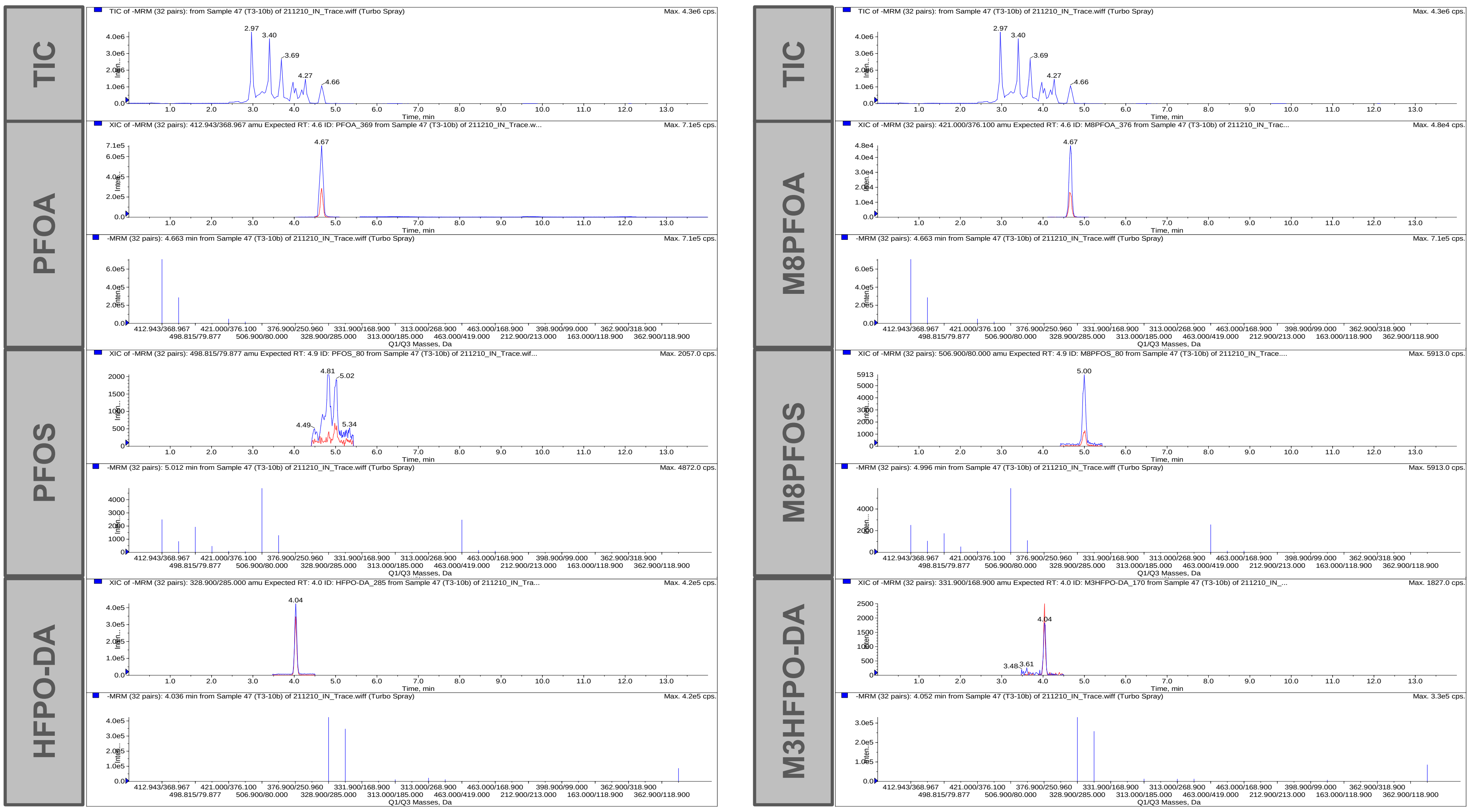

Figure S34 – ***Part 1*****:** TIC and mass spectra of all PFAS fragments: after adsorptive removal at trace level concentrations by **UiO-66-(F)$_4$-OS**, corresponding to Figure 2D and Table S2.

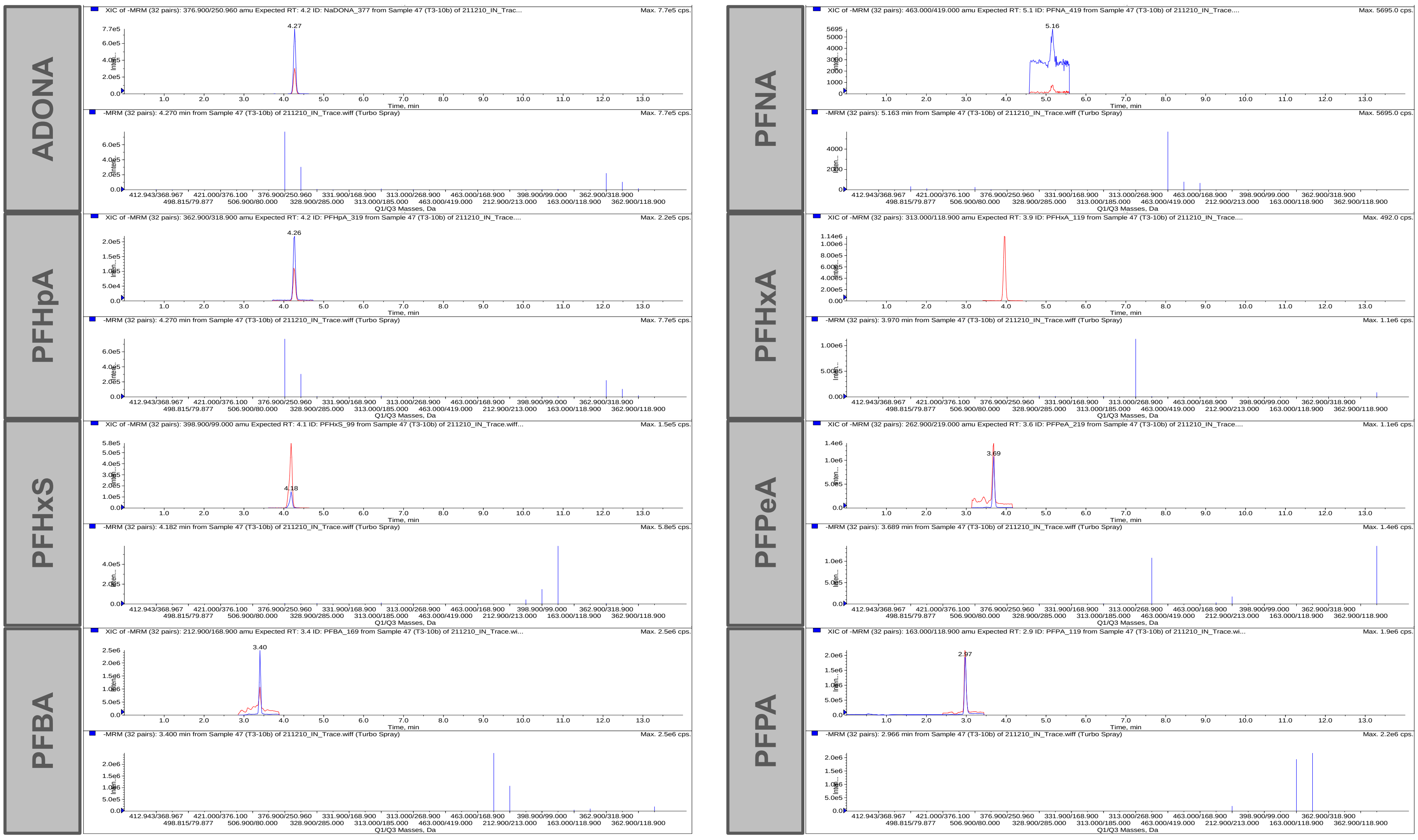

Figure S34 – ***Part 2*****:** TIC and mass spectra of all PFAS fragments: after adsorption removal at trace level concentrations by **UiO-66-(F)$_4$-OS**, corresponding to Figure 2D and Table S2.

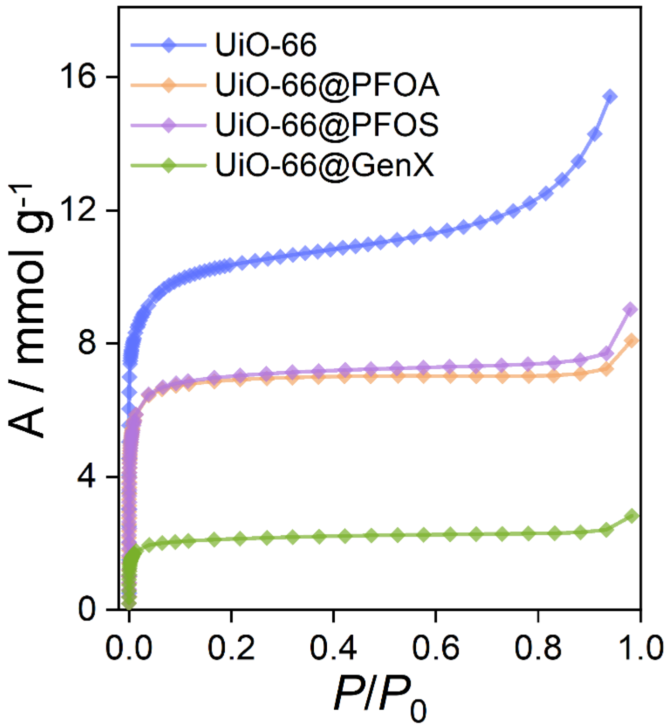

Figure S35. N_2 adsorption isotherms recorded at 77 K for **UiO-66**, **UiO-66@PFOA**, **UiO-66@PFOS** and **UiO-66@GenX**.

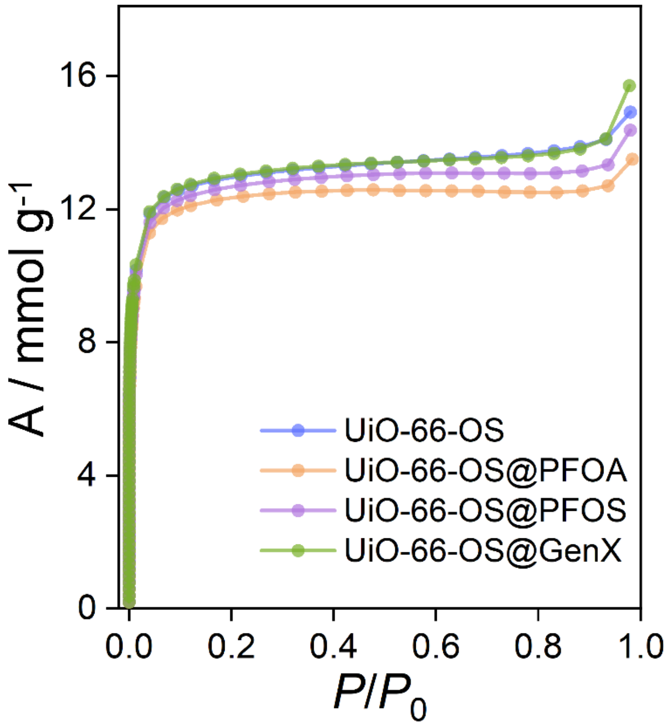

Figure S36. N_2 adsorption isotherms recorded at 77 K for **UiO-66-OS**, **UiO-66-OS@PFOA**, **UiO-66-OS@PFOS** and **UiO-66-OS@GenX**.

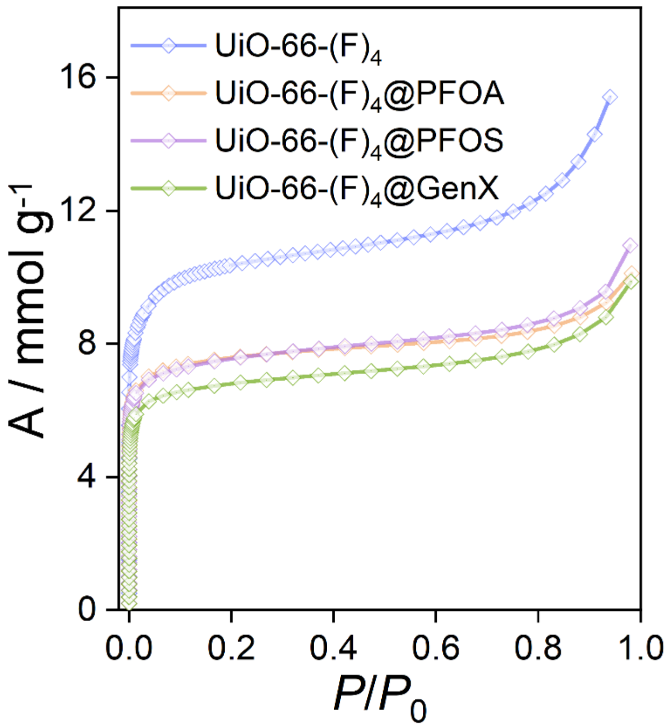

Figure S37. N_2 adsorption isotherms recorded at 77 K for **UiO-66-(F)$_4$**, **UiO-66-(F)$_4$@PFOA** and **UiO-66-(F)$_4$@PFOS** and **UiO-66-(F)$_4$@GenX**.

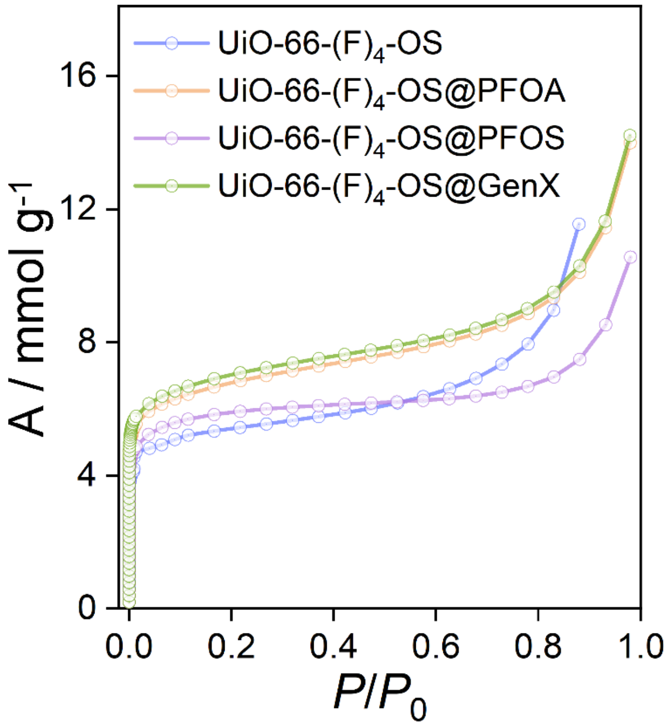

Figure S38. N_2 adsorption isotherms recorded at 77 K for **UiO-66-(F)$_4$-OS**, **UiO-66-(F)$_4$-OS@PFOA** and **UiO-66-(F)$_4$-OS@PFOS** and **UiO-66-(F)$_4$-OS@GenX**.

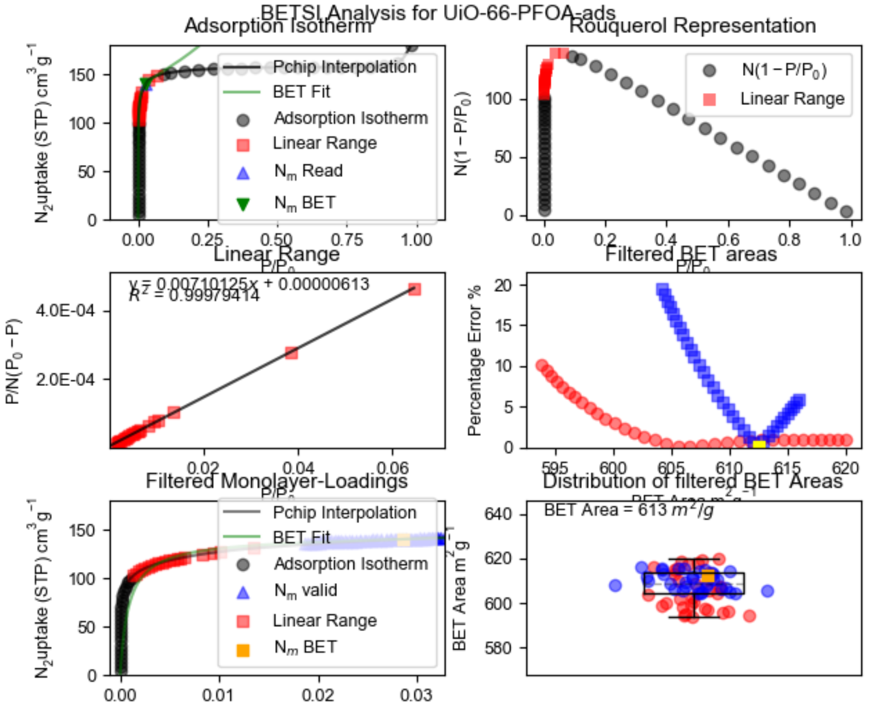

Figure S39. BETSI fitting and BET area calculations for **UiO-66-PFOA**.

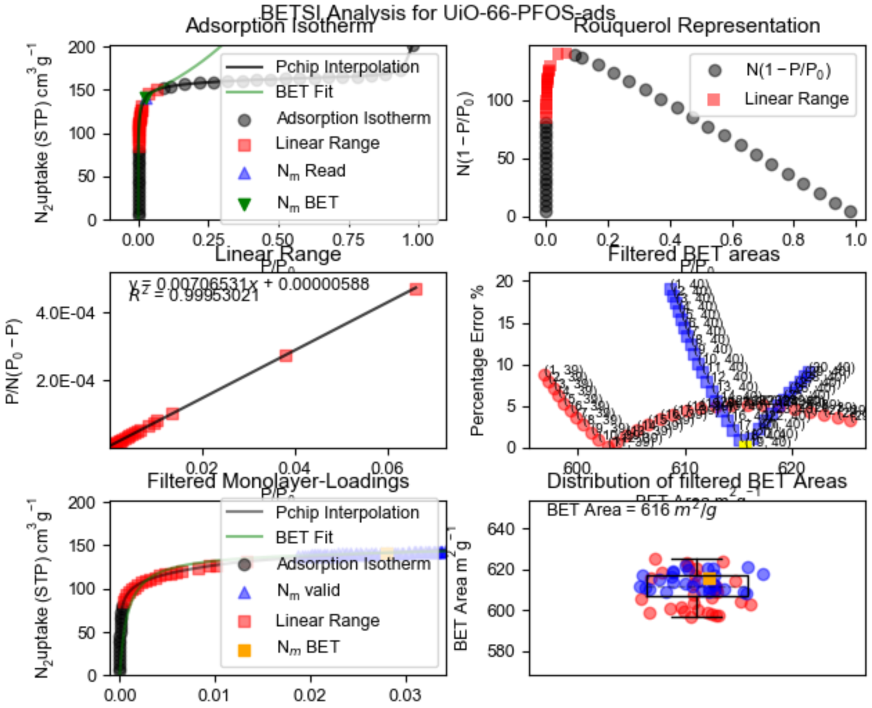

Figure S40. BETSI fitting and BET area calculations for **UiO-66-PFOS**.

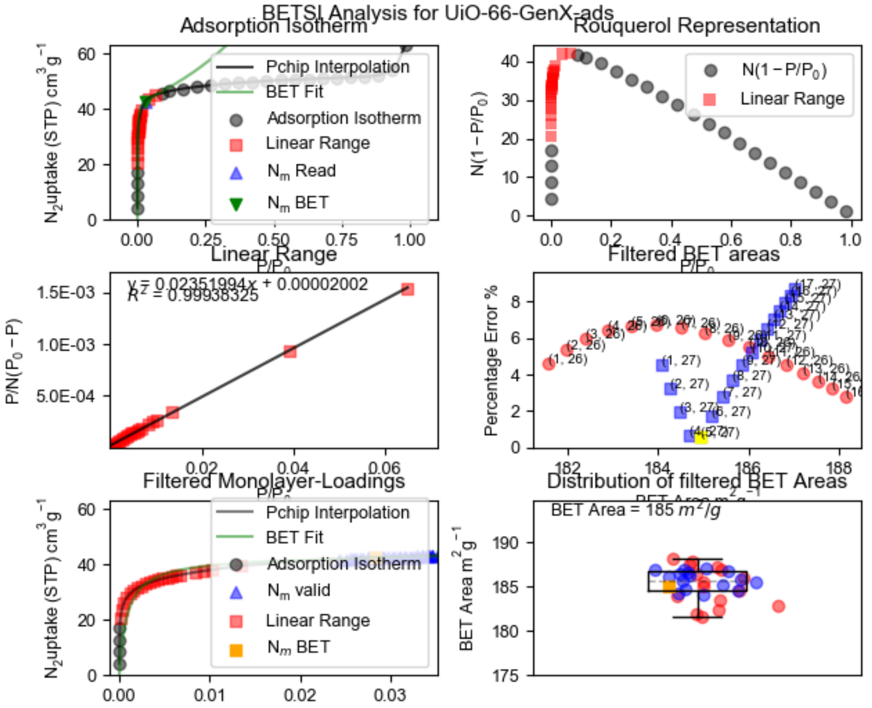

Figure S41. BETSI fitting and BET area calculations for **UiO-66-GenX**.

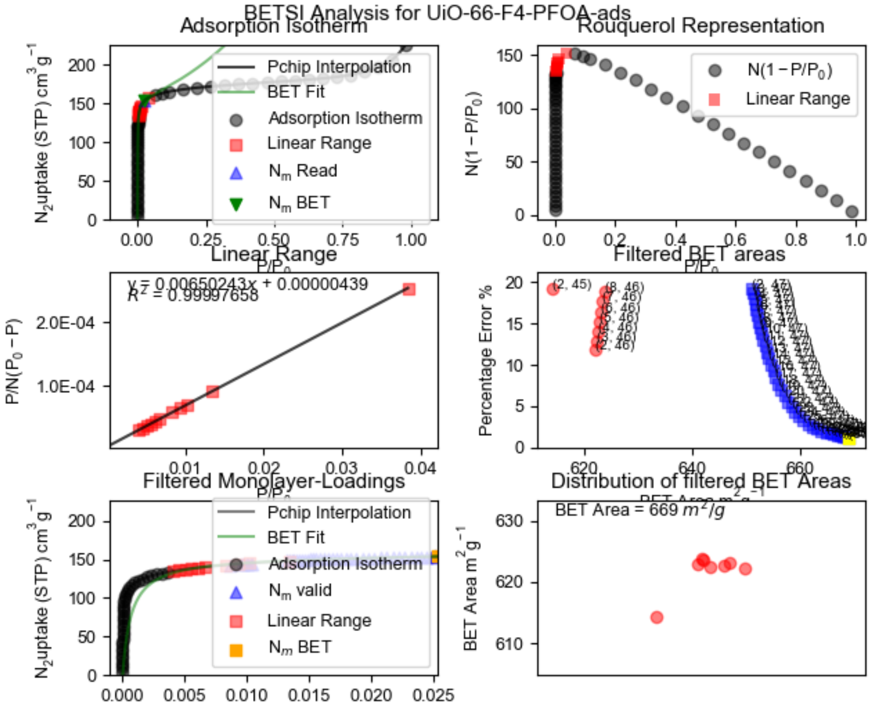

Figure S42. BETSI fitting and BET area calculations for **UiO-66-(F)$_4$-PFOA**.

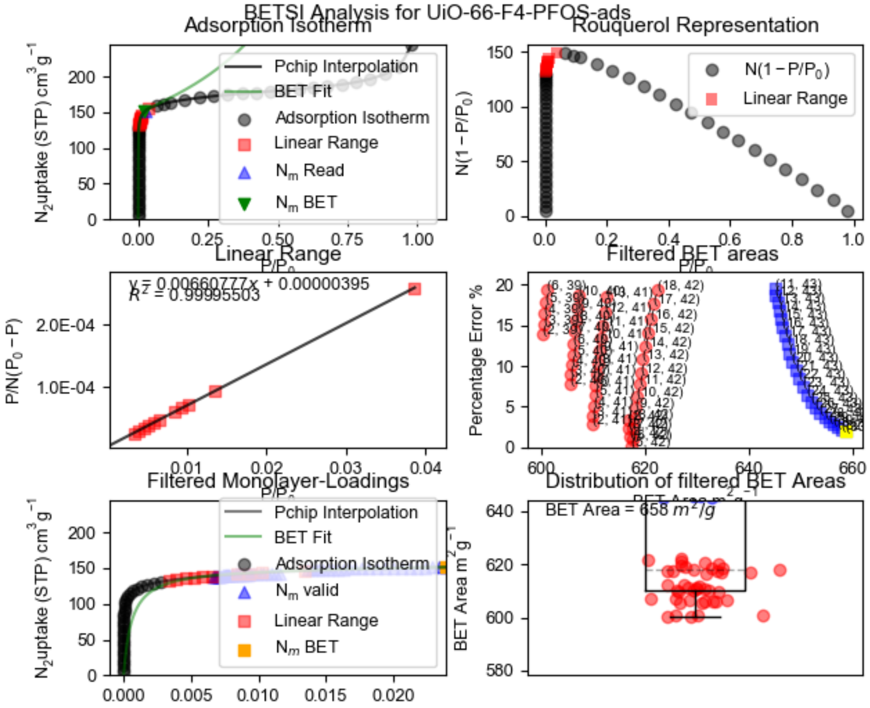

Figure S43. BETSI fitting and BET area calculations for **UiO-66-(F)$_4$-PFOS**.

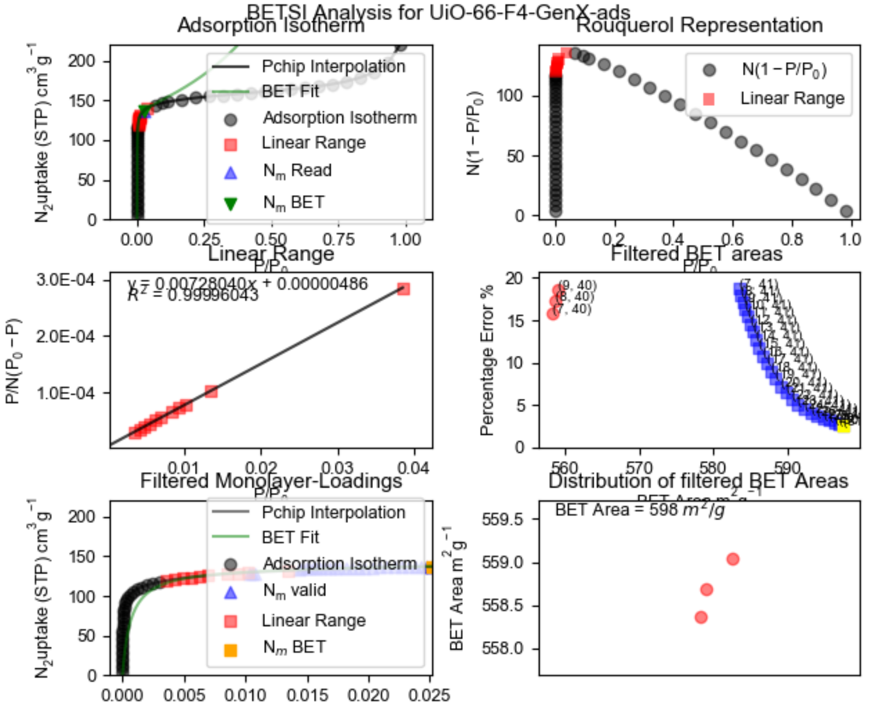

Figure S44. BETSI fitting and BET area calculations for **UiO-66-(F)$_4$-GenX**.

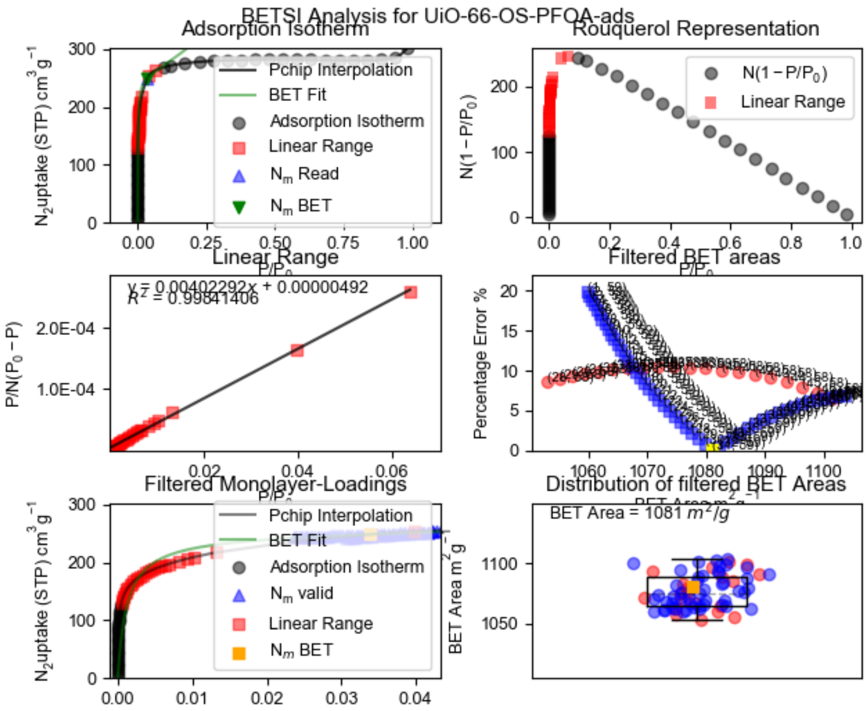

Figure S45. BETSI fitting and BET area calculations for **UiO-66-OS-PFOA**.

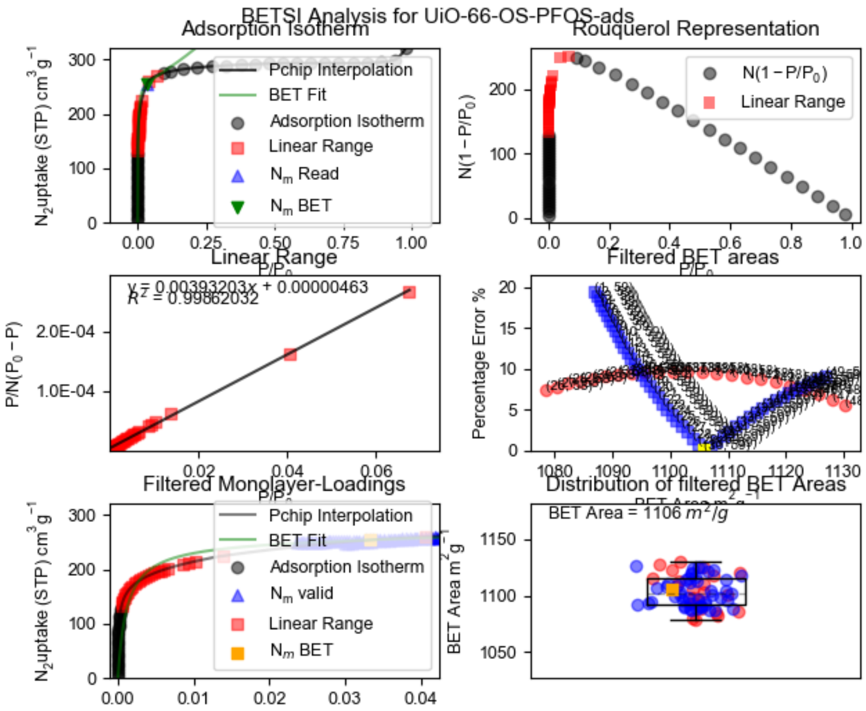

Figure S46. BETSI fitting and BET area calculations for **UiO-66-OS-PFOS**.

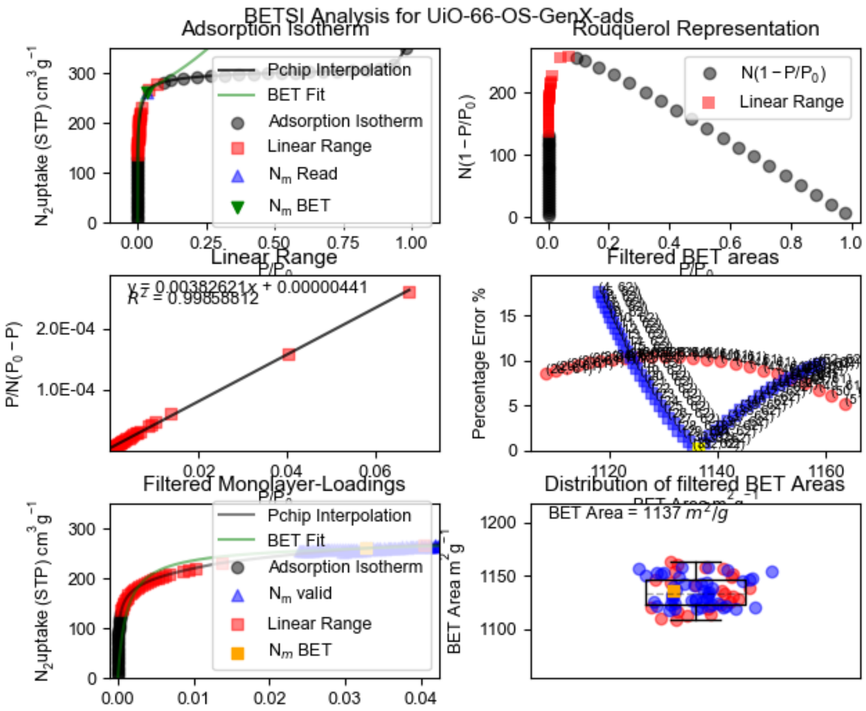

Figure S47. BETSI fitting and BET area calculations for **UiO-66-OS-GenX**.

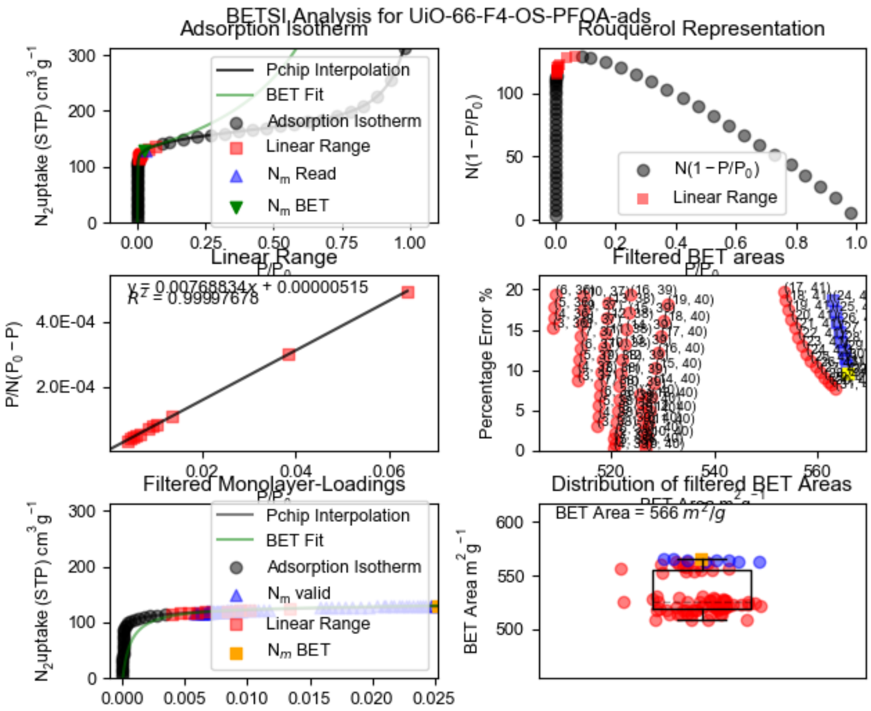

Figure S48. BETSI fitting and BET area calculations for **UiO-66-(F)$_4$-OS-PFOA**.

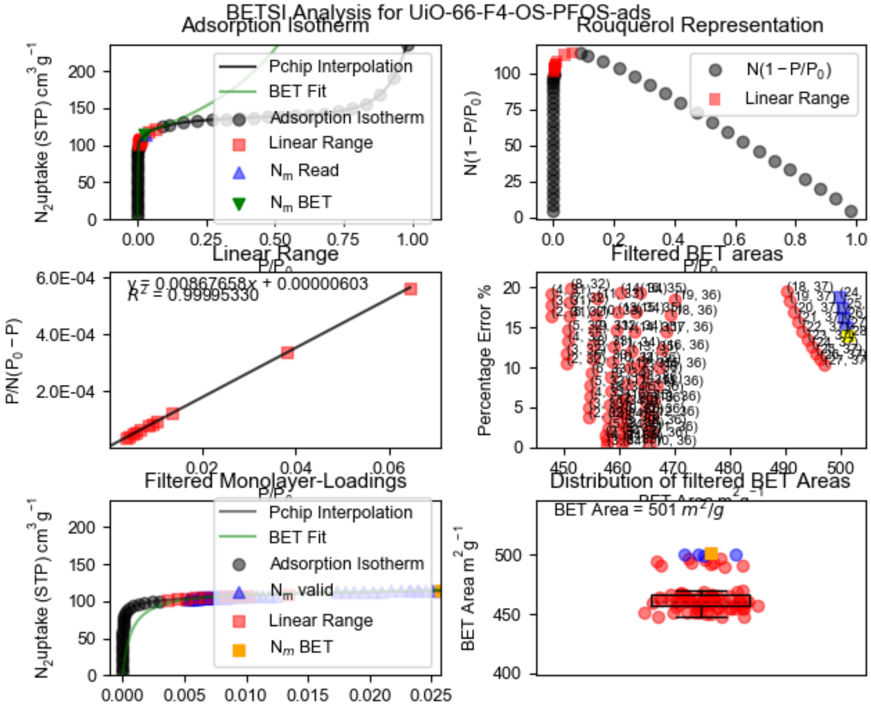

Figure S49. BETSI fitting and BET area calculations for **UiO-66-(F)$_4$-OS-PFOS**.

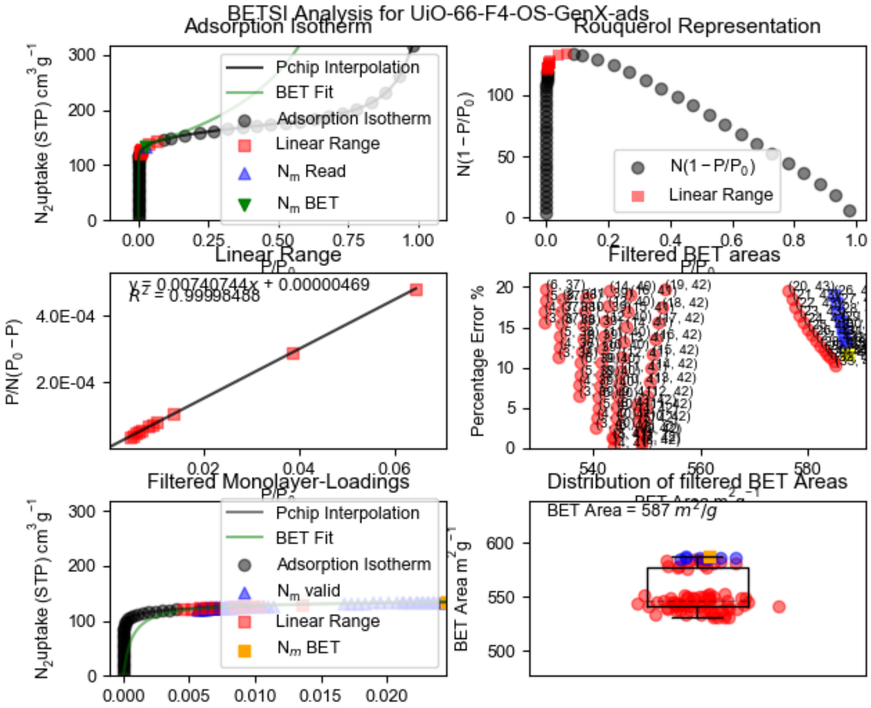

Figure S50. BETSI fitting and BET area calculations for **UiO-66-(F)$_4$-OS-GenX**.

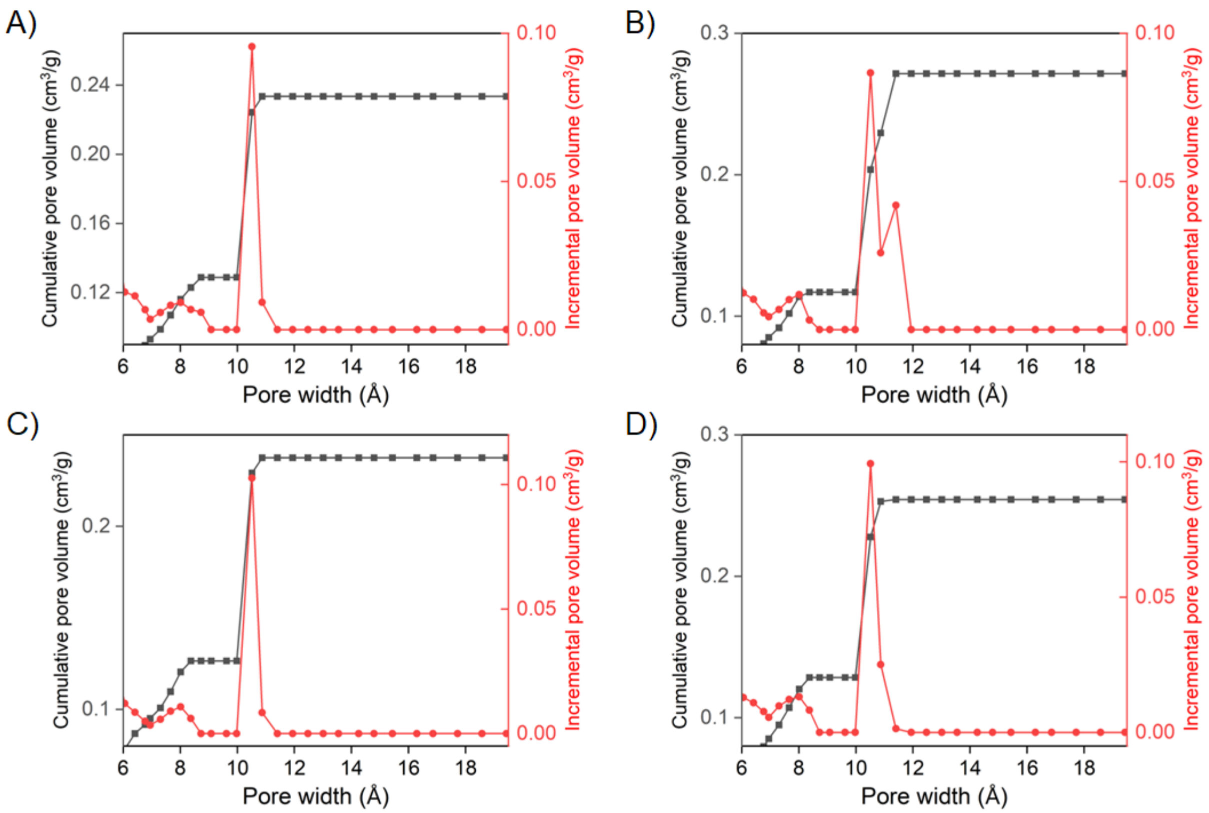

Figure S51. Pore size distribution profiles for A) **UiO-66-OS**; B) **UiO-66-OS@PFOA**; C) **UiO-66-OS@PFOS**; D) **UiO-66-OS@GenX** were obtained by fitting the NLDFT model to the respective 77 K N_2 adsorption branches.

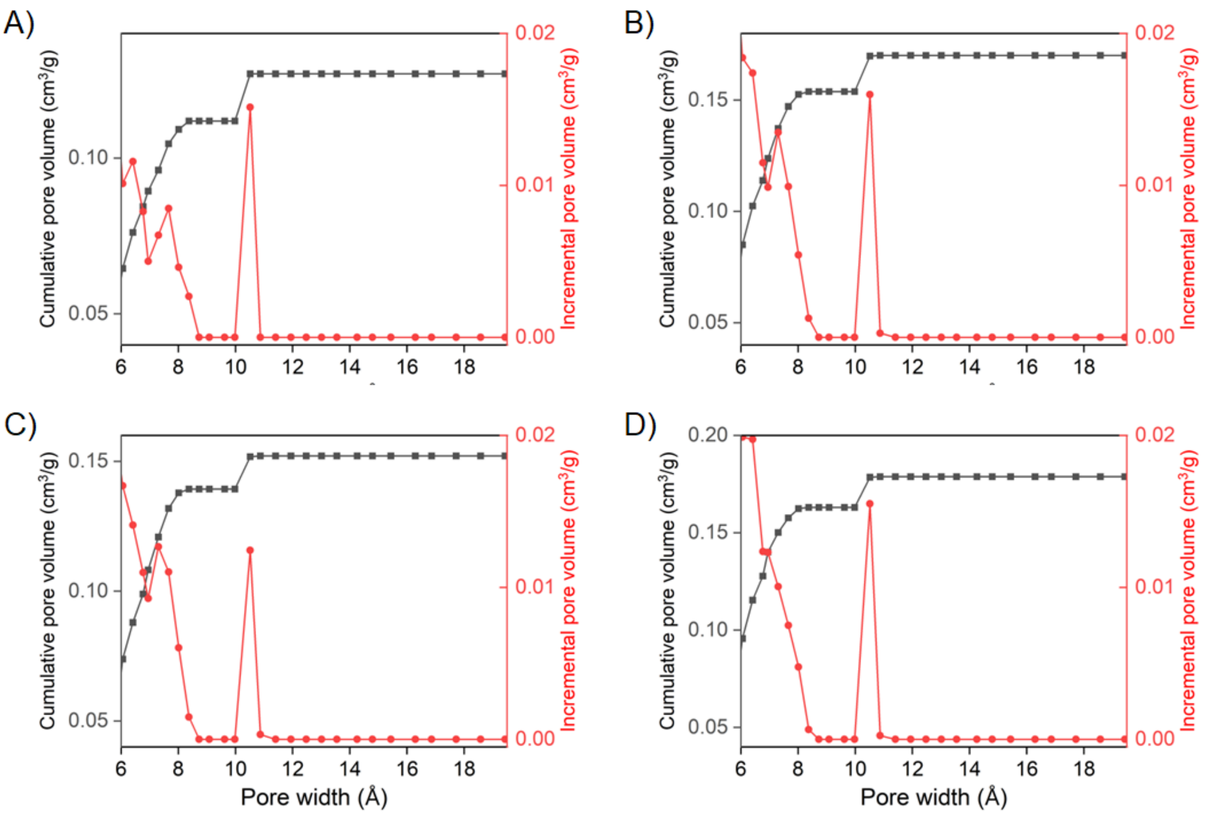

Figure S52. Pore size distribution profiles for A) **UiO-66-(F)$_4$-OS**; B) **UiO-66-(F)$_4$-OS@PFOA**; C) **UiO-66-(F)$_4$-OS@PFOS**; D) **UiO-66-(F)$_4$-OS@GenX** were obtained by fitting the NLDFT model to the respective 77 K N_2 adsorption branches.

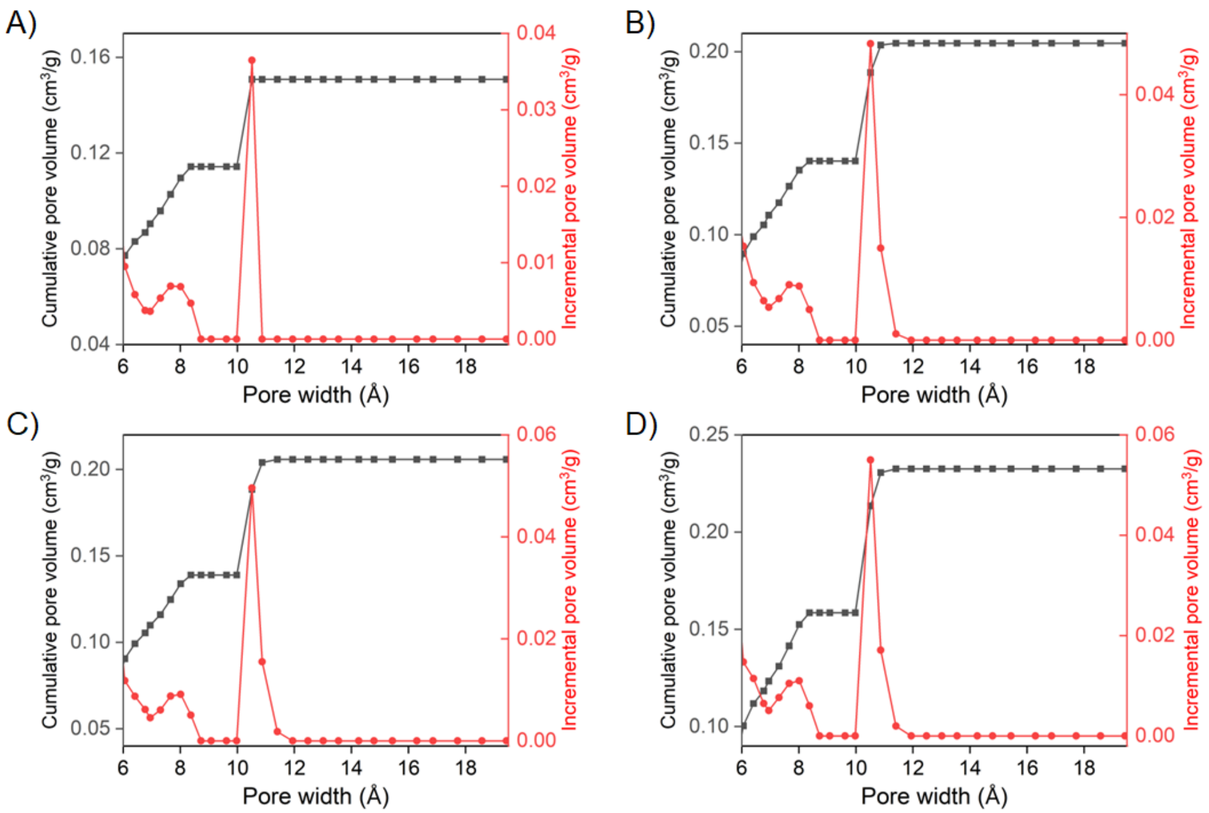

Figure S53. Pore size distribution profiles for A) **UiO-66-NO_2-OS**; B) **UiO-66-NO_2-OS@PFOA**; C) **UiO-66-NO_2-OS@PFOS**; D) **UiO-66-NO_2-OS@GenX** were obtained by fitting the NLDFT model to the respective 77 K N_2 adsorption branches.

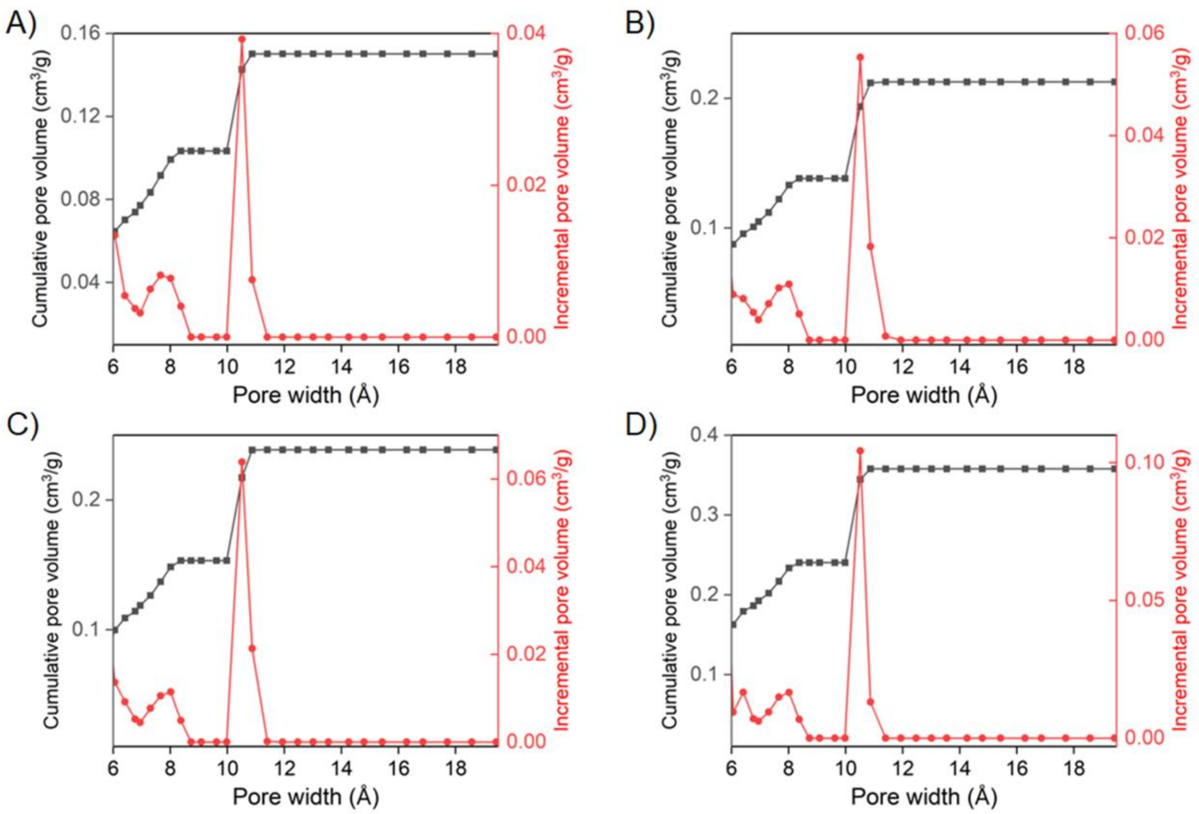

Figure S54. Pore size distribution profiles for A) **UiO-66-NH$_2$-OS**; B) **UiO-66-NH$_2$-OS@PFOA**; C) **UiO-66-NH$_2$-OS@PFOS**; D) **UiO-66-NH$_2$-OS@GenX** were obtained by fitting the NLDFT model to the respective 77 K N_2 adsorption branches.

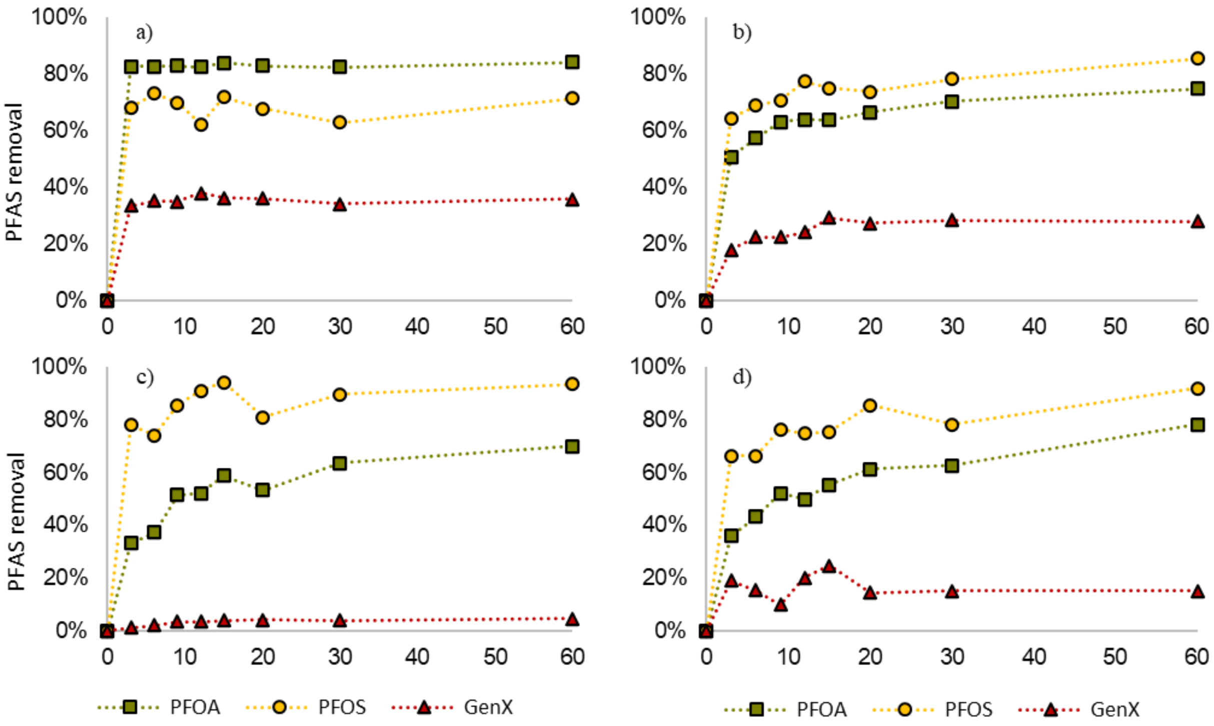

Figure S55. First 60 minutes of adsorption kinetics for PFOA, PFOS and GenX, for a) **UiO-66**, b) **UiO-66-OS**, c) **UiO-66-(F)$_4$**, and d) **UiO-66-(F)$_4$-OS**. The analysis was conducted from the same samples, where 20 mg of MOF in 50 mL of PFAS sample was agitated in a water bath shaker, stopping the shaker for sampling.

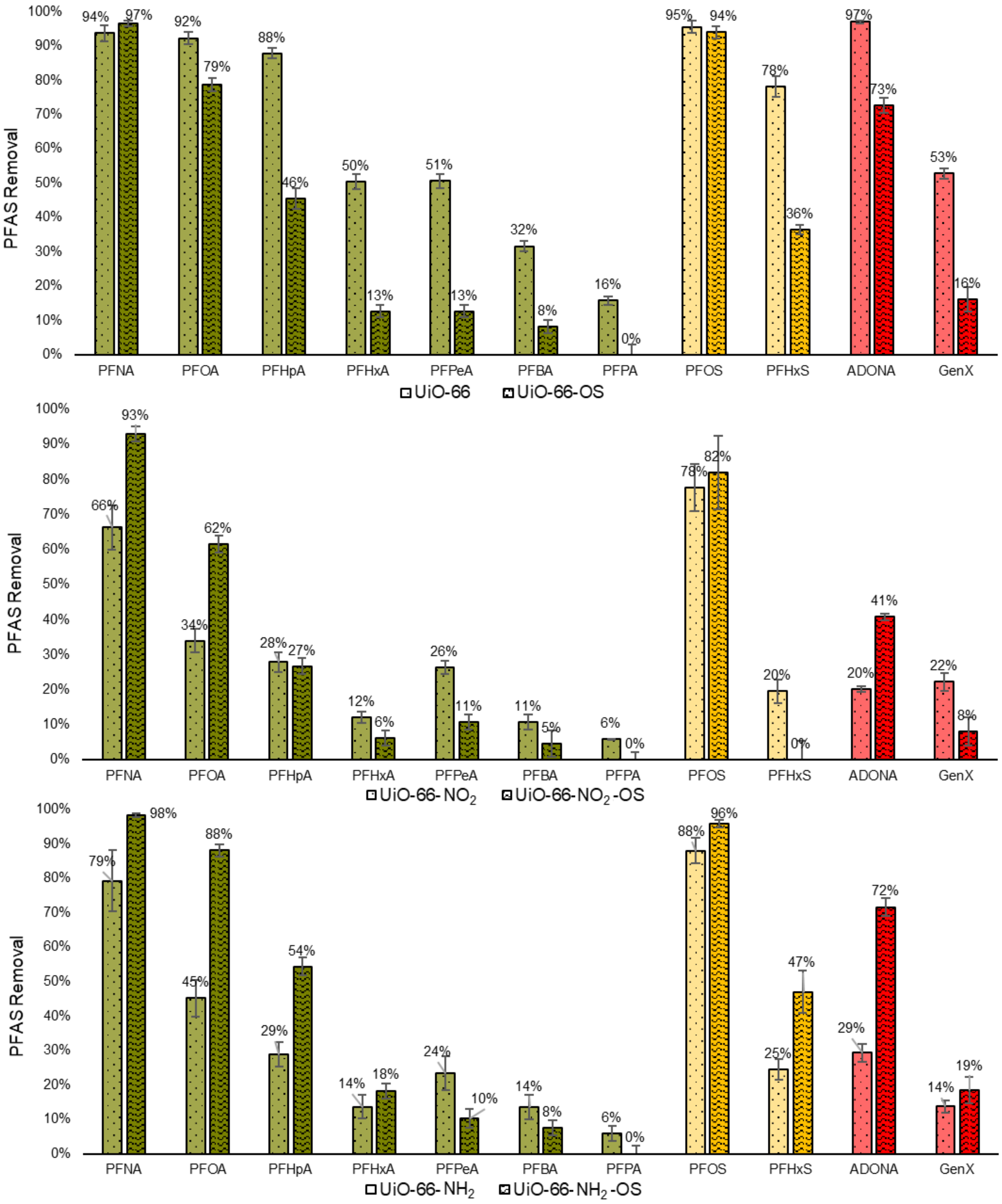

Figure S56. Comparison of adsorption performance between pristine **UiO-66**, **UiO-66-NO₂**, and **UiO-66-NH₂**, and their OS analogues at trace PFAS concentration levels. The 11 PFAS were spiked as a mixture for a starting concentration of ~2 μg L^{-1} for each, and the vials shaken for 48 h in a water bath.

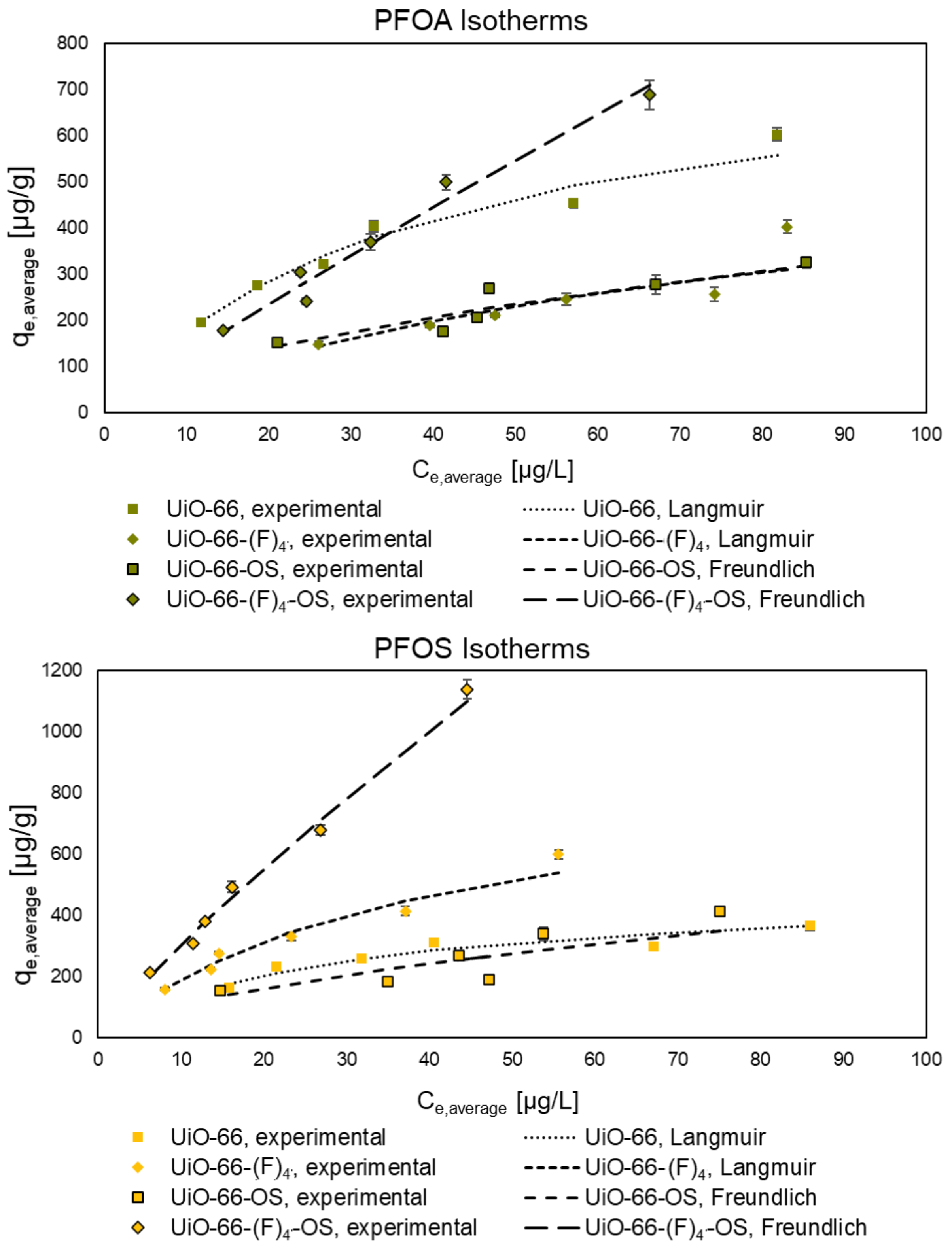

Figure S57. PFOA and PFOS adsorption isotherms for the best-performing sorbents: **UiO-66**, **UiO-66-(F)4**, **UiO-66-OS**, and **UiO-66-(F)4-OS**. Isotherm parameters obtained from linear regression were used to plot isotherm models presented in the graphs.

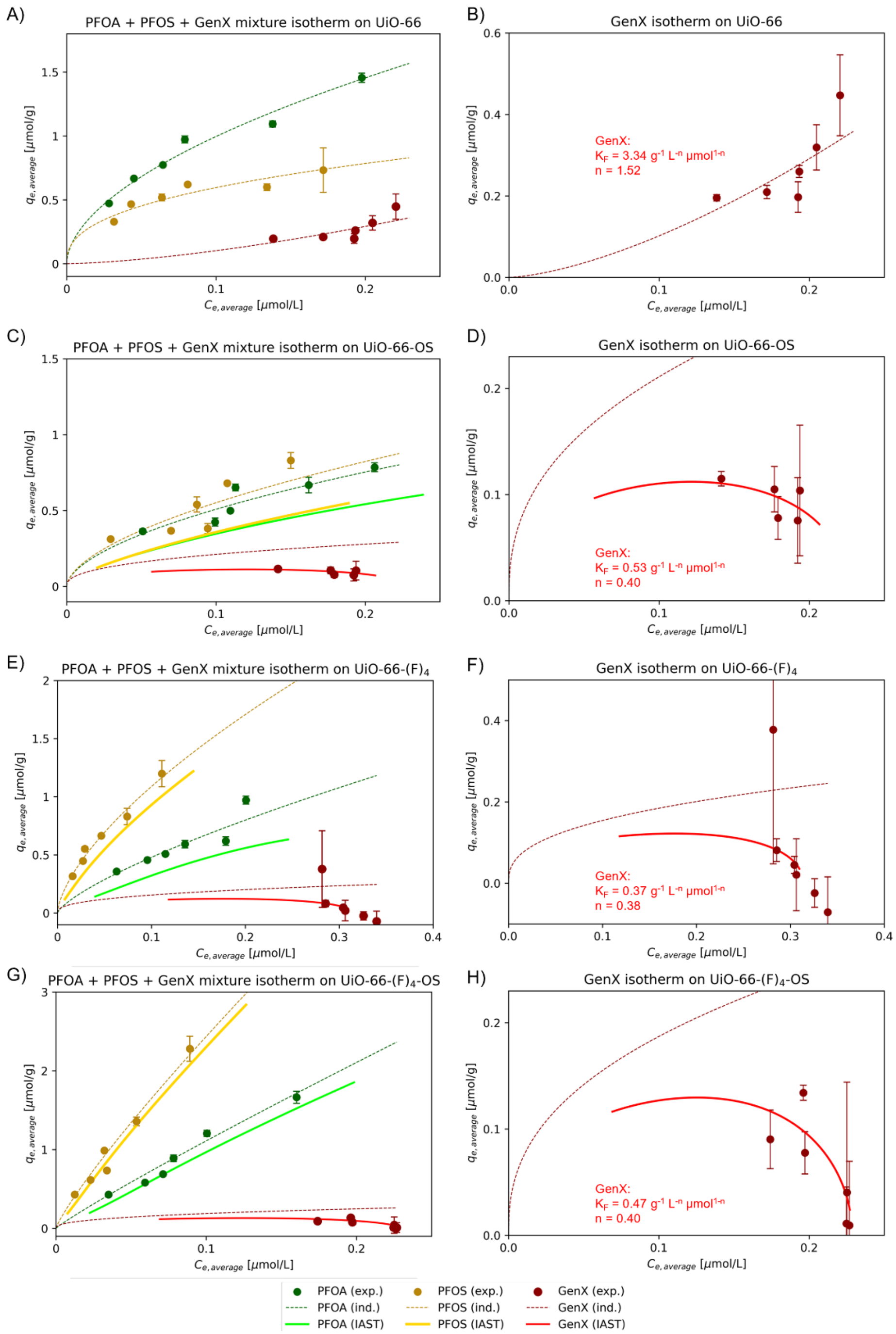

Figure S58. PFAS mixture isotherms on **UiO-66**, **UiO-66-OS**, **UiO-66-(F)4** and **UiO-66-(F)4-OS**. The competitive adsorption behaviour was modelled based on IAST to evidence the displacement of GenX. Panel B, D, F and H are respective close-ups of the panels A, C, E and G, showing GenX data only. Scatterplot – experimental results (averages with standard deviations), dashed lines-- isotherm based on single-solute assumption (individual, ind., *cf*. Table 1, manuscript), and solid lines – competitive adsorption model based on IAST with estimated GenX isotherm parameters. For **UiO-66-OS**, **UiO-66-(F)4** and **UiO-66-(F)4-OS**, the isotherms demonstrate competitive adsorption behaviour. Notably, GenX is displaced by the two other better adsorbing PFAS.

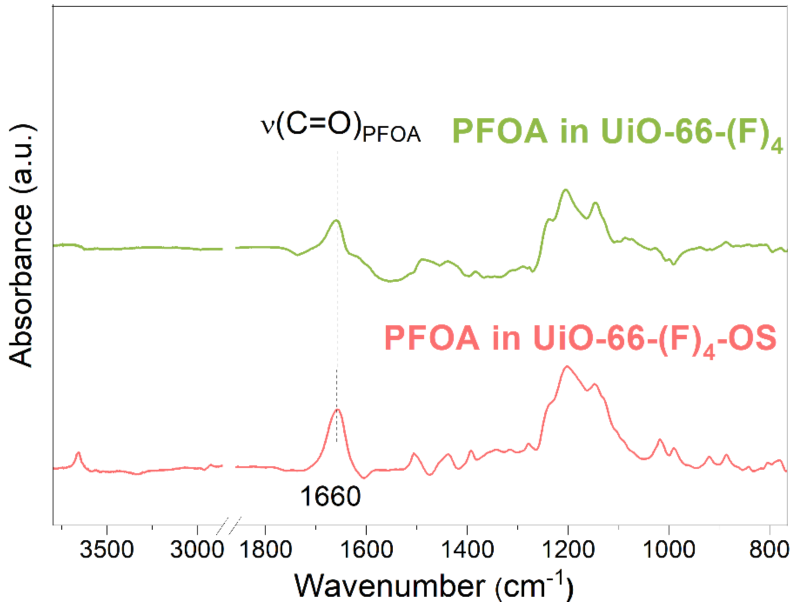

Figure S59. Difference FT-IR spectra showing the modes of adsorbed PFOA in **UiO-66-(F)4** obtained by subtracting the spectrum of pristine **UiO-66-(F)4** from that of the PFOA-loaded one (bottom) (see the bottom panel in Figure 3); in **UiO-66-(F)4-OS** obtained by subtracting the spectrum of pristine **UiO-66-(F)4-OS** from that of the PFOA-loaded one (bottom) (see the bottom panel in Figure 3).

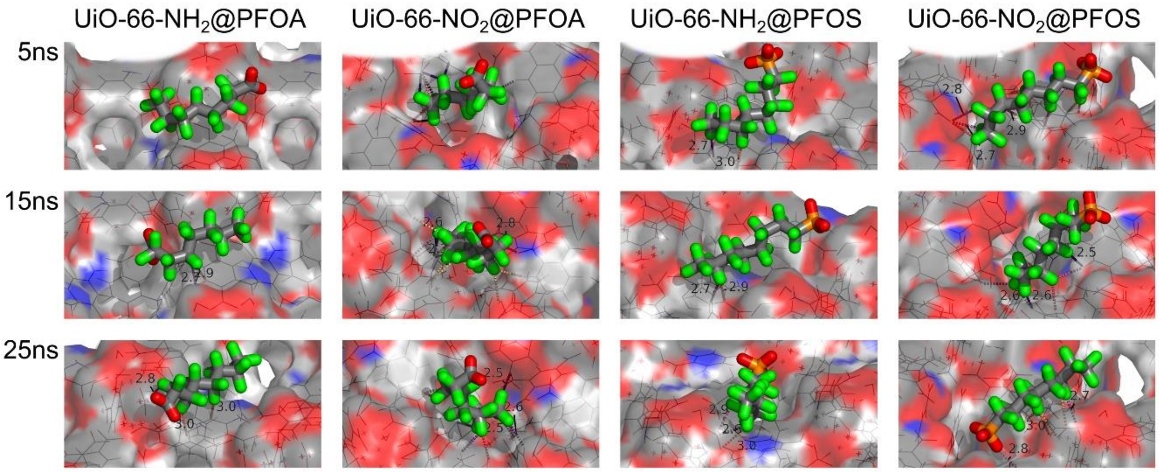

Figure S60. MD snapshots of sample trajectories for the MOFs, **UiO-66-NH2** and **UiO-66-NO2**. Marked up are the inter-atom-distances < 0.3 nm. The three shortest distances are annotated, in Å.

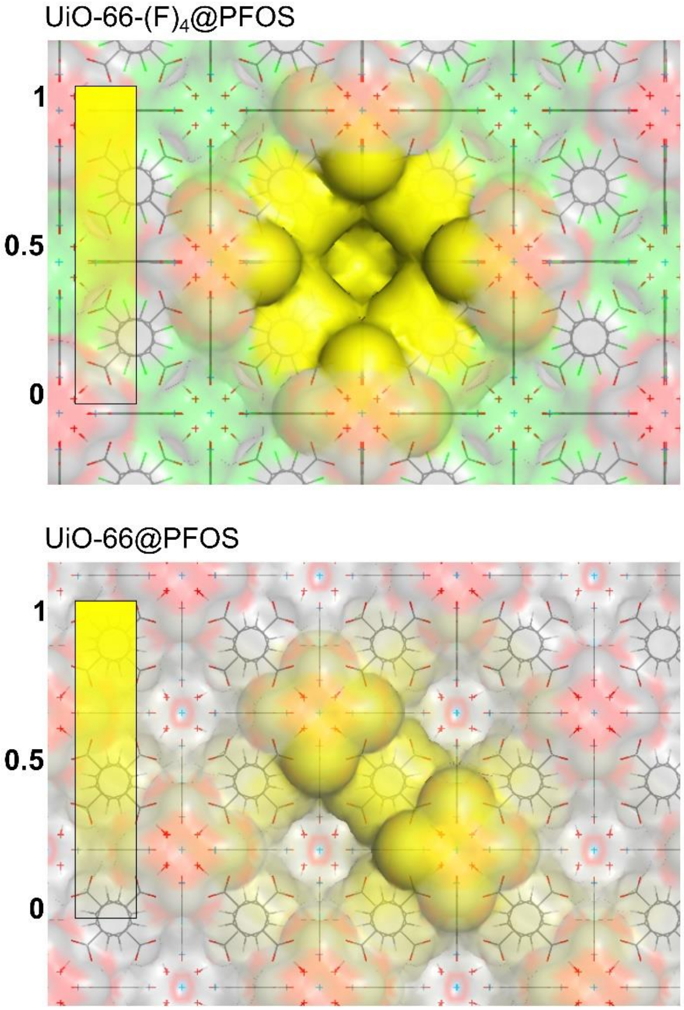

Figure S61. Contact ratio maps for **UiO-66@PFOS** and **UiO-66-(F)$_4$@PFOS**.

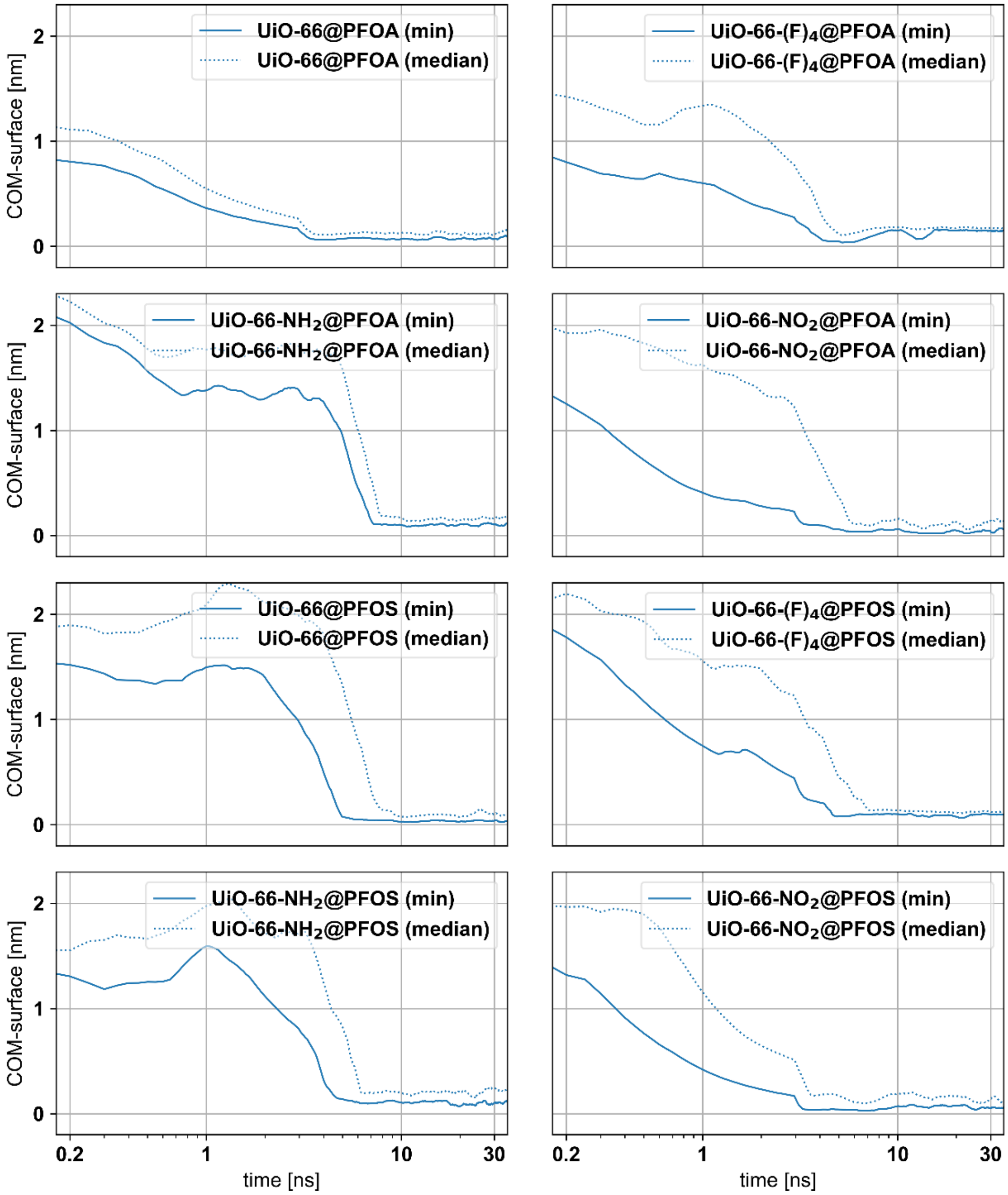

Figure S62. Distances between the PFAS-COM and MOF surface along simulation time, visualised across all simulation runs (at least 5 × 35 ns for the **UiO-66/UiO-66-(F)$_4$**, 3 × 35 ns for **UiO-66-NH$_2$** and **UiO-66-NO$_2$**). Plotted is a dynamic average of the closest minimal distance across trajectories (solid line) as well as the median distance (dotted line).

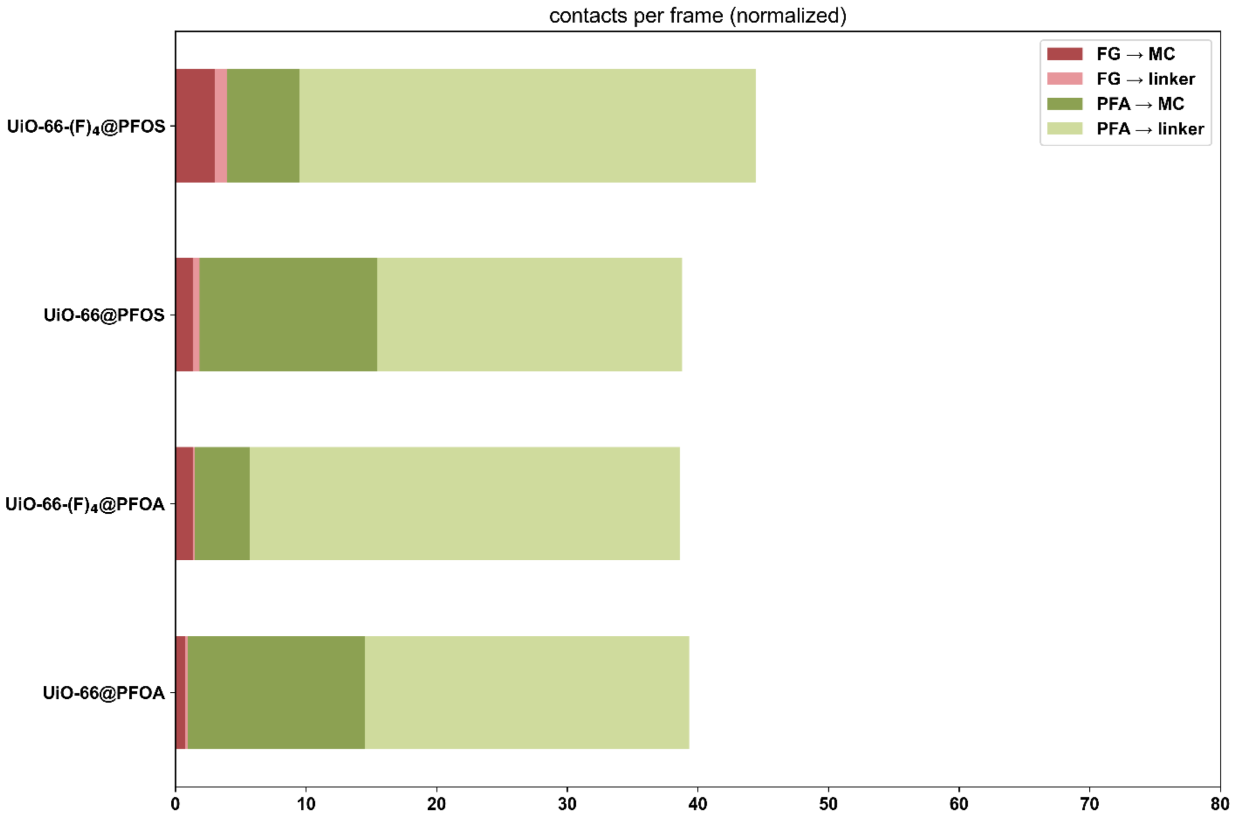

Figure S63. Contacts < 0.4 nm between the MOF and the PFAS molecules, normalised across the number of simulation snapshots considered. Contacts are differentiated by the type of subunits of the MOF (linker/metal cluster) and PFAS (backbone/functional group)-system to which the contact-pair-atoms belong. Shown are the trends regarding how the **UiO-66** / **UiO-66-(F)$_4$** MOFs behave, a comparison with the NX_2-functionalised systems would need to consider the fact that the number of possible contact-sites is significantly different within the cavity of the functionalised MOF. To clarify that the numbers are not directly comparable, we have included this data in Figure S64. Please also note that PFOA and PFOS have different heavy-atom backbone lengths.

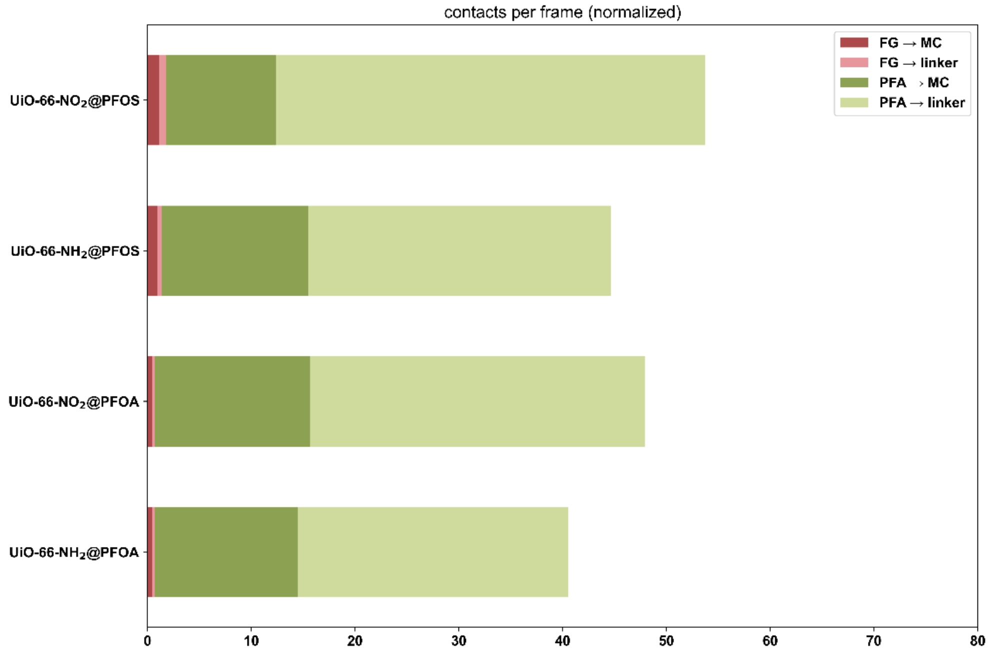

Figure S64. Contacts < 0.4 nm between the MOF and the PFAS molecules, normalised across the number of simulation snapshots considered. Contacts are differentiated by the type of subunits of the MOF (linker/metal cluster) and PFAS (backbone/functional group)-system to which the contact-pair-atoms belong. Shown are the trends regarding **UiO-66-NH$_2$ / UiO-66-NO$_2$**.

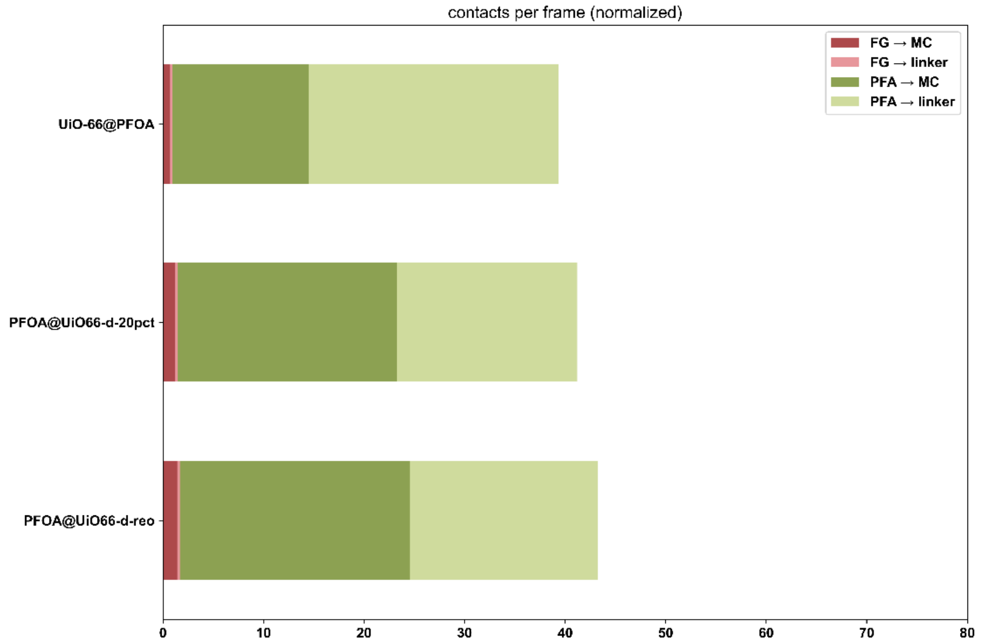

Figure S65. Contacts < 0.4 nm between different (defective) realisations of **UiO-66** and the PFAS molecules, normalised across the number of simulation snapshots considered. Contacts are differentiated by the type of subunits of the MOF (linker/metal cluster) and PFAS (backbone/functional group)-system to which the contact-pair-atoms belong. We see a marked increase in PFAS-MCA contacts when introducing defects.

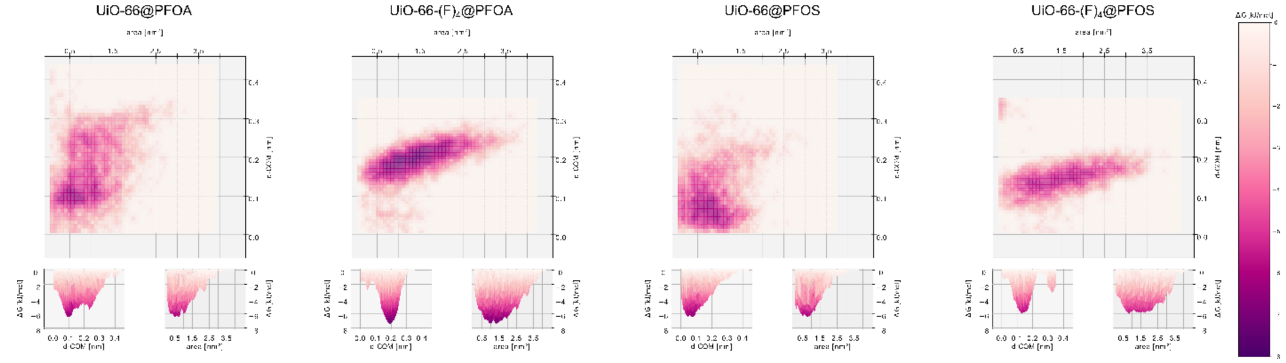

Figure S66. Probability plots for **UiO-66@PFAS** and **UiO-66-(F)4@PFAS**, in a specific (distance *vs.* contact-area) state expressed in the units of free energy. The data was recorded across all simulation runs for the respective **MOF@PFAS** combination in timesteps of 50 ns. Distances are recorded following the distances of the PFOA molecule's COM from the topmost MOF atoms, measured along the surface normal. Contact areas were determined with a modified solvent-accessible surface area algorithm (see the methods section). These graphs augment Fig 4D, where only PFOA was shown. The shape of the probability-distributions does not differ between the different PFAS, but shows strong differences between the different MOF sorbents.

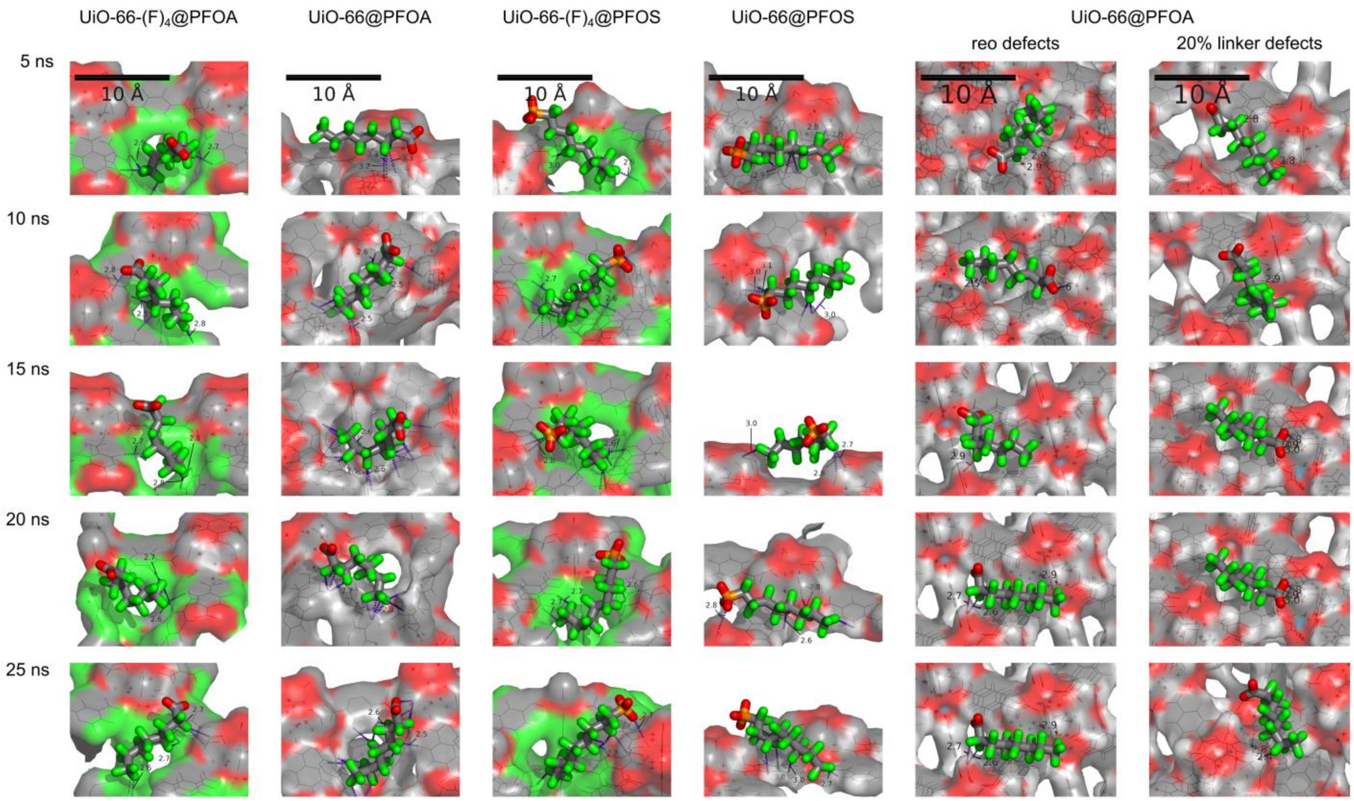

Figure S67. Molecular dynamics snapshots for the two best-performing MOF sorbents, **UiO-66** and **UiO-66-(F)4** (left), and for these two **UiO-66** structures including structural defects (right); each taken from a single MD run. Marked up are inter-atomic distances < 0.3 nm. The three shortest distances are annotated in Å.

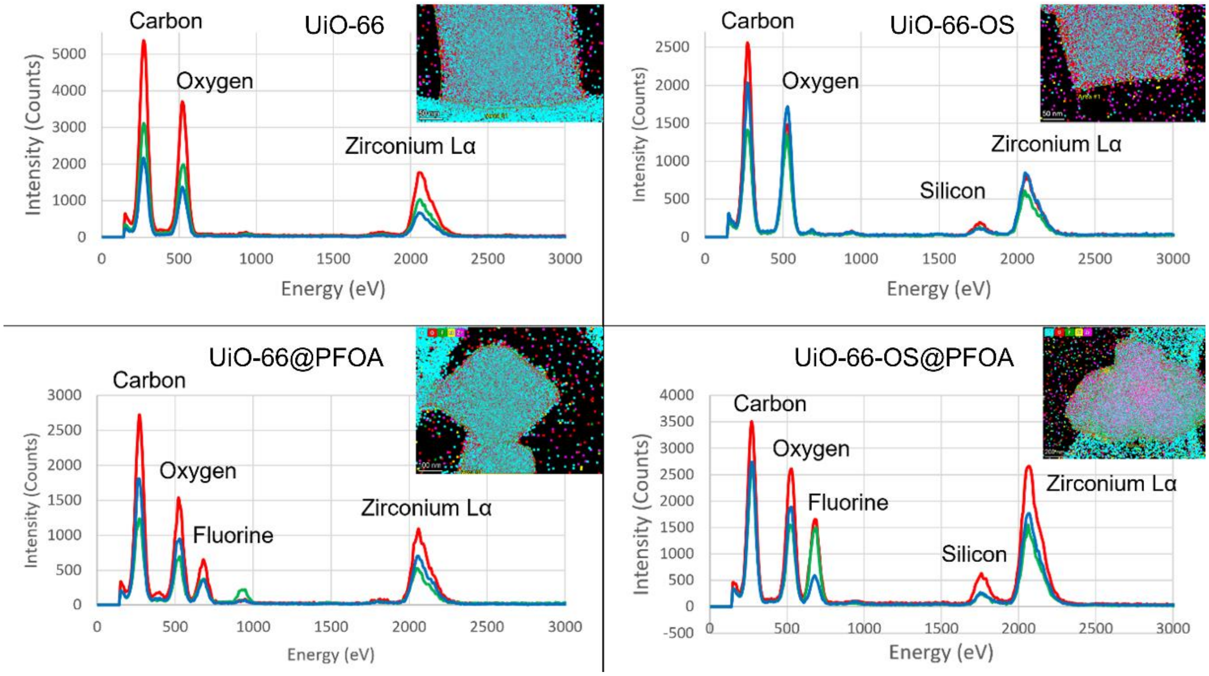

Figure S68. EDX spectra displayed for **UiO-66**, **UiO-66-OS**, **UiO-66@PFOA**, and **UiO-66-OS@PFOA** (corresponding to the sample labels) with a representative EDX map image inset where all elemental components are represented, *i.e.*, C (cyan), O (red), F (green), Si (yellow), and Zr (pink).

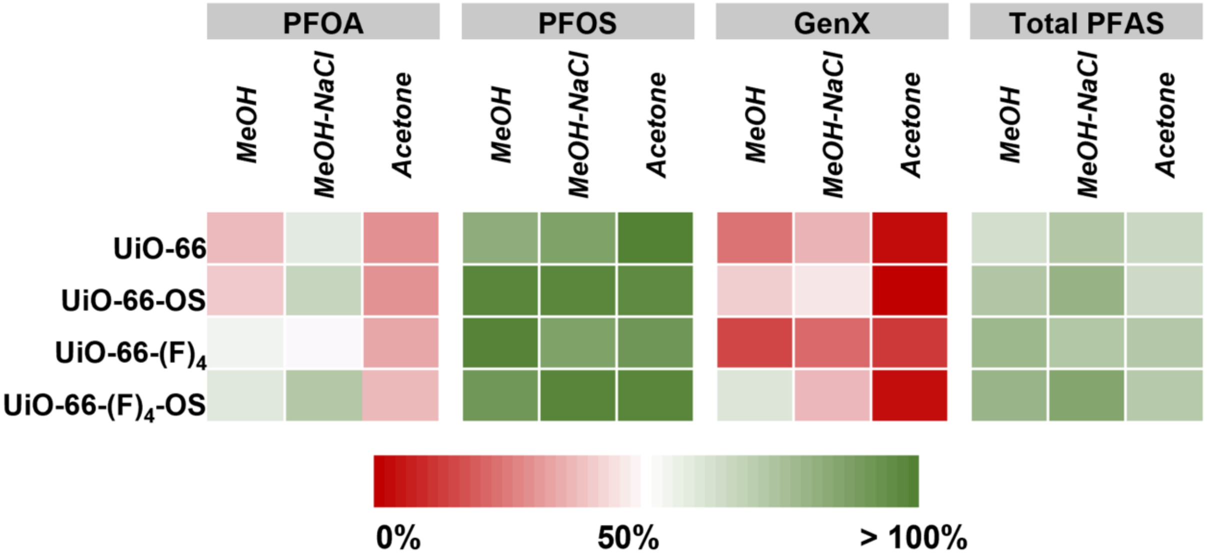

Figure S69. Preliminary regeneration performance analysis for the selected 4 UiO-66-X sorbents with three different solvents (MeOH, MeOH with NaCl, and acetone), colour coded for easier identification of the best-performing solvent to be used in the full regeneration study. Regeneration performance is assessed by initially determining the quantity of adsorbed PFAS, followed by quantifying PFAS in the regenerant (*i.e.*, the percentage of the adsorbed PFAS that was recovered), as explained previously with Equations 1-4 (Supporting Information).

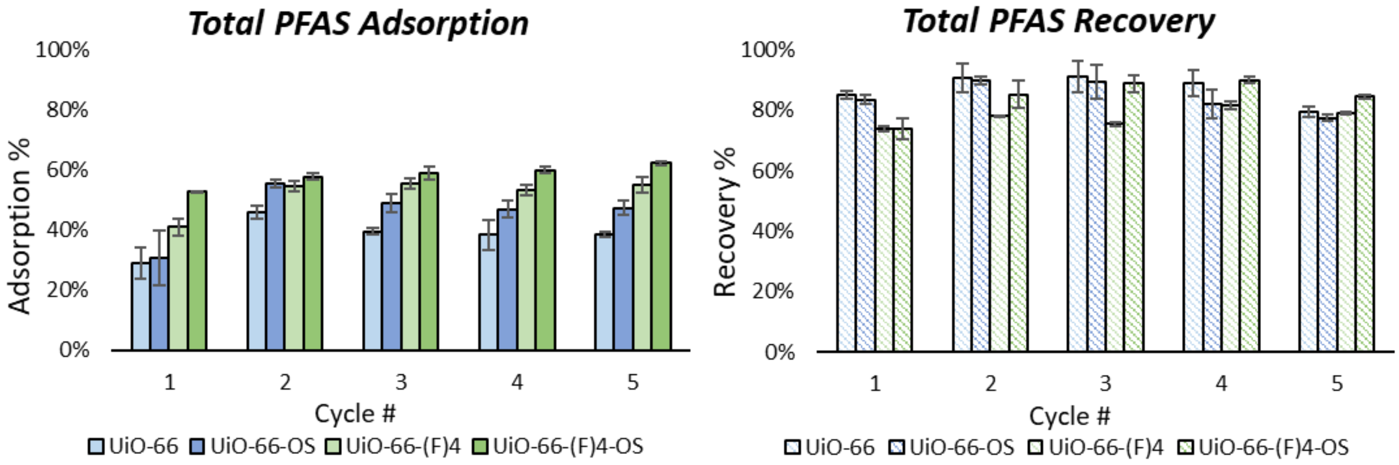

Figure S70. Total PFAS adsorption and regeneration performance overview for the 4 tested sorbents: **UiO-66**, **UiO-66-(F)$_4$**, **UiO-66-OS**, and **UiO-66-(F)$_4$-OS**, over the course of 5 cycles. Adsorption represents the percentage of the total PFAS as measured against the blank control that were removed in each cycle, while recovery displays the percentage of the removed PFAS that was subsequently recovered with the regeneration solvent.

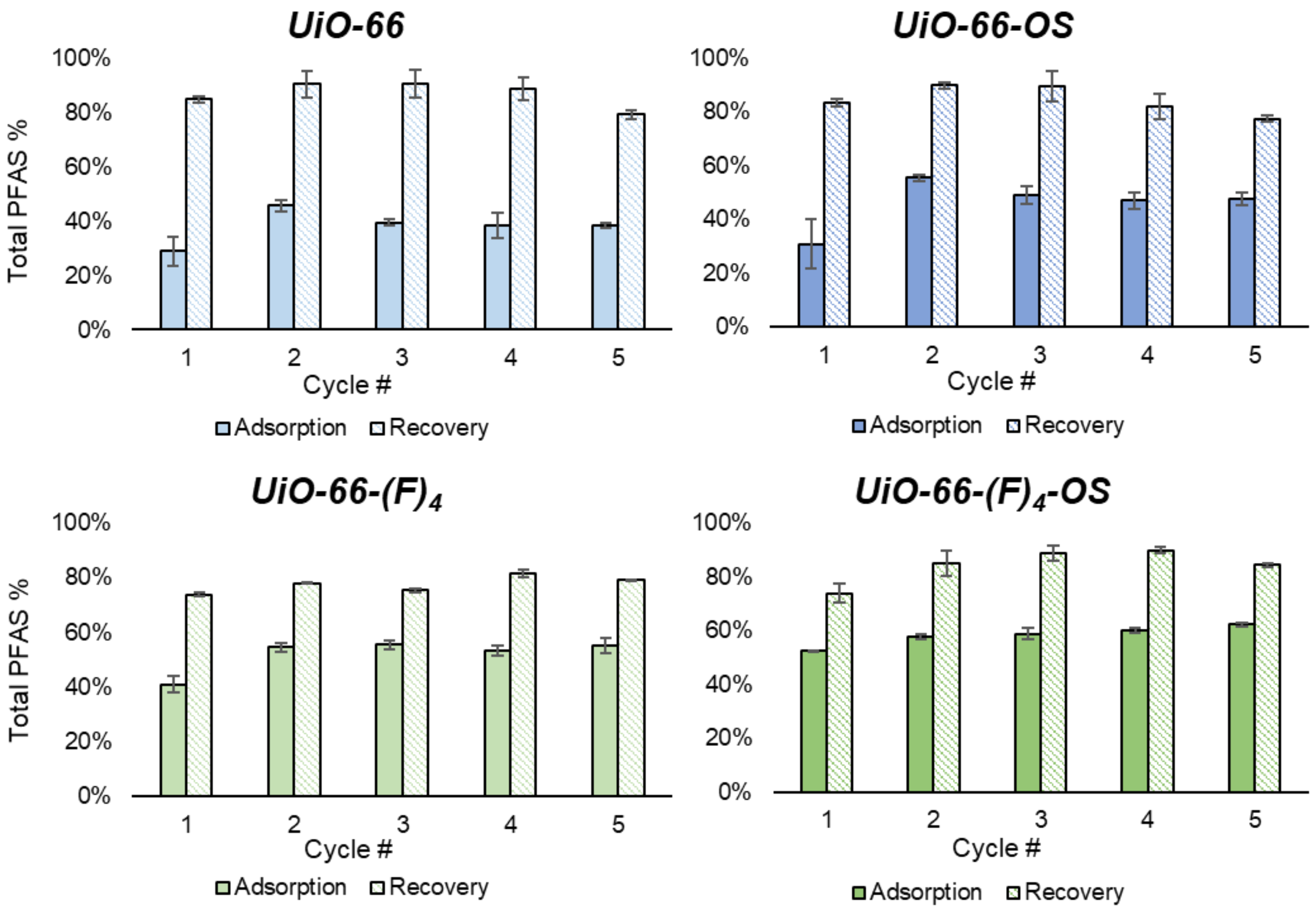

Figure S71. Total PFAS adsorption and regeneration performance of the 4 tested sorbents: **UiO-66**, **UiO-66-(F)$_4$**, **UiO-66-OS**, and **UiO-66-(F)$_4$-OS** (in comparison and individually), over the course of 5 cycles. Adsorption represents the percentage of the total PFAS as measured against the blank control that were removed in each cycle, while recovery displays the percentage of the removed PFAS that was subsequently recovered with the regeneration solvent.

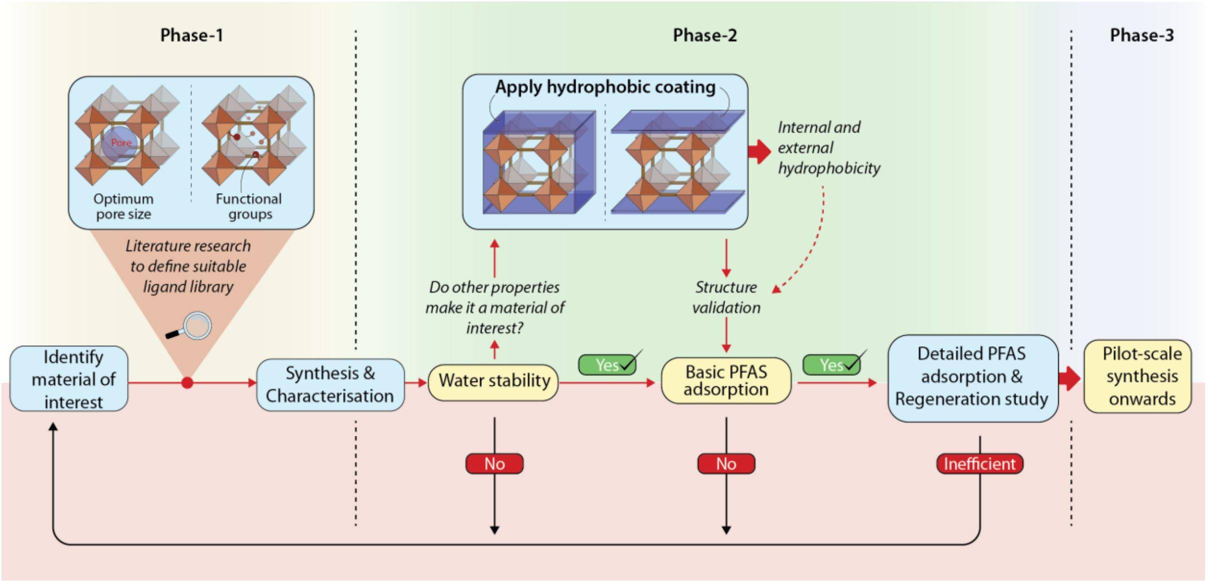

Figure S72. Generalised, bottom-up sorbent design paradigm: Rather counterintuitively, albeit limited guest-accessible porosity, the prepared polymer-MOF composites showcase high trace (ppb-level) PFAS removal performances. This study offers insights into which kind of MOF functionalisation routes (pore *versus* surface) elicit optimal surface signatures, just right to exhibit strong affinity to PFAS contaminants (even when present in trace concentrations).

Supporting Information Tables.

Table S1. Full list of PFAS chemicals used in this study.

PFAS	Full name	Manufacturer	PFAS type	Concentration
**PFOA**	Perfluoro-*n*-[$^{13}C_8$] octanoic acid	Campro-Scientific	^{13}C mass-labelled	50 μg mL^{-1}
**PFOS**	Perfluoro-*n*-[$^{13}C_8$]octanesulfonate	Campro-Scientific	^{13}C mass-labelled	50 μg mL^{-1}
**GenX (HFPO-DA)**	Hexafluoro-[$^{13}C_3$]propylene Oxide Dimer Acid	Campro-Scientific	^{13}C mass-labelled	50 μg mL^{-1}
**PFNA**	Perfluorononanoic acid	Thermo-fisher Scientific	Reagent grade	Solid
**PFOA**	Perfluorooctanoic acid	Thermo-fisher Scientific	Analytical grade	100 μg mL^{-1}
**PFOA**	Perfluorooctanoic acid	Thermo-fisher Scientific	Reagent grade	Solid
**PFHpA**	Perfluoroheptanoic acid	Thermo-fisher Scientific	Reagent grade	Solid
**PFHxA**	Perfluorohexanoic acid	Thermo-fisher Scientific	Reagent grade	Liquid
**PFPeA**	Perfluoropentanoic acid	Thermo-fisher Scientific	Reagent grade	Liquid

**PFBA**	Perfluorobutanoic acid	Thermo-fisher Scientific	Reagent grade	Liquid
**PFPA**	Perfluoropropionic acid	Thermo-fisher Scientific	Reagent grade	Liquid
**PFOS**	Perfluorooctane sulfonate	Thermo-fisher Scientific	Analytical grade	100 μg mL^{-1}
**PFOS**	Perfluorooctane sulfonate	Thermo-fisher Scientific	Reagent grade	Solid
**PFHxS**	Perfluorohexane sulfonate	Thermo-fisher Scientific	Reagent grade	Solid
**GenX (HFPO-DA)**	Hexafluoropropylene Oxide Dimer Acid	Campro-Scientific	Analytical grade	50 μg mL^{-1}
**GenX (HFPO-DA)**	Hexafluoropropylene Oxide Dimer Acid	Toronto Research Chemicals	Reagent grade	Liquid
**NaDONA**	Sodiumdodecafluoro-3H-4,8-dioxanonanoate	Campro-Scientific	Analytical grade	50 μg mL^{-1}

Table S2. - Mass to charge ratio (m/z) of fragments used in quantification of each PFAS; two fragments were always analysed for quality control.

PFAS type	PFAS m/z ratio	^{13}C mass-labeled PFAS m/z ratio
**PFOA**	PFOA_169	M8PFOA_172
	PFOA_369	M8PFOA_376
**PFOS**	PFOS_80	M8PFOS_80
	PFOS_99	M8PFOS_99
**GenX (HFPO-DA)**	HFPO-DA_169	M3HFPO-DA_170
	HFPO-DA_285	M3HFPO-DA_287
**ADONA**	ADONA_85	-
	ADONA_377	-
**PFNA**	PFNA_219	-
	PFNA_419	-
**PFHpA**	PFHpA_169	-
	PFHpA_319	-
**PFHxA**	PFHxA_119	-
	PFHxA_269	-
**PFHxS**	PFHxS_80	-
	PFHxS_99	-
**PFPeA**	PFPeA_219	-
	PFPeA_263	-
**PFBA**	PFBA_169	-
	PFBA_213	-
**PFPA**	PFPA_119	-
	PFPA_163	-

Samples and standards were always spiked with freshly prepared internal standard mixture for quality assurance. Batch, kinetics, isotherm and regeneration experiments were all diluted to match the effective measurement range of the instrument, while the trace experiments were measured without dilution.

Table S3. Analysis of saturation uptakes (at ≈1 bar) and BET surface areas (SAs) for the post-coating sorbents, compared to the activated phases of **UiO-66** obtained pre-polymer coating (green rows), **UiO-66-(F)$_4$** (grey rows), **UiO-66-NO$_2$** (orange rows) and **UiO-66-NH$_2$** (blue rows).

Sorbent	1 bar uptake N_2 (mmol g^{-1})	BET SA	% reductions in BET SA	% reductions in 1-bar uptake
**UiO-66**	17.704	1382		
**UiO-66-OS**	13.502	1081	21.78	23.7361422
**UiO-66-PDMS**	7.9995	626	54.703	54.815
**UiO-66-(F)$_4$**	15.417	897		
**UiO-66-(F)$_4$-OS**	11.552	433	51.727	25.068
**UiO-66-(F)$_4$-PDMS**	7.060	500	44.259	54.205
**UiO-66-NO$_2$**	10.919	863		
**UiO-66-NO$_2$-OS**	8.369	567	34.299	23.355
**UiO-66-NO$_2$-PDMS**	5.788	459	46.813	46.993
**UiO-66-NH$_2$**	15.181	1214		
**UiO-66-NH$_2$-OS**	8.206	587	51.648	45.945
**UiO-66-NH$_2$-PDMS**	2.211	76	93.740	85.434

Table S4. Analysis of saturation uptakes (at ≈1 bar) and BET surface areas (SAs) for the phases post-PFAS sorption, compared to the pre-PFAS sorption activated phases of **UiO-66** (green rows), **UiO-66-(F)$_4$** (grey rows), **UiO-66-OS** (orange rows) and **UiO-66-(F)$_4$-OS** (blue rows).

**Sorbent**	**1 bar uptake N_2 (mmol g^{-1})**	**BET SA**	**% reductions in BET SA**	**% reductions in 1-bar uptake**
**UiO-66**	17.70393	1382		
**UiO-66@PFOA**	8.08525	613	55.64399421	54.33076159
**UiO-66@PFOS**	9.028340002	616	55.42691751	49.00375226
**UiO-66@GenX**	2.82454	185	86.61360347	84.04568929
**UiO-66-(F)$_4$**	15.41695	897		
**UiO-66-(F)$_4$@PFOA**	10.11697	669	25.4180602	34.37761684
**UiO-66-(F)$_4$@PFOS**	10.95496	658	26.64437012	28.94210594
**UiO-66-(F)$_4$@GenX**	9.865509998	598	33.33333333	36.00867877
**UiO-66-OS**	13.5017	1081		
**UiO-66-OS@PFOA**	13.5017	1081	0	0
**UiO-66-OS@PFOS**	14.37207	1106	-2.312673451	-6.446373426
**UiO-66-OS@GenX**	15.70534	1137	-5.180388529	-16.32120401
**UiO-66-(F)$_4$-OS**	11.55221	433		
**UiO-66-(F)$_4$-OS@PFOA**	13.99451	566	-30.71593533	-21.14140929
**UiO-66-(F)$_4$-OS@PFOS**	10.55959	501	-15.70438799	8.592468439
**UiO-66-(F)$_4$-OS@GenX**	14.21597	587	-35.56581986	-23.0584451

Table S5. Vibrational modes of adsorbed PFAS chemicals in **UiO-66** and **UiO-66-(F)$_4$**.

Assignment	Frequency Position (cm^{-1})					
	UiO-66			UiO-66-(F)$_4$		
	PFOA	GenX	PFOS	PFOA	GenX	PFOS
ν(C=O)	1650	1650		1660	1660	
$\nu_{as}(CF_2)$	1236	1237	1242	1240	1239	1242
$\nu(CF_2+CF_3)$	1208	1201	1217	1210	1202	1217
$\nu_s(CF_2)$	1156	1156	1156	1146	1158	1149
ν_{as}(C-O-C)		1042			1040	
ν_s(C-O-C)		988	1371			
$\nu_s(SO_3)$			1054			1056

Table S6. Vibrational modes of adsorbed PFAS chemicals in **UiO-66-OS** and **UiO-66-(F)$_4$-OS**.

Assignment	Frequency Position (cm^{-1})					
	UiO-66-OS			UiO-66-(F)$_4$-OS		
	PFOA	GenX	PFOS	PFOA	GenX	PFOS
ν(C=O)	1650	1650		1660	1660	
$\nu_{as}(CF_2)$	1238	1238	1243	1239	1240	1240
$\nu(CF_2+CF_3)$	1208	1200	1217	1209	1202	1217
$\nu_s(CF_2)$	1156	1157	1157	1145	1157	1146
ν_{as}(C-O-C)		1043			1040	
ν_s(C-O-C)		989				
$\nu_s(SO_3)$			1054			1057

References.

[1] J. W. M. Osterrieth, J. Rampersad, D. Madden, N. Rampal, L. Skoric, B. Connolly, M. D. Allendorf, V. Stavila, J. L. Snider, R. Ameloot, J. Marreiros, C. Ania, D. Azevedo, E. Vilarrasa-Garcia, B. F. Santos, X.-H. Bu, Z. Chang, H. Bunzen, N. R. Champness, S. L. Griffin, B. Chen, R.-B. Lin, B. Coasne, S. Cohen, J. C. Moreton, Y. J. Colón, L. Chen, R. Clowes, F.-X. Coudert, Y. Cui, B. Hou, D. M. D'Alessandro, P. W. Doheny, M. Dincă, C. Sun, C. Doonan, M. T. Huxley, J. D. Evans, P. Falcaro, R. Ricco, O. Farha, K. B. Idrees, T. Islamoglu, P. Feng, H. Yang, R. S. Forgan, D. Bara, S. Furukawa, E. Sanchez, J. Gascon, S. Telalović, S. K. Ghosh, S. Mukherjee, M. R. Hill, M. M. Sadiq, P. Horcajada, P. Salcedo-Abraira, K. Kaneko, R. Kukobat, J. Kenvin, S. Keskin, S. Kitagawa, K.-i. Otake, R. P. Lively, S. J. A. DeWitt, P. Llewellyn, B. V. Lotsch, S. T. Emmerling, A. M. Pütz, C. Martí-Gastaldo, N. M. Padial, J. García-Martínez, N. Linares, D. Maspoch, J. A. Suárez del Pino, P. Moghadam, R. Oktavian, R. E. Morris, P. S. Wheatley, J. Navarro, C. Petit, D. Danaci, M. J. Rosseinsky, A. P. Katsoulidis, M. Schröder, X. Han, S. Yang, C. Serre, G. Mouchaham, D. S. Sholl, R. Thyagarajan, D. Siderius, R. Q. Snurr, R. B. Goncalves, S. Telfer, S. J. Lee, V. P. Ting, J. L. Rowlandson, T. Uemura, T. Iiyuka, M. A. van der Veen, D. Rega, V. Van Speybroeck, S. M. J. Rogge, A. Lamaire, K. S. Walton, L. W. Bingel, S. Wuttke, J. Andreo, O. Yaghi, B. Zhang, C. T. Yavuz, T. S. Nguyen, F. Zamora, C. Montoro, H. Zhou, A. Kirchon, D. Fairen-Jimenez, *Adv. Mater.* **2022**, 34, 2201502.

[2] M. R. DeStefano, T. Islamoglu, S. J. Garibay, J. T. Hupp, O. K. Farha, *Chem. Mater.* **2017**, 29, 1357.

[3] Z. Hu, Y. Peng, Z. Kang, Y. Qian, D. Zhao, *Inorg. Chem.* **2015**, 54, 4862.

[4] W. Zhang, Y. Hu, J. Ge, H.-L. Jiang, S.-H. Yu, *J. Am. Chem. Soc.* **2014**, 136, 16978.

[5] X. Qian, F. Sun, J. Sun, H. Wu, F. Xiao, X. Wu, G. Zhu, *Nanoscale* **2017**, 9, 2003.

[6] a)Y. Li, Z. Yang, Y. Wang, Z. Bai, T. Zheng, X. Dai, S. Liu, D. Gui, W. Liu, M. Chen, L. Chen, J. Diwu, L. Zhu, R. Zhou, Z. Chai, T. E. Albrecht-Schmitt, S. Wang, *Nat. Comm.* **2017**, 8, 1354; b)J. A. R. Willemsen, I. C. Bourg, *J. Colloid Interface Sci.* **2021**, 585, 337.

[7] J. J. Wardzala, J. P. Ruffley, I. Goodenough, A. M. Schmidt, P. B. Shukla, X. Wei, A. Bagusetty, M. De Souza, P. Das, D. J. Thompson, C. J. Karwacki, C. E. Wilmer, E. Borguet, N. L. Rosi, J. K. Johnson, *J. Phys. Chem. C* **2020**, 124, 28469.

[8] A. K. Rappe, C. J. Casewit, K. S. Colwell, W. A. Goddard, W. M. Skiff, *J. Am. Chem. Soc.* **1992**, 114, 10024.

[9] M. Salvalaglio, I. Muscionico, C. Cavallotti, *J. Phys. Chem. B* **2010**, 114, 14860.

[10] J. Träg, D. Zahn, *J. Mol. Model.* **2019**, 25, 39.

[11] a)R. J. Woods, R. Chappelle, *J. Mol. Struct.* **2000**, 527, 149; b)T. D. Kühne, M. Iannuzzi, M. D. Ben, V. V. Rybkin, P. Seewald, F. Stein, T. Laino, R. Z. Khaliullin, O. Schütt, F. Schiffmann, D. Golze, J. Wilhelm, S. Chulkov, M. H. Bani-Hashemian, V. Weber, U. Borštnik, M. Taillefumier, A. S. Jakobovits, A. Lazzaro, H. Pabst, T. Müller, R. Schade, M. Guidon, S. Andermatt, N. Holmberg, G. K. Schenter, A. Hehn, A. Bussy, F. Belleflamme, G. Tabacchi, A. Glöß, M. Lass, I. Bethune, C. J. Mundy, C. Plessl, M. Watkins, J. VandeVondele, M. Krack, J. Hutter, *J. Chem. Phys.* **2020**, 152, 194103.

[12] P. Ghosh, Y. J. Colón, R. Q. Snurr, *Chem. Commun.* **2014**, 50, 11329.

[13] K. L. Svane, J. K. Bristow, J. D. Gale, A. Walsh, *J. Mater. Chem. A* **2018**, 6, 8507.

[14] a)G. C. Shearer, J. G. Vitillo, S. Bordiga, S. Svelle, U. Olsbye, K. P. Lillerud, *Chem. Mater.* **2016**, 28, 7190; b)Y. Feng, Q. Chen, M. Jiang, J. Yao, *Ind. Eng. Chem. Res* **2019**, 58, 17646; c)L. Liu, Z. Chen, J. Wang, D. Zhang, Y. Zhu, S. Ling, K.-W. Huang, Y. Belmabkhout, K. Adil, Y. Zhang, B. Slater, M. Eddaoudi, Y. Han, *Nat. Chem.* **2019**, 11, 622.

[15] J. Ribeiro, C. Ríos-Vera, F. Melo, A. Schüller, *Bioinformatics* **2019**, 35, 3499.

[16] A. L. Myers, J. M. Prausnitz, *AlChE J.* **1965**, 11, 121.

[17] A. L. Myers, *AlChE J.* **2002**, 48, 145.

[18] a)F. S. Buarque, C. M. F. Soares, R. L. de Souza, M. M. Pereira, Á. S. Lima, *Chem. Commun.* **2021**, 57, 2156; b)A. Coupé, H. Maskrot, E. Buet, A. Renault, P. J. Fontaine, L. Chaffron, *J. Eur. Ceram.* **2012**, 32, 3837.

[19] S. Sorbara, S. Mukherjee, A. Schneemann, R. A. Fischer, P. Macchi, *Chem. Commun.* **2022**, 58, 12823.
[20] S. Øien, D. Wragg, H. Reinsch, S. Svelle, S. Bordiga, C. Lamberti, K. P. Lillerud, *Cryst. Growth Des.* **2014**, 14, 5370.
[21] X. Esparza, E. Moyano, J. de Boer, M. T. Galceran, S. P. J. van Leeuwen, *Talanta* **2011**, 86, 329.
